# Alkene Epoxidation and Oxygen Evolution Reactions Compete for Reactive Surface Oxygen Atoms on Gold Anodes

**DOI:** 10.1021/jacs.4c08948

**Published:** 2024-12-11

**Authors:** Richa Ghosh, Geoffrey M. Hopping, Jordan W. Lu, Drew W. Hollyfield, David W. Flaherty

**Affiliations:** † School of Chemical and Biomolecular Engineering, Georgia Institute of Technology, Atlanta, Georgia 30332, United States; ‡ Department of Chemical and Biomolecular Engineering, University of Illinois Urbana−Champaign, Urbana, Illinois 61801 United States

## Abstract

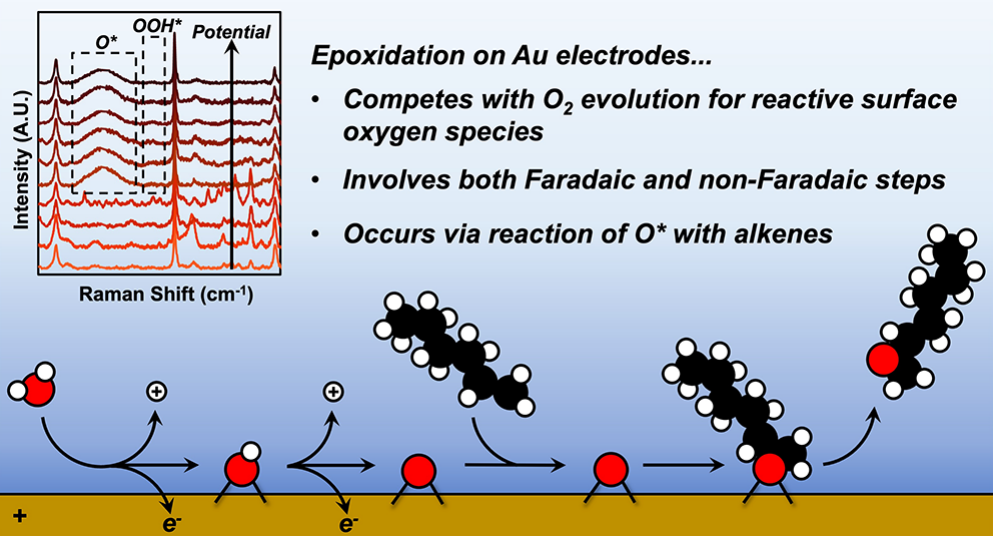

Rates and selectivities for the partial oxidation of organic molecules on reactive electrodes depend on the identity and prevalence of reactive and spectator species. Here, we investigate the mechanism for the epoxidation of 1-hexene (C_6_H_12_) with reactive oxygen species formed by electrochemical oxidation of water (H_2_O) on gold (Au) in an aqueous acetonitrile (CH_3_CN) electrolyte. Cyclic voltammetry measurements demonstrate that oxygen (O_2_) evolution competes with C_6_H_12_ epoxidation, and the Au surface must oxidize before either reaction occurs. *In situ* Raman spectroscopy reveals reactive oxygen species and spectators (CH_3_CN) on the active anode as well as species within the electrochemical double layer. The Faradaic efficiencies toward epoxidation and the ratios of epoxide formation to O_2_ evolution rates increase linearly with the concentration of C_6_H_12_ and depend inversely on the concentration of H_2_O, which agree with analytical expressions that describe rates for reaction between C_6_H_12_ and chemisorbed oxygen atoms (O*) and exclude proposals for other forms of reactive oxygen (e.g., O_2_*, OOH*, OH*). These findings show that the epoxidation and O_2_ evolution reactions share a set of common steps that form O* through electrochemical H_2_O activation but then diverge. Subsequently, epoxides form when O* reacts with C_6_H_12_ through a non-Faradaic process, whereas O_2_ evolves when O* reacts with H_2_O through a Faradaic process to form OOH*, which then deprotonates. These differences lead to distinct changes in rates in response to electrode potential, and hence, disparate Tafel slopes. Collectively, these results provide a self-consistent mechanism for C_6_H_12_ epoxidation that involves reactive O*.

## 1 Introduction

Partial oxidations of alkenes can yield epoxides, such as ethylene and propylene oxide, needed for manufacturing antifreeze, solvents, pharmaceuticals, textiles, and other functional materials.^1−3^ Direct and selective epoxidation with molecular oxygen only occurs with ethylene,^4^ therefore, epoxidations of larger alkenes (e.g., propylene, styrene) utilize aqueous chlorine and calcium hydroxide, organic hydroperoxides, or hydrogen peroxide as coreagents.^5−7^ These thermochemical processes allow for high rates and selectivities but generate large amounts of CO_2_ emissions (0.88 tons CO_2_ per ton ethylene oxide)^8^ and undesired and harmful byproducts (brine and chlorinated compounds),^9^ utilize costly and explosive oxidations,^10,11^ and rely on operation at elevated temperatures or pressures. The reaction of alkenes with oxidants produced via electrochemical routes offers a sustainable alternative to current industrial epoxidation technologies. Indirect electrochemical epoxidations involve the homogeneous reaction of an alkene with an oxidant. The active oxidant, typically active halogens,^12−20^ hydrogen peroxide,^21−24^ or coordination complexes,^25,26^ forms through electrochemical oxidation or reduction of salts or redox species in the electrolyte. Although indirect electrochemical epoxidations can achieve industrially relevant current densities and Faradaic efficiencies, this method results in several drawbacks similar to thermochemical epoxidation processes. These include the production of environmentally harmful halides with the use of active halogens and the need to separate and neutralize oxidant byproducts. The direct, heterogeneous epoxidation of alkenes with water (H_2_O) on an electrode appears as an attractive alternative to thermochemical and indirect electrochemical epoxidation technologies due to the abundance of H_2_O, lack of production of hazardous byproducts, ability to operate at mild conditions, and opportunity to cogenerate hydrogen.^27^ Thermodynamic calculations demonstrate that the epoxidation of alkenes with H_2_O occurs at anodic potentials greater than 0.8 V versus the standard hydrogen electrode (*V*_SHE_) at ambient conditions.^28^ Transition metals, including Au,^29^ Pd,^30−36^ Pt,^35,37−39^ Ag,^40−42^ Mn,^43−45^ and Ru,^46−48^ oxidized through electrochemical, thermal, or chemical treatment catalyze alkene epoxidations at oxidizing potentials. The requirement for an oxidized catalyst implies that the alkene reacts with a surface bound oxygen species to form an epoxide molecule. The identities of the reactive oxygen species remain challenging to demonstrate despite the need for this knowledge to inform the design of increasingly reactive and selective epoxidation catalysts.

Proposed mechanisms for alkene epoxidations in electrochemical systems involve reactive oxygen surface intermediates also formed during the oxygen (O_2_) evolution reaction (OER). In the commonly invoked mechanism for OER, which involves nucleophilic attack of H_2_O to a terminal oxo (O*), two H_2_O molecules react through four sequential proton–electron transfer steps to create four oxygen-containing surface species that include hydroxyl (OH*), O*, hydroperoxo (OOH*), and peroxo (O_2_*), which reduces and desorbs to form gaseous O_2_.^49−51^ Studies agree that one of these four oxygen-containing surface species must form electrochemically through H_2_O activation before reacting, either electrochemically or chemically, with alkenes to form epoxides. However, there remains a lack of consensus regarding the identity of the reactive oxygen species among studies on similar catalysts and electrolyte systems. For example, proposals for the mechanism of propylene epoxidation on Ag-based electrocatalysts invoke different forms of reactive species (OH*,^42^ O*^40^) despite operating in similar electrolytes (0.1 M phosphate buffer solution, saturated propylene, H_2_O). Ke et al. concluded that propylene reacts with OH* (and not O*) to form propylene oxide on crystalline Ag_3_PO_4_ particles immersed in an aqueous electrolyte (0.1 M phosphate buffer solution, saturated propylene, H_2_O), based on differences between apparent energy barriers for competing pathways computed by density functional theory (DFT, solid-vacuum interface).^42^ In comparison, recent work on V-doped AgO (0.1 M phosphate buffer solution, saturated propylene, H_2_O) posits reactive O* species transfer to propylene, because *in situ* Raman and infrared spectroscopy show that Ag surfaces covered in O* and O_2_* promote propylene epoxidation and overoxidation to CO_2_, respectively.^40^ Several groups propose ethylene and propylene undergo direct oxidation to their respective glycols via metal hydroxides formed on Pd and Pd-based bimetallics (Pd–Au, Pd–Rh), based on DFT simulations together with catalyst testing and *in situ* spectroscopy (Raman, infrared) in various electrolytes (0.1 M sodium perchlorate, 0.1 M perchloric acid, saturated alkene, H_2_O) and pH (pH 3–10).^31,32,36^ In this mechanism, alkenes adsorb to the surface and react successively with two lattice OH* to form glycols in steps that resemble a Mars-van Krevelen process. In contrast, Chung et al. combined electrochemical kinetics, rate measurements, and kinetic modeling to demonstrate that propylene undergoes epoxidation to propylene oxide by reactions with O_2_* on oxidized PdPt_*x*_ nanoparticles in aqueous (0.25 M sodium phosphate buffer, 0.9 M NaClO_4_, 0.1–1 atm propylene, H_2_O, pH 6) and aqueous–organic (0.1 M tetrabutylammonium tetrafluoroborate, 2–5 M H_2_O, 0.1–1 atm propylene, acetonitrile (CH_3_CN)) electrolytes.^35^ Multiple forms of experimental evidence excluded pathways in which propylene reacts with OH*, O*, or OOH*. Here, propylene glycol forms at higher amounts in the aqueous electrolyte and exclusively forms in acidic electrolytes (0.1 M perchloric acid) due to a secondary acid-catalyzed hydrolysis of epoxides to glycols^52,53^ and not by direct reaction of propylene with OH* or other species. In contrast to investigations with platinum group metals, cyclohexene was reported to react with OOH* to produce the corresponding epoxide on MnO_2_ nanoparticles in aqueous–organic electrolyte (0.1 M tetrabutylammonium tetrafluoroborate, 5 M H_2_O, CH_3_CN), as indicated by the stereochemistry of products formed from *cis*-stilbene epoxidation and *in situ* Raman spectra that detected Mn–OOH.^45^ This contradicts conclusions from contemporaneous studies, which gave experimental evidence that cyclooctene reacts with O* to form the corresponding epoxide on Mn_3_O_4_^43^ and Ir-decorated MnO_*x*_^44^ nanoparticles in aqueous–organic electrolytes (0.11 M tetrabutylammonium tetrafluoroborate, 1–15 M H_2_O, 0.001–0.2 M cyclooctene, CH_3_CN). Recent articles suggest that alkenes react with O* species to produce epoxides over Au^29^ (0.1 M tetrabutylammonium perchlorate, 5 M H_2_O, 0.1 M cyclooctene, CH_3_CN), PtO_*x*_^37^ (dichloromethane, saturated propylene), and Ru_2_O/N_0.12_C^46^ (0.05 M lithium perchlorate, 41 M H_2_O, 0.025 M cyclooctene, ethanol) catalysts, however, these proposals draw support from recognition that epoxidations equate to two electron oxidations of the alkene and O* forms on anodes via two proton–electron transfer steps during OER. Overall, a collection of experimental and computational studies indicates that the reactive oxygen species for epoxidations vary among catalysts. Yet, the literature includes significant disagreement on the identity of these active oxygen species and the mechanism of these reactions, due in part to a scarcity of kinetic measurements. Further development of electrocatalysts for selective epoxidations will require thorough understanding of the identities of the reactive oxygen species and abundance of other oxygen species on different metal catalysts.

Knowledge of the dominant surface species and the distinctions between reactive and spectating species provide evidence to support or exclude proposed reaction mechanisms and guide the design of materials with greater rates and selectivities. Certain forms of *in situ* spectroscopy, and surface enhanced Raman spectroscopy (SERS) in particular, possess the surface selection rules and sensitivity required to detect surface intermediates pertinent for OER,^54−56^ CO_2_ reduction,^57−59^ oxygen reduction,^60,61^ and nitrate reduction.^62,63^ Features in Raman spectra that represent the O–O stretching mode of OOH* have been observed during OER on Au,^54,64−66^ Ni,^67−69^ and Co^70,71^ in alkaline and neutral electrolytes. *In situ* Raman spectroscopy shows that Au (single-crystal),^72^ Pt (single-crystal),^73^ and Cu (single-crystal, polycrystalline)^74^ surfaces oxidize and reconstruct with applied electrochemical potentials, which agrees with interpretation of voltammetry measurements. A single investigation used *in situ* SERS to examine reactive surface species during alkene epoxidations on anodes and found that Ag-based catalysts contain O* and O_2_* under propylene epoxidation conditions (0.1 M phosphate buffer solution, saturated propylene).^40^

Here, we combine electrochemical and catalytic rate measurements together with *in situ* Raman spectroscopy to elucidate the mechanism for the epoxidation of 1-hexene (C_6_H_12_) with H_2_O-derived oxygen species to 1,2-epoxyhexane (C_6_H_12_O) and characterize the structure and active species upon an Au anode in an aqueous–organic electrolyte (H_2_O, CH_3_CN, and tetrabutylammonium perchlorate). Electrochemical rate measurements obtained over a span of C_6_H_12_ and H_2_O concentrations ([C_6_H_12_], [H_2_O]) show that C_6_H_12_ epoxidation occurs only after the Au surface oxidizes. Moreover, decreases in electrochemical rates with increasing [C_6_H_12_] at oxidizing potentials show that C_6_H_12_ epoxidation and OER compete for an identical pool of reactive surface oxygen species derived from H_2_O. *In situ* Raman spectroscopy measurements confirm that the initially metallic Au anode reconstructs at relevant potentials to form Au oxide prior to epoxidation and O_2_ evolution, as signified by the appearance of Au-bound O* species. Epoxide formation rates and epoxidation Faradaic efficiencies depend on the applied potential in ways that suggest the epoxidation and OER mechanism compete for reactive oxygen species and that epoxidation does not occur via only Faradaic steps. We derive rate laws for epoxidation and OER that involve formation of epoxide by reactions with distinct reactive oxygen species (O*, OH*, OOH*, O_2_*). The dependencies of measured C_6_H_12_O and O_2_ formation rate ratios and epoxidation Faradaic efficiencies on reagent concentrations agree only with analytical expressions that describe the epoxidation of C_6_H_12_ by reaction with an O* species and exclude mechanisms in which OH*, OOH* or O_2_* react with C_6_H_12_. This conclusion appears consistent with product distributions from *cis*-stilbene epoxidation, radical clock experiments, and with Tafel slopes for epoxidation and OER. Collectively, these findings provide insight on the mechanism and identity of the reactive oxygen surface species responsible for epoxidation on Au.

## 2 Experimental Methods

### 2.1 Chemicals and Reagents

1-Hexene (C_6_H_12_, Sigma-Aldrich, ≥99%), 1,2-epoxyhexane (C_6_H_12_O, Thermo Fisher Scientific, 97%), *cis*-stilbene (Sigma-Aldrich, 96%), 2-hexanone (Sigma-Aldrich, reagent grade, 98%), valeraldehyde (Sigma-Aldrich, 97%), hexanal (Sigma-Aldrich, 98%), 1-hexanol (Sigma-Aldrich, anhydrous, ≥99%), 1,2-hexanediol (Sigma-Aldrich, 98%), 2-hexanol (Sigma-Aldrich, 99%), *cis*-stilbene oxide (Sigma-Aldrich, 97%), *trans*-stilbene oxide (Sigma-Aldrich, 98%), decane (Sigma-Aldrich, ReagentPlus, ≥99%), tetrabutylammonium perchlorate (TBAClO_4_, TCI America, 98%), acetonitrile (CH_3_CN, Sigma-Aldrich, HPLC, ≥99%), sodium perchlorate (NaClO_4_, Sigma-Aldrich, ACS reagent, ≥98%), potassium hydroxide (KOH, Sigma-Aldrich, ACS reagent, ≥98%), ferrocene (Fc, Sigma-Aldrich, 98%), isotope labeled water (H_2_^18^O, Cambridge Isotope Laboratories, 97%) and hydrogen peroxide (H_2_O_2_, J. T. Baker, 30%, ULTREX II, Ultrapure for trace metal analysis) were purchased and used without additional treatment. Water from a Milli-Q benchtop lab water purification system (Milli-Q EQ 7016 Ultrapure Water Purification System) was used.

### 2.2 Reaction Rate Measurements

#### 2.2.1 Electrochemical Kinetics Using Cyclic Voltammetry

Cyclic voltammetry measurements were carried out in a sandwich-type one-compartment electrochemical flow cell (Figure S1a). The cell was constructed of polyetheretherketone (PEEK) and contained a flow path with a geometric area of one cm^2^. Polycrystalline Au (Goodfellow, 99.999%) and Pt (Thermo Scientific, 99.99%) foils were used as the working and counter electrodes, respectively, and a leak-free silver–silver chloride electrode (Ag/AgCl, Innovative Instruments, 3.4 M KCl leak-free 1.6 mm diameter) was used as the pseudoreference electrode. A potentiostat (Biologic, VSP-3e) equipped with capabilities for potentiostatic electrochemical impedance spectroscopy (EIS) was connected to the reference electrode and stainless-steel plates that served as current collectors for the working and counter electrodes. The flow cell was oriented vertically to allow for upward liquid flow across the electrodes (Figure S1b). The upward electrolyte flow prevented the accumulation of O_2_ on the working electrode. The electrolyte solution (55 cm^3^) with the desired concentrations of C_6_H_12_ and H_2_O (0.1 M TBAClO_4_, CH_3_CN) was recirculated (10 cm^3^ min^–1^) through the cell and an external electrolyte reservoir using a high-performance liquid chromatography pump (Chrom Tech, HPLC, M1) with interior PEEK lining. The electrolyte reservoir was a sealed, media bottle (Pyrex, 100 cm^3^) in which the electrolyte was stirred (400 rpm) to maintain a homogeneous solution. PEEK tubing, ferrules, and nuts were used for all connections between the electrolyte reservoir, pump, and flow cell.

Prior to cyclic voltammetry measurements, the Au foil anode was cleaned *in situ* with cyclic voltammetry (20 cycles, −0.44 to 1.66 V_Fc/Fc+_, 50 mV s^–1^) with a cleaning electrolyte solution (0.1 M TBAClO_4_, 10 M H_2_O, CH_3_CN) with the intent to remove organic surface contaminants (Figure S2). The system was then placed in a single-pass flow configuration and the cleaning electrolyte solution was replaced with the electrolyte solution (75 cm^3^) containing the desired concentrations of C_6_H_12_ and H_2_O. Using the replaced electrolyte solution, the system was flushed (20 cm^3^) to remove the cleaning electrolyte from the flow cell and pump. The system was then returned to the recirculating flow configuration for cyclic voltammetry measurements. The ranges of concentrations studied for C_6_H_12_ (0–0.32 M C_6_H_12_) and H_2_O (0–18 M H_2_O) were limited by the solubility of C_6_H_12_ in the aqueous–organic electrolyte. The solution resistance value at open circuit potential (OCP) was determined by EIS and analysis of the resulting Nyquist plots. All reported potentials were fully compensated for resistive losses (i.e., 100% *iR* compensated) unless otherwise noted. Electrochemical surface area measurements were taken before each measurement (see Section S3 for further details). All current densities are normalized by the anode geometric area (1 cm^2^). For cyclic voltammetry measurements, three cyclic voltammetry cycles (−0.54 to 1.46 V_Ag/AgCl_, 10 mV s^–1^) were run to ensure that the cyclic voltammograms converged and the surface was stable (Figure S4). The reported cyclic voltammograms are the third cycle collected. All potentials taken in aqueous–organic and aqueous electrolyte are reported versus ferrocene/ferrocenium (Fc/Fc^+^) and reversible hydrogen electrode (RHE), respectively (see Section S5 for details).

#### 2.2.2 Product Formation Rate Measurements with Chronoamperometry and Gas Chromatography

Measurements of epoxide formation rates and electrode currents were carried out over extended periods (22–48 h) at fixed potentials (chronoamperometric) and concentrations of C_6_H_12_ and H_2_O ([C_6_H_12_], [H_2_O]) at ambient temperature in the apparatus described in Section 2.2.1. The hydrogen evolution reaction occurred at the Pt cathode. Prior to rate measurements, the anode was cleaned, and the solution resistance measured. Three cycles of cyclic voltammetry (−0.54 to 1.46 V_Ag/AgCl_, 10 mV s^–1^) were performed to confirm the reproducible preparation of the anode.

Liquid aliquots (1.0 cm^3^), including a sample taken before the chronoamperometry measurement began, were taken with a syringe equipped with a stainless-steel needle throughout the chronoamperometry measurement to quantify the amount of C_6_H_12_O formed and C_6_H_12_ consumed. Liquid–liquid extraction was performed by combining the electrolyte aliquot with decane (1.5 cm^3^) in a disposable glass culture tube where it was agitated (10 s) with a plastic spatula and then allowed to settle (5 min) with the intent to recover organic components of the electrolyte solution while preventing TBAClO_4_ from being introduced to the gas chromatograph. A portion of the decane phase (0.5 cm^3^) was pipetted into a chromatography autosampler vial and later injected, with an autosampler (Agilent 7693A), to a gas chromatograph (Agilent, 8890) equipped with a flame ionization detector and a polysiloxane column (Agilent, HP-1, 19091Z-115E). The concentrations of C_6_H_12_ and C_6_H_12_O were determined with calibration curves (Figure S7) obtained by application of an identical sample preparation and analysis method to samples with known [C_6_H_12_] and [C_6_H_12_O]. The GC analysis method displays an uncertainty of ∼5% (Figure S8). Product analysis from the chronoamperometry measurements with H_2_^18^O was done with gas chromatography–mass spectrometry (GC-MS, Thermo Trace 1610/ISQ 7610, Agilent HP-5MS UI column) measurements carried out by staff scientists at the Parker H. Petit Institute for Bioengineering and Bioscience’s Systems Mass Spectrometry Core at Georgia Tech.

Epoxidation rates were determined by plotting the amount of epoxide formed versus time, with the amount of epoxide formed fixed at zero at time equal to zero, and taking a linear fit. The carbon balance closes within 95–115% for all reported measurements. Side reactions and further oxidation reactions did not form concentrations of products detectable in the gas chromatograms (see Section S7). Analysis of these data shows that C_6_H_12_O forms at a consistent rate throughout the 22 to 48 h period of each experiment (Figure S12), which indicates that secondary reactions that consume the epoxide do not occur at measurable rates. All reported epoxidation and O_2_ evolution rates were measured at differential conversion (< 0.1%) with respect to C_6_H_12_ and H_2_O and reactant depletion did not influence the observations. Replicate measurements indicate that reported rates have an uncertainty of ∼20%. The Faradaic efficiency (FE) at each time point was calculated using the following equation:
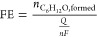
1where  is the moles of epoxide formed determined through gas chromatography analysis, *Q* is the cumulative charge passed determined through integration of the chronoamperogram (Figure S13), *F* is the Faraday constant (96,485 C mol^–1^), and *n* is the number of electrons required for the production of one C_6_H_12_O molecule (two e^–^). Reported values of FE represent averages of FE calculated throughout each experiment, which differed by less than 10% (Figure S12). It was assumed that the remaining amount of charge went toward OER (i.e., no other side reactions occur). Section S8 further describes calculations for epoxidation and O_2_ evolution partial currents and rates. Replicate experiments demonstrate an uncertainty of ∼15% for reported FE values.

The conditions used for rate measurements here avoid external mass transfer constraints, because current densities (0.01–1 mA cm^–2^) do not depend on flow rate across the range of flow rates (7–10 cm^3^ min^–1^) studied (Figure S14).^75^ In addition, epoxidation rates exhibit first- and zero- order dependence on [C_6_H_12_] and [H_2_O], respectively, at conditions studied (Section 3.3). A system with external mass transfer limitations would show a sublinear dependence on reaction concentrations because the mean concentration of the reactant throughout the diffusion layer would not depend linearly on the fluid phase reactant concentrations for a zero-order reactant.^76^

Au content in the electrolyte before and after chronoamperometry and cyclic voltammetry measurements was determined by *ex-situ* analysis of the electrolyte with inductively coupled plasma optical emission spectroscopy (ICP-OES, PerkinElmer, Optima 73000DV ICP-OES) measurements carried out by staff scientists at the Pulping and Chemical Analytical Lab at Georgia Tech and UV–vis spectroscopy obtained with a mini transmission dip probe (Avantes, Hastelloy). Au deposition on the pump inlet filter after chronoamperometry and cyclic voltammetry measurements was determined by *ex-situ* analysis of the filter with energy-dispersive X-ray fluorescence spectroscopy (EDXRF, Bruker, M4 Tornado).

### 2.3 *In Situ* Raman Spectroscopy

*In situ* Raman spectroscopy was used to probe the state of the Au anode and examine surface intermediates. The polycrystalline Au foil was placed in an undivided electrochemical Raman cell (Figure S15a). A stainless-steel plate sputter-coated with Pt (150 nm) was used as the cathode and an Ag/AgCl electrode (BASi, 3 M KCl, 7.5 cm length, 6 mm diameter) placed downstream of the Raman cell was used as the reference electrode. Aqueous potassium chloride solution (1 M KCl, H_2_O) was recirculated (1 cm^3^ min^–1^) through the Raman cell using an HPLC pump (Chrom Tech, M1, PEEK). Prior to the *in situ* Raman measurements, the Au foil was electrochemically roughened following a previously reported method to create Au nanoparticles on the surface so the surface-enhanced Raman effect could be induced.^77^ First, the system was held at reducing potentials to desorb any adsorbates (−1.16 V_Ag/AgCl_, 10 min) and initiate Au dissolution (−0.06 V_Ag/AgCl_, 2 min). Then cyclic voltammetry was performed (−0.06 to 1.44 V_Ag/AgCl_, hold at −0.06 V_Ag/AgCl_ for 30 s and 1.44 V_Ag/AgCl_ for 2 s, 750 mV s^–1^, 20 cycles) to dissolute Au and then redeposit it on the surface. Finally, the Au foil was returned to a reducing potential (−0.36 V_Ag/AgCl_, 2 min) to desorb residual surface chlorine. The Au foil was rinsed in the Raman cell by flowing deionized H_2_O (100 cm^3^), then H_2_O_2_ (30%, 100 cm^3^) to remove organic contaminants on the Au surface, and then deionized H_2_O again (500 cm^3^). The electrochemical roughening process produces a surface that contains ∼100 nm nodules, which results in surface enhanced Raman scattering similar to Au nanoparticles.^78^

Raman measurements were carried out with electrolyte flow (1 cm^3^ min^–1^) in a single-pass configuration (Figure S15b). A Raman spectrometer (Renishaw, inVia) equipped with a 632.8 nm diode laser was used to perform the measurements. Prior to Raman measurements, the anode was cleaned, and the solution resistance was measured (Section 2.2.1). The system was held at the desired potential before (5 min) and during the spectra collection. Spectra were taken at different positions on the Au surface and different potentials in random order (i.e., not decreasing or increasing potential). Spectra were obtained with a long 50× objective and using a “scan edge” feature that disperses the light across an area of the electrode of ∼10 μm^2^, which leads to a power density of approximately 1.4 mW μm^–2^ at the surface of the sample (Gentech-EO, PRONTO-SI). High resolution spectra are averaged from 20 individual acquisitions with a scan time of 30 s per acquisition, and extended scans represent the average of 10 acquisitions of 20 s. Reported spectra are normalized by the most intense feature in the spectra. In these spectra, a defective element on the charge coupled device detector gives a false inverse feature at 595 cm^–1^, which appears in all spectra including control experiments. This inverse feature is removed using software corrections described in Section S11.

### 2.4 Measurements of the Thermodynamic Activity of Reagents

The thermodynamic activities of H_2_O and C_6_H_12_ across a range of different electrolyte compositions (Figure S18) were measured using an adaptation of a similar method.^79^ Samples of electrolyte solutions (4 cm^3^, 0.1 M TBAClO_4_, CH_3_CN) with the desired H_2_O and C_6_H_12_ concentrations were prepared and placed in headspace vials (Agilent, 20 cm^3^, crimp top, silver aluminum cap, PTFE/silicone septa). The vials were sealed and left at ambient conditions overnight (at least 12 h) to allow the system to achieve vapor–liquid equilibrium. The partial pressure of H_2_O in the vial was quantified with a gas chromatograph (Agilent, 7890) equipped with a headspace autosampler (Agilent, G1888A) and polydimethylsiloxane column (Agilent, DB-624UI, 123-1334UI) connected to a thermal conductivity detector. The partial pressure of C_6_H_12_ in the headspace was quantified using a second channel on the same apparatus, which was equipped with a polysiloxane column (HP-1, Agilent, 19091Z-115E) connected to a flame ionization detector. Activity measurements for each electrolyte composition were collected in triplicates, and compositions were measured in randomized order. During the collection of H_2_O activity measurements, a vial of pure H_2_O was run before each electrolyte sample to serve as a reference for activity calculation and to minimize the effects of fluctuations in ambient temperature. Similarly, during the collection of C_6_H_12_ activity measurements, a vial of pure C_6_H_12_ was run after every four samples of electrolyte to serve as a reference for the next four activity measurements.

The thermodynamic activity coefficient (γ_*i*_) of H_2_O and C_6_H_12_ for each electrolyte composition was calculated, with the assumption that the integrated peak area for each species in the gas chromatogram is proportional to its partial pressure in the vial, using the following equation:
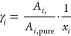
2where *A*_*i*_ is the integrated peak area of species *i* (either H_2_O or C_6_H_12_), *A*_*i,*pure_ is the peak area obtained from the headspace of vial containing only species *i*, and *x*_*i*_ is the mole fraction of species *i* in the electrolyte. The derivation of eq 2 from Raoult’s Law appears in Section S12.

Thermodynamic activities (*a*_*i*_) of each species were then calculated with:
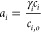
3where *c*_*i*_ is the concentration of species *i* in the electrolyte and *c*_*i,o*_ is the standard state concentration (i.e., the concentration of the pure species). The activity coefficient of the pure species is equal to unity.

## 3 Results and Discussion

### 3.1 Effects of [C_6_H_12_] and [H_2_O] on Rates of Electrochemical Processes

**Figure 1:**
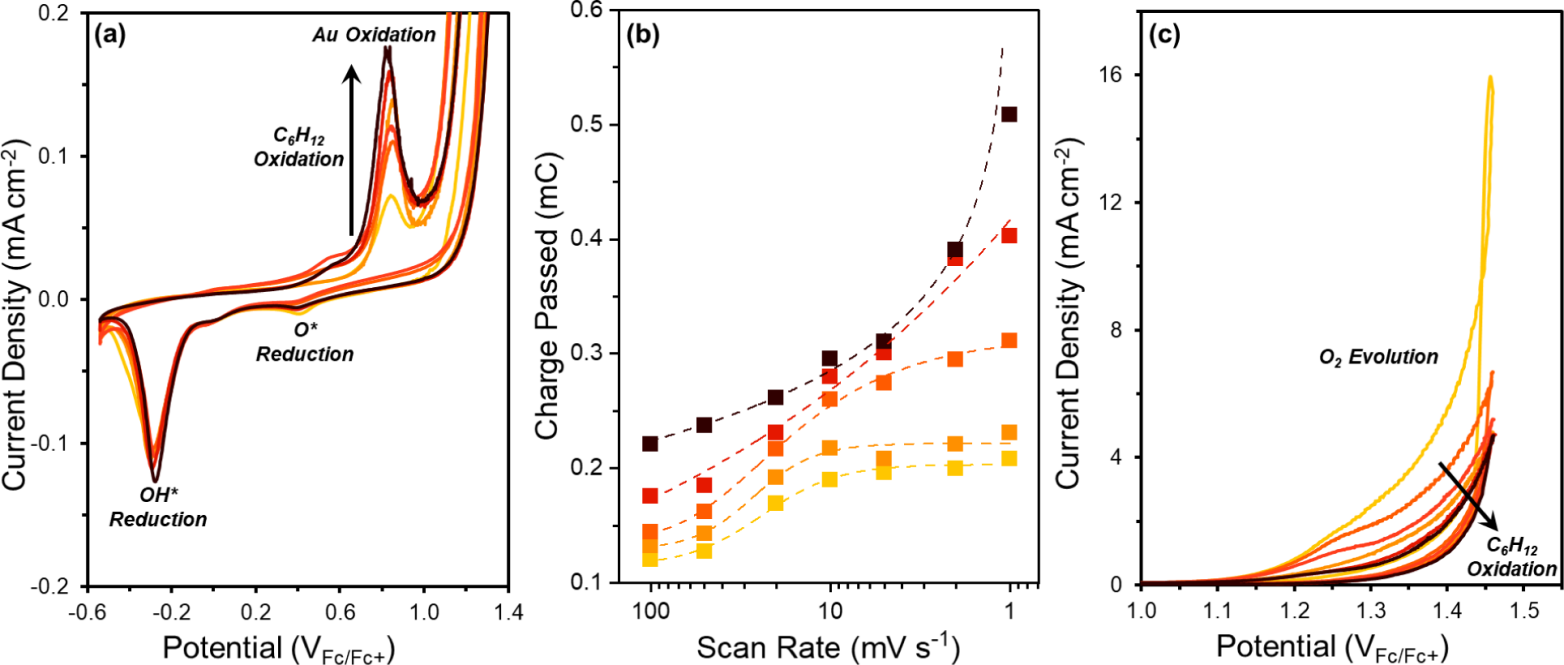
(a) Cyclic voltammograms scaled to show features between −0.6 to 1.4 V_Fc/Fc+_ as functions of [C_6_H_12_] (0, 0.001, 0.01, 0.05, 0.1, 0.32 M C_6_H_12_) on Au (0.1 M TBAClO_4_, 10 M H_2_O, CH_3_CN, 10 mV s^–1^). (b) Charge passed from the oxidation feature at 0.65 to 0.94 V_Fc/Fc+_ as a function of scan rate (100, 50, 20, 10, 5, 2, 1 mV s^–1^) in electrolytes of varying [C_6_H_12_] (0, 0.001, 0.01, 0.1, 0.32 M C_6_H_12_) on Au (0.1 M TBAClO_4_, 10 M H_2_O, CH_3_CN). (c) Cyclic voltammograms scaled to show features between 1.0 to 1.5 V_Fc/Fc+_ as functions of [C_6_H_12_] (0, 0.001, 0.01, 0.05, 0.1, 0.32 M C_6_H_12_) on Au (0.1 M TBAClO_4_, 10 M H_2_O, CH_3_CN, 10 mV s^–1^). Yellow data series represent measurements in the absence of C_6_H_12_, while higher [C_6_H_12_] appear as increasingly dark orange and red series.

Cyclic voltammetry measurements as a function of [C_6_H_12_] and [H_2_O] provide insight into the onset potential of C_6_H_12_ oxidation and other surface oxidation processes that occur on Au through analysis of their electrochemical rates (i.e., current). Figure 1 shows cyclic voltammograms conducted across a range of [C_6_H_12_] (0–0.32 M C_6_H_12_), which demonstrate that multiple distinct oxidation and reduction processes occur on the Au anode. In the absence of C_6_H_12_, oxidation features from 0.65 to 0.94 V_Fc/Fc+_ on the anodic scan signify oxidation and reconstruction of the metallic Au surface to an Au surface oxide. An increasing coverage of oxygen species form as H_2_O molecules activate upon the anode as the electrode potential increases (e.g., Au, Au(OH)_3_, and Au_2_O_<3_). Subsequently, the OER occurs at 0.94 V_Fc/Fc+_ and greater potentials. Two reduction features on the cathodic scan (−0.5 to −0.05 V_Fc/Fc+_, 0.2 to 0.6 V_Fc/Fc+_) reflect reduction of the surface Au_2_O_<3_ to Au(OH)_3_ and Au(OH)_3_ to metallic Au, respectively.^54,80^

Oxidative current densities increase with values of [C_6_H_12_] (Figure 1a, 0.4 to 0.94 V_Fc/Fc+_). The oxidative current densities from 0.4 to 0.65 V_Fc/Fc+_ increase with the introduction of C_6_H_12_ in the electrolyte but do not change as a function of increasing [C_6_H_12_]. This increase in current density results from C_6_H_12_ adsorption on metallic Au.^81^ Au surface oxidation above 0.65 V_Fc/Fc+_ inhibits C_6_H_12_ adsorption.^82^ From 0.65 to 0.94 V_Fc/Fc+_, the oxidative current densities increase with [C_6_H_12_], which indicates that C_6_H_12_ reacts with surface oxygen species formed by the activation of H_2_O on Au. This process generates an oxygen vacancy and allows for additional H_2_O molecules to activate on the surface. The Au surface must first activate H_2_O and accumulate oxygen species prior to C_6_H_12_ oxidation, evidenced by current densities that remain constant across [C_6_H_12_] values until the onset of Au surface oxidation at 0.65 V_Fc/Fc+_. As [C_6_H_12_] increases from 0 to 0.1 M C_6_H_12_, the total charge passed in the oxidation feature (0.65 to 0.94 V_Fc/Fc+_), calculated by integration of current with respect to time, increases by a factor of three (Figure S19a). Here, the increase in charge passed reflects that greater number of oxygen atoms derived from H_2_O to oxidize both the C_6_H_12_ and Au surface. The total charge passed between 0.65 to 0.94 V_Fc/Fc+_ does not change significantly when [C_6_H_12_] increases beyond 0.1 M C_6_H_12_, likely due to limitations on the quantity of C_6_H_12_ that can diffuse to the surface within the time scale of the oxidation feature at the set scan rate (10 mV s^–1^).

Cyclic voltammetry measurements with various scan rates further support that C_6_H_12_ oxidizes through reaction with surface oxygen species at potentials greater than 0.94 V_Fc/Fc+_. Figure 1b shows that charge passed in the oxidation feature (0.65 to 0.94 V_Fc/Fc+_) depends on both voltammetric scan rate and [C_6_H_12_], and more importantly, the total charge passed continues to increase over a wider range of [C_6_H_12_] at the slowest scan rate (1 mV s^–1^). For reactions stoichiometrically limited by the number of surface sites (e.g., Au surface oxidation in the absence of C_6_H_12_), the charge passed initially increases with decreasing scan rate, because the concentration gradient near the electrode decreases and the surface reaction approaches full completion prior to the onset of OER.^83^ In contrast, the charge passed continually increases with decreasing scan rate in the presence of sufficient quantities of C_6_H_12_, which consume reactive surface oxygen species and circumvent the previous limitations imposed when H_2_O stoichiometrically oxidizes only the accessible surface sites. These interpretations agree with complementary scans to more reducing potentials that show the total charge passed in reduction features do not depend on [C_6_H_12_] (Figure S19b), which indicates that potential-induced adsorption and desorption of C_6_H_12_ does not occur at measurable rates. This provides additional support to C_6_H_12_ oxidation through a nonstoichiometric surface limited reaction with H_2_O-derived surface oxygen species.

**Figure 2:**
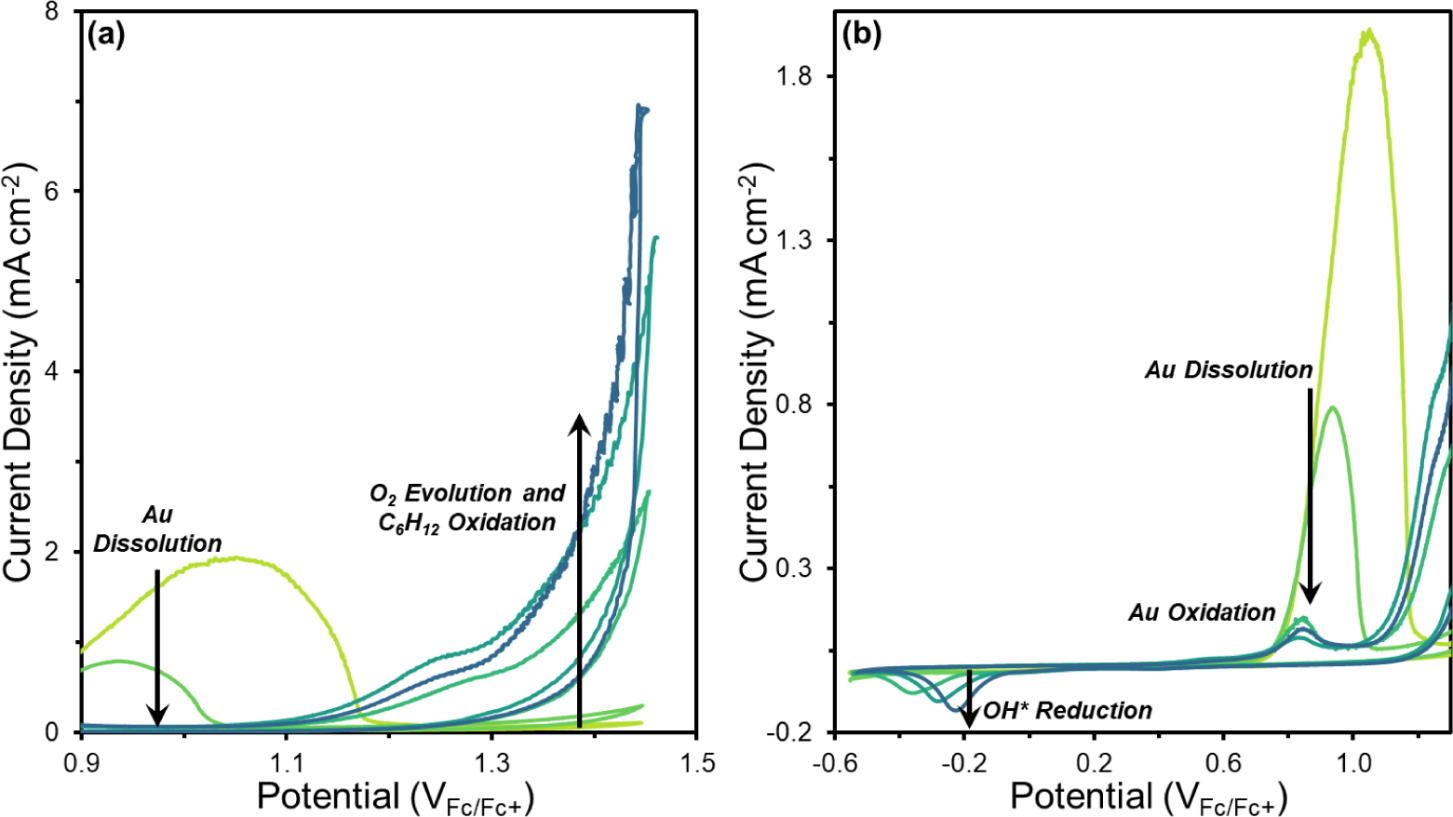
Cyclic voltammograms performed across a range of [H_2_O] (0, 1, 5, 10, 15 M H_2_O) on Au (0.1 M C_6_H_12_, 0.1 M TBAClO_4_, CH_3_CN, 10 mV s^–1^) rescaled to clearly show features between (a) 0.9 to 1.5 V_Fc/Fc+_, and (b) –0.6 to 1.3 V_Fc/Fc+_. Light green data series represent measurements in the absence of [H_2_O] and increasing values of [H_2_O] appear as increasingly dark blue-green and blue series.

Figure 1c demonstrates that the presence of C_6_H_12_ inhibits O_2_ evolution, because current densities decrease substantially with increasing [C_6_H_12_] (0–0.32 M C_6_H_12_) at potentials greater than 0.94 V_Fc/Fc+_. These trends implicate that C_6_H_12_ reacts with surface intermediates that also participate in processes for O_2_ formation from the oxidized Au surface and, furthermore, the reactions with C_6_H_12_ (e.g., oxidation) possess a weaker dependence on electrode potential than OER. The current density does not change significantly across values of [C_6_H_12_] greater than 0.1 M C_6_H_12_ (Figure S19c), consistent with the proposal that C_6_H_12_ reacts at the electrode surface. Furthermore, Figure 2a shows that current densities increase an order of magnitude from 0 to 5 M H_2_O at potentials greater than 1.2 V_Fc/Fc+_ (Figure S20a), which suggests both C_6_H_12_ oxidation and OER involve reactive oxygen species derived from H_2_O. Collectively, these results show that C_6_H_12_ oxidation and the O_2_ evolution reaction share a common pool of reactive oxygen surface species derived from electrochemical H_2_O activation before the reaction pathways diverge to form their respective products.

The stability of the polycrystalline Au foil depends strongly on the concentrations of H_2_O and CH_3_CN in the electrolyte solution. Figure 2b presents cyclic voltammograms collected across a range of [H_2_O] (0–15 M H_2_O) that show an unexpected increase in the total charge passed during oxidation of Au by H_2_O (positive current features) along with a decrease in reduction features of Au oxides (negative current features) as values of [H_2_O] decrease. The trends on oxidation and reduction features demonstrate that Au undergoes oxidation but also dissolution as values of [H_2_O] decrease. The Au oxidation feature (0.65 to 1.18 V_Fc/Fc+_) increases sharply with decreasing [H_2_O] at values less than 5 M H_2_O (Figure S20b). As discussed above, this feature represents the formation of a surface oxide layer, and the increase in charge passed signifies the dissolution of Au^n+^ followed by oxidation and dissolution of the previously subsurface Au atoms. Continued cyclic voltammetry or constant potential electrolysis at low [H_2_O] (< 5 M H_2_O) forms purple deposits upon the pump filter and discoloration of the electrolyte (Figure S21). ICP-OES, EDXRF, and UV–vis spectroscopy corroborate the corrosion of the Au anode and the formation of Au nanoparticles (50–100 nm) within the electrolyte (Section S14.1). The Au surface oxidation feature (0.65 to 0.94 V_Fc/Fc+_) remains constant with [H_2_O] at concentrations greater than 5 M H_2_O, presumably due to displacement of organic species from the surface by H_2_O and H_2_O-derived oxygen species. Consequently, rates of C_6_H_12_ oxidation (*vide infra*) were measured only in electrolytes with at least 5 M H_2_O (see Section S14.2 for discussion regarding lack of Au dissolution at high [H_2_O]).

Collectively, these cyclic voltammograms reveal that the oxidized Au surface catalyzes C_6_H_12_ oxidation and O_2_ evolution and corrodes (in certain cases) at rates that depend on [H_2_O]. The oxidation of C_6_H_12_ proceeds once sufficient coverages of reactive oxygen accumulate on Au, however, O_2_ evolution requires higher oxygen coverages and greater electrode potentials. These findings also indicate that C_6_H_12_ oxidation and O_2_ formation compete for a common pool of reactive surface oxygen species. We next utilize *in situ* surface enhanced Raman spectroscopy to characterize how the species located on and near the Au surface depend on electrolyte composition and electrode potential.

### 3.2 Characterization of Species on and Near the Au Surface

**Figure 3:**
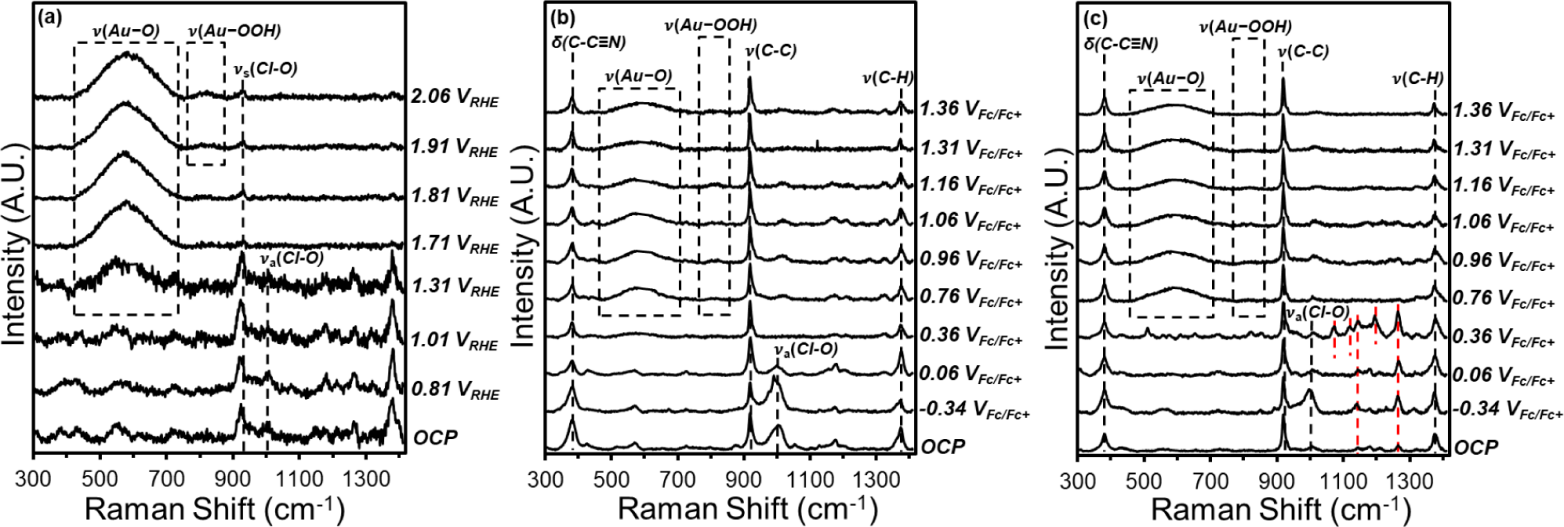
Potential-dependent steady-state Raman spectra of Au under flowing (a) aqueous electrolyte (0.1 M NaClO_4_, H_2_O) and aqueous–organic electrolytes (b) without (0.1 M TBAClO_4_, 10 M H_2_O, CH_3_CN) and (c) with C_6_H_12_ (0.1 M C_6_H_12_, 0.1 M TBAClO_4_, 10 M H_2_O, CH_3_CN). The red dashed lines in Figure 3c denote features that correspond to vibrational modes of liquid-phase C_6_H_12_.

*In situ* surface enhanced Raman spectroscopy (SERS) reveals species adsorbed on the anode and located in the nearby solution (e.g., in the electrochemical double layer) for different electrolyte compositions and as functions of potential. Figure 3 shows changes in Raman features that signify the oxidation of Au surfaces and the adsorption onto or concentration of species near the anode. In Figure 3a, a broad feature at 580 cm^–1^ appears at 1.31 V_RHE_ and intensifies as the potential increases to 2.06 V_RHE_. This feature corresponds to the ν(Au–O) of monatomic O* and Au oxides as described on electrochemically roughened Au in aqueous H_2_O_2_ (560 cm^–1^)^84^ and at anodic potentials in neutral,^72,85^ acidic,^54,65,66^ and alkaline^54,64^ electrolytes (550–590 cm^–1^). The ν(Au–O) features appear at the onset potential of Au surface oxidation and intensify in proportion to the total charge passed in aqueous solutions. As electrode potentials decrease toward more reducing potentials, the peaks for ν(Au–O) persist and only disappear following electrokinetic features that signify the reduction of Au oxide (Figure S26). Figure 3b,c shows that Au oxidation proceeds in a similar manner in aqueous–organic electrolytes. The 580 cm^–1^ feature appears after the onset potential of surface Au oxidation (0.65 V_Fc/Fc+_) and then intensifies with increasing potentials before becoming constant. This behavior occurs both in the absence (Figure 3b) and presence of C_6_H_12_ (Figure 3c). These *in situ* SERS results confirm the electrochemically induced, reversible reconstruction of the Au surface to Au oxide and agree with the conclusions drawn from the cyclic voltammetry measurements (Figure 1).

Raman scattering features indicate that other forms of reactive oxygen also bind to the anode. Figure 3a contains a feature at 820 cm^–1^ that appears at potentials greater than 1.8 V_RHE_ in neutral aqueous electrolyte solutions (0.1 M NaClO_4_, H_2_O) and at potentials greater than 1.6 V_RHE_ in alkaline electrolytes (0.1 M KOH, H_2_O) (Figure S27). These potentials correspond to the onset of the OER in the electrolytes. Literature reports attribute Raman scattering features near this position primarily to the ν(O–O) of OOH* species. Diaz-Morales et al. assigned a broad peak observed at 810 cm^–1^ under oxidizing conditions on Au in an acidic electrolyte (1 M HClO_4_, H_2_O) to the ν(O–O) of OOH*, based on DFT calculation on several Au facets.^65^ Similar features at 827 and 832 cm^–1^ were attributed to OOH* on Au in separate studies in alkaline electrolytes (0.1 M KOH, H_2_O).^64,66^ Notably, these features occur at greater frequencies than computed and experimentally observed modes of ν(Au–OH) (600–680 cm^–1^)^66,86^ and δ(Au–OH) (730–800 cm^–1^),^65,72,87^ assigned to OH*. Therefore, we attribute the feature at 820 cm^–1^ to the ν(O–O) mode of OOH*. The feature at 820 cm^–1^ appears at oxidizing potentials (> 0.76 V_Fc/Fc+_) in the aqueous–organic electrolytes with weaker intensity in comparison to the aqueous electrolyte, which signified lower coverages of OOH*.

Additional species from the electrolyte (CH_3_CN, ClO_4_^–^) specifically adsorb or remain near the Au anode at oxidizing potentials. Figure 3a possesses a sharp feature at 925 cm^–1^ evident in spectra collected at all potentials (OCP to 2.06 V_RHE_), which corresponds to the symmetric stretching of ClO_4_^–^ from the supporting electrolyte near the anode surface.^88,89^ A feature at 1005 cm^–1^ appears from 0.81 to 1.31 V_RHE_ in the aqueous electrolyte and −0.34 to 0.06 V_Fc/Fc+_ and −0.34 to 0.36 V_Fc/Fc+_ in the aqueous–organic electrolyte without and with C_6_H_12_, respectively. This feature correlates to the asymmetric stretching of ClO_4_^–^^77,90−92^ and likely decreases in intensity as potentials become more oxidizing because of solvent reorganization within the electrochemical double layer. Figures 3b,c, S29, and S30 contain a set of clear features (380 cm^–1^, 918 cm^–1^ 1375 cm^–1^, 2250 cm^–1^, 2950 and 3000 cm^–1^) that originate from internal vibrational modes of CH_3_CN (δ(C–C≡N), ν(C–C), ν(C–H), ν(C≡N), ν_s_(C–H), ν_a_(C–H)) near the electrode.^93,94^ Several features (1080 cm^–1^, 1115 cm^–1^, 1150 cm^–1^, 1210 cm^–1^, 1285 cm^–1^) that represent vibrational modes of C_6_H_12_ (ν(C–C), ν(C–C), ν(C–C), τ(C–H sp^3^), ρ(C–H sp^2^)) near the electrode appear at potentials less than 0.76 V_Fc/Fc+_.^95^ These features become less intense and no longer appear in the spectra at oxidizing potentials because of solvent reorganization. The sharpness of the CH_3_CN and C_6_H_12_ features indicates these species reside in the liquid phase 1–5 nm from the anode.^96^ A broad shoulder emerges at ∼2200 cm^–1^ when the electrode exceeds potentials of 1.31 V_Fc/Fc+_ (Figure S31b), which we attribute to the ν(C≡N) mode of CH_3_CN coordinated to Lewis acidic Au^n+^ surface sites.^97−99^ This feature does not appear in the presence of C_6_H_12_ (Figure S31b), which implies that C_6_H_12_ displaces CH_3_CN from the surface.

These spectroscopic observations confirm that in aqueous CH_3_CN solutions and at electrode potentials that oxidize C_6_H_12_ and evolve O_2_, the Au surface transforms into an Au oxide surface covered in O* and organic species reside upon or near the surface. Furthermore, *in situ* SERS provides direct evidence that electrochemical H_2_O activation forms pools of distinguishable oxygen species (O*, OOH*) in aqueous and aqueous–organic electrolytes. Although multiple forms of H_2_O-derived oxygen species populate the surface, the identity of the reactive oxygen species responsible for epoxidation of alkenes must be determined by analysis of kinetic measurements with molecularly detailed rate expressions.

### 3.3 Reaction Mechanism Insight from Epoxidation Rate Measurements

**Figure 4:**
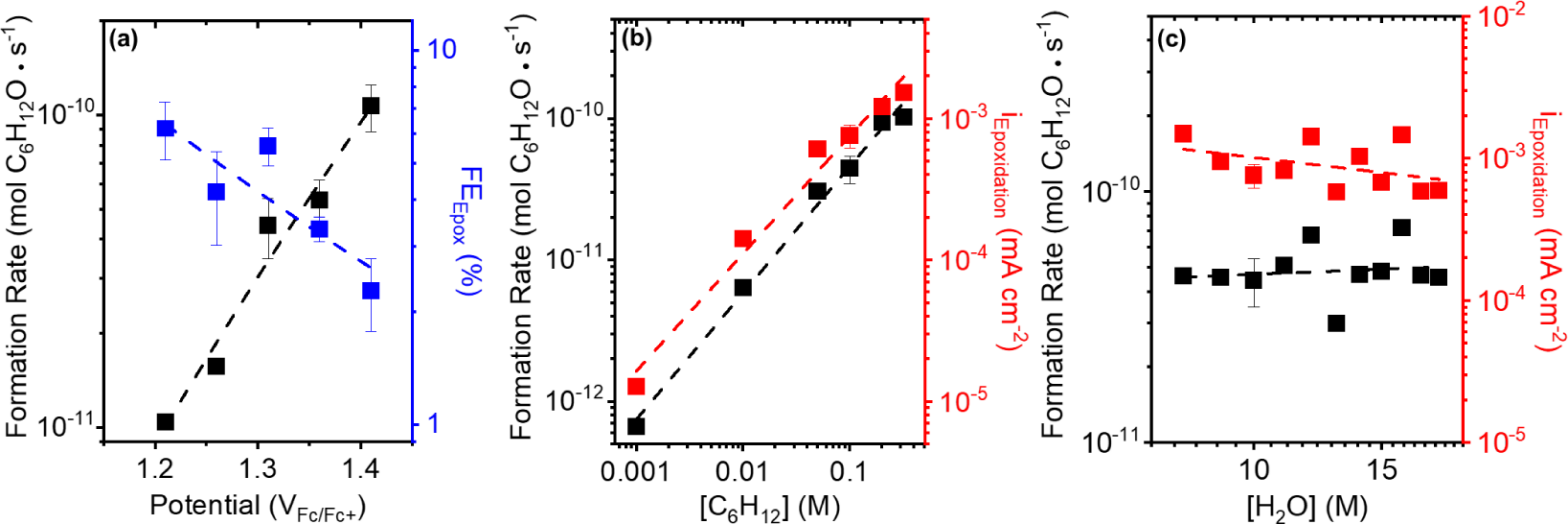
(a) C_6_H_12_O formation rates (black) and epoxidation Faradaic efficiencies (blue) as functions of potential (0.1 M C_6_H_12_, 0.1 M TBAClO_4_, 10 M H_2_O, CH_3_CN). C_6_H_12_O formation rates (black) and epoxidation partial current densities (red) as functions of (b) [C_6_H_12_] (1.31 V_Fc/Fc+_, 0.1 M TBAClO_4_, 10 M H_2_O, CH_3_CN) and (c) [H_2_O] (1.31 V_Fc/Fc+_, 0.1 M C_6_H_12_, 0.1 M TBAClO_4_, CH_3_CN).

Chronoamperometry measurements as a function of potential, [C_6_H_12_], and [H_2_O] demonstrate that Au catalyzes the epoxidation of C_6_H_12_ to C_6_H_12_O, and measured product formation rates and selectivities provide evidence for the mechanism of the epoxidation reaction. Figure 4a shows that C_6_H_12_O formation rates increase linearly, and epoxidation Faradaic efficiencies (FE_Epox_) decrease linearly with increasing potential. In addition, Figure 4b,c show that epoxide formation rates increase in proportion to [C_6_H_12_]  ∼ [C_6_H_12_]^0.9±0.2^, 0.01–0.32 M C_6_H_12_) and remain nearly constant with changes in [H_2_O] ( ∼ [H_2_O]^0.1±0.3^, 8–18 M H_2_O). These rate measurements suggest that Au-catalyzed alkene epoxidation involves kinetically relevant oxygen transfer to C_6_H_12_, and both epoxidation and OER occur by sequences of elementary steps that proceed on active sites saturated by H_2_O-derived surface oxygen species. Epoxidation measurements with H_2_^18^O coupled with GC–MS product analysis confirm that the oxygen in C_6_H_12_O comes from H_2_O (Section S21).

The chronoamperometry and cyclic voltammetry results provide evidence that C_6_H_12_O and O_2_ both form by reactions that involve a common pool of oxygen surface intermediates derived from H_2_O, before the pathways to these specific products diverge. We derive expressions for ratios of C_6_H_12_O formation rates to O_2_ formation rates  and epoxidation FE_Epox_ from analytical C_6_H_12_O and O_2_ formation rate expressions for five proposed mechanisms (Section S19), inspired in part by prior investigations.^35,42,43,45^ Each mechanism results in distinct predictions for the functional dependence of  and FE_Epox_ on [C_6_H_12_] and [H_2_O], which provide a basis for quantitative comparison to experimental results. Notably, use of  and FE_Epox_ does not require any assumptions about the dominant surface species on the anode. We consider four Eley–Rideal mechanisms where a solvent-phase C_6_H_12_ molecule reacts with either an O*, OH*, OOH*, or O_2_* species and a Langmuir–Hinshelwood mechanism where an adsorbed C_6_H_12_* species reacts with an O* species.

**Scheme 1:**
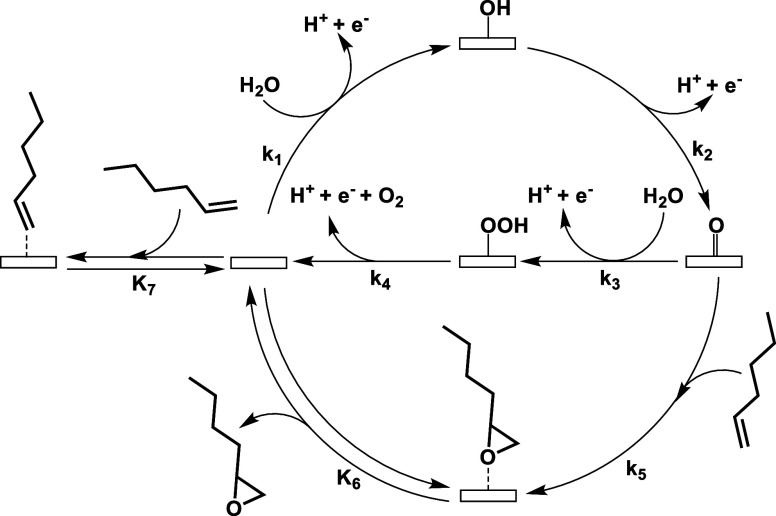
Proposed Catalytic Cycles for C_6_H_12_ Epoxidation with O* Species Derived from Electrochemical H_2_O Activation and O_2_ Evolution All adsorption steps and proton-electron transfer steps are currently assumed as reversible and irreversible, respectively. The hydrogen evolution reaction occurs at the cathode and consumes the H^+^ and e^–^ formed during C_6_H_12_ epoxidation and O_2_ evolution.

Scheme 1 depicts the single set of elementary steps consistent with experimental measurements (*vide infra)* of C_6_H_12_ and O_2_ formation rates, which involves direct reaction between a liquid-phase C_6_H_12_ molecule and a surface O* to form the epoxide via an Eley–Rideal pathway. Both products form in cycles that start with the proton–electron transfer from H_2_O to form an OH* species (step 1). The OH* undergoes subsequent proton–electron transfer to form O* (step 2). From here the mechanisms for O_2_ and C_6_H_12_O production diverge. To create O_2_, the O* species undergoes a nucleophilic attack with a solvent H_2_O molecule to form OOH* (step 3), which then dissociates to produce an O_2_ molecule (step 4).^65,66,100^ To form the epoxide, a solvent C_6_H_12_ molecule reacts with the O* species (step 5, a non-Faradaic process), which forms an epoxide intermediate (C_6_H_12_O*) that desorbs (step 6). In addition, C_6_H_12_ may adsorb reversibly (step 7). The oxidizing conditions on the anode lead to irreversible proton–electron transfer steps. Within the aqueous acetonitrile solution, H_2_O molecules transport protons that form by H_2_O oxidation away from the anode to the cathode, where H_2_ forms by the hydrogen evolution reaction (2H^+^ + 2e^–^ → H_2_). The other four catalytic cycles (Section S22) contain identical steps for the O_2_ evolution reaction but distinct processes and reactive species for the epoxidation of C_6_H_12_.

The rate expression for the steady-state formation of the epoxide product corresponds to the net rate of step 6 and step 5, in which the O* reacts to form C_6_H_12_O*:
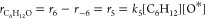
4where *k*_*i*_ and *k*_*–i*_ are the forward and reverse rate constants for step *i*. Application of the pseudo steady state hypothesis (PSSH) to the number of O* species and substitution of this expression into eq 4 yields
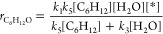
5where [*] is the number of unoccupied active sites.

An expression for [*] derives from the sum of all likely surface intermediates and recognition that the total number of active sites ([*L*]) remains constant:
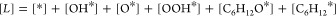
6where [x*] is the number of adsorbed species x*. The PSSH allows for the numbers of all adsorbates in eq 6 to be restated in terms of rate constants and liquid-phase reactant concentrations (further expanded upon in Section S22.1):
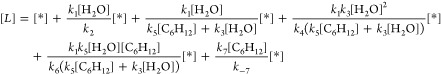
7

The combination of eqs 5 and 7 yields the complete expression for the epoxidation turnover rate:
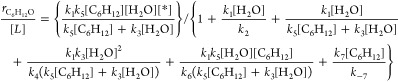
8

The form of eq 8 simplifies considerably in limits where the coverage of a given species far exceeds those of other reactive surface intermediates (*vide infra*). Analytical turnover rate expressions were derived for the other four mechanisms considered for epoxidation (Section S22).

Similar methods yield turnover rate expressions for the OER. The O_2_ formation rate equals the rate of step 4, in which the OOH* intermediate dissociates to form O_2_:
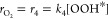
9

Application of the PSSH to [OOH*] and substitution of this expression into eq 9 gives
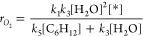
10

Combining eqs 7 and 10 yields an expression for the turnover rate for O_2_ evolution turnover rate:
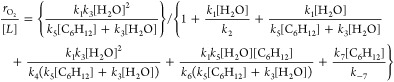
11

The ratio of C_6_H_12_O formation rates to O_2_ formation rates collapses to a simple form, because these reactions share a subset of steps that activate H_2_O and involve reactions of the common O* intermediate:
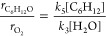
12

The FE_Epox_ expression relates the rate of epoxidation and O_2_ evolution to the number of electrons each process produces (two for epoxidation and four for OER):
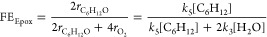
13

The same analysis was performed for the other four proposed epoxidation reaction mechanisms (Section S22).

**Figure 5:**
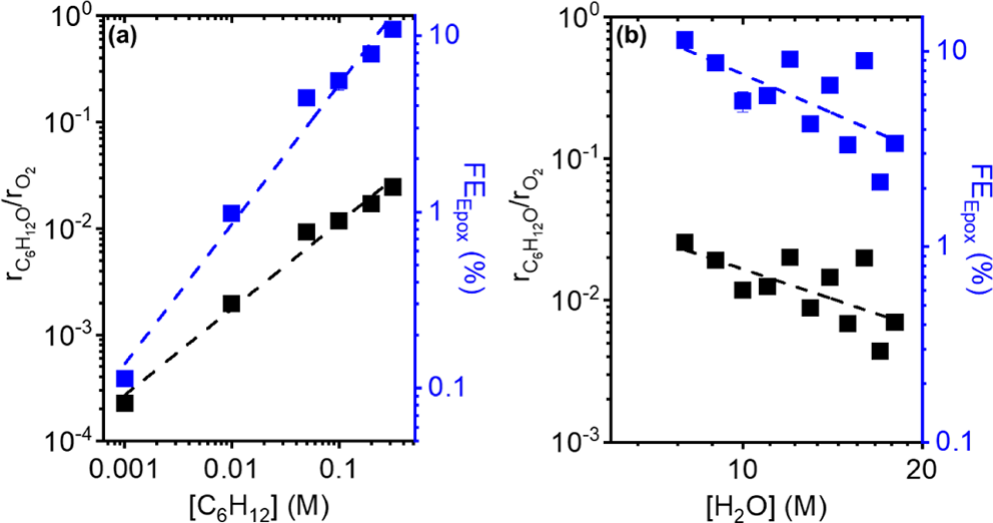
Ratios of C_6_H_12_O and O_2_ formation rates (black) and epoxidation Faradaic efficiencies (blue) as functions of (a) [C_6_H_12_] (1.31 V_Fc/Fc+_, 0.1 M TBAClO_4_, 10 M H_2_O, CH_3_CN) and (b) [H_2_O] (1.31 V_Fc/Fc+_, 0.1 M C_6_H_12_, 0.1 M TBAClO_4_, CH_3_CN).

Table 1 summarizes the analytical expressions for the  and FE_Epox_ derived for each of the five proposed mechanisms. Expressions for  carry a linear dependence on [C_6_H_12_] for all reactions with the sole exception of the Eley–Rideal mechanism with O_2_*, which remains independent of [C_6_H_12_]. In addition, the functional form of the equations for FE_Epox_ predict values that depend on [H_2_O] and [C_6_H_12_] for all cases except for epoxidation via O_2_*, which appears to be independent of reactant concentrations. Notably, O_2_ formation rates far exceed C_6_H_12_O formation rates (Figures 4a and 5). Consequently, the denominator of FE_Epox_ expressions remain nearly constant, and values of FE_Epox_ will increase in proportion to [C_6_H_12_] for epoxidation by reaction with O*, OH*, or OOH*. Forms for the  and FE_Epox_ from the mechanisms that involve direct reaction between O* and C_6_H_12_ species (both Eley–Rideal and Langmuir–Hinshelwood) possess an inverse dependence on [H_2_O]. In contrast,  and FE_Epox_ for the remaining mechanisms (OH*, OOH*, and O_2_*) do not explicitly depend on [H_2_O].

**Table 1:** Derived Rate Ratios and FE_Epox_ for Proposed Mechanisms^a^

Mechanism for C_6_H_12_ Reaction		FE_Epox_
Eley–Rideal with O*		
Eley–Rideal with OH*		
Eley–Rideal with OOH*		
Eley–Rideal with O_2_*		
Langmuir–Hinshelwood with O*		

a β represents a group of rate constants and reactant concentrations as follows: 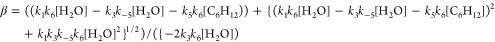

Figure 5a shows that measured values for  and FE_Epox_ depend linearly on [C_6_H_12_]. The near linear dependence of  (0.8 ± 0.2) and FE_Epox_ (0.8 ± 0.2) on [C_6_H_12_] does not agree with expectations for the mechanism that involves O_2_* (Table 1), but agrees with predictions for reactions via O*, OH*, or OOH*. Figure 5b shows that  and FE_Epox_ depend inversely on [H_2_O] (−1.4 ± 0.4 and −1.3 ± 0.4, respectively). These observations exclude mechanisms in which C_6_H_12_ reacts with either OH*, OOH*, or O_2_* (Table 1), which predict these quantities do not change with [H_2_O]. Only mechanisms that involve the reaction between C_6_H_12_ and O* species (Eley–Ridel or Langmuir–Hinshelwood) give analytical expressions for rate ratios and Faradaic efficiencies consistent with the experiments. Furthermore, the  follows a linear function when considered a function of  (Figure S34). Only Eley–Rideal and Langmuir–Hinshelwood mechanisms with a reactive O* species predict a linear dependence of these two values on . We note that the gradient of  dependence on  differs between the measurements with varied [C_6_H_12_] and [H_2_O]. This difference likely stems from interactions within the electrochemical double layer, because C_6_H_12_ and H_2_O will stabilize surface species and transition states (epoxidation, O_2_ evolution) in different manners. The comparisons between steady-state rates (Figures 4 and 5) and analytical rate expressions along with cyclic voltammetry experiments (Figure 1) provide strong evidence that the C_6_H_12_ epoxidation mechanism proceeds via the non-Faradaic reaction of a solvent or surface-bound C_6_H_12_ species with a reactive intermediate O* species.

The use of aqueous–organic electrolytes in the kinetic studies requires consideration of the nonideal behavior of C_6_H_12_ and H_2_O in solution.^79^ Kinetic and equilibrium behavior in these systems depend on the thermodynamic activities of C_6_H_12_ and H_2_O, and consequently, we recast measurements of  and FE_Epox_ to show the dependence of these terms explicitly (Figure S35): both quantities increase linearly with C_6_H_12_ activity and show approximately an inverse fourth-order dependence on H_2_O activity. These observations most closely agree with predictions for mechanisms that involve O* (and once again exclude other proposals), however, the high order dependence on the activity of H_2_O indicates that the analytical expressions do not capture all relevant interactions. This discrepancy seems likely to reflect the nonideal interactions between the epoxidation transition state and the surrounding species contained in the electrochemical double layer, which possesses a composition dependent upon, yet far different, from the solvating environment for reagents in the bulk fluid.^101^

The epoxidation of *cis-*stilbene and analysis of the product distribution provides further evidence that the C_6_H_12_ epoxidation mechanism involves a reaction with O* and excludes other forms of reactive oxygen. Figure S36 shows that *cis-*stilbene epoxidation on Au results in nearly equal formation rates of both *cis*-stilbene oxide and *trans-*stilbene oxide, which indicates that epoxidation occurs through a stepwise mechanism that allows for rotation about the C–C bond before closing the oxirane ring. These observation agree with expectations for epoxidations with O* species, which can proceed through either stepwise^102−105^ or concerted^106−108^ mechanisms, or with reactions through O_2_* that predominantly occur by sequential formation of C–O bonds and yield near equilibrium distributions of isomers.^109−111^ In contrast, *cis*-stilbene epoxidation with OOH* occurs via a concerted pathway that retains the stereochemistry of the reactant and yields predominantly *cis-*stilbene oxide (>90%) with trace amounts of *trans*-stilbene oxide (<5%).^112−114^ This further excludes the possibility that the OOH* species participates in the epoxidation mechanism. As discussed earlier, the dependencies of  and FE_Epox_ on [H_2_O] eliminate the possibility of an O_2_* reactive species. Thus, the combination of the rate ratio and Faradaic efficiency dependency analysis and *cis*-stilbene epoxidation product distribution support that alkenes react with O* on Au.

Establishing that the epoxidation mechanism on Au proceeds through the reaction of a solvent-phase or surface-bound alkene molecule with an O* species, the full epoxidation rate expression can be simplified. O* species cover the Au surface at all potentials greater than the onset of Au surface oxidation (Section 3.2). The rate expression for the Eley–Rideal mechanism (eq 8) collapses with the assumption that the O* species acts as the most abundant surface intermediate:
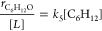
14and similarly, the rate expression for the Langmuir–Hinshelwood mechanism (eq S116) reduces to
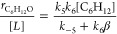
15

Both eqs 14 and 15 match the rate behavior observed (Figure 4) in which the epoxidation rate shows a first-order dependence on [C_6_H_12_] and zero-order dependence on [H_2_O], which supports the assumption of O* dominating at active sites. Furthermore, the measured Tafel slopes for epoxidation (273 ± 54 mV decade^–1^) and OER (184 ± 19 mV decade^–1^) qualitatively align with the predicted Tafel slopes (∞ and 120 mV decade^–1^, respectively) for an O* covered surface (Figure S37 and Section S25). The discrepancy between the measured and predicted Tafel slopes may result from deviation of the assumed charge transfer coefficient value of one-half. Furthermore, the epoxidation rate depends indirectly on potential, which leads to a finite Tafel slope, because the numbers of reactive O* species at steady state can increase with the applied potential. Although O* appears to be more abundant than all other surface intermediates, the fractional coverage of this species increases toward a value of unity when more positive potentials lead to greater rate constants for H_2_O activation relative to C_6_H_12_ epoxidation (Section S25). The high value for the epoxidation Tafel slope provides further evidence that the epoxidation mechanism involves both Faradaic and non-Faradaic steps.

Broadly, these findings establish the mechanism of and surface structure during epoxidation on Au electrodes. *In situ* Raman spectroscopy confirms the presence of O* on Au under reaction conditions. The derived analytical rate expressions and rate ratios, *cis*-stilbene epoxidation products, and electrokinetics exclude the possibility that C_6_H_12_ reacts with OH*, OOH*, or OO* and provides evidence that C_6_H_12_ combines with O* to form C_6_H_12_O. These conclusions validate previous proposals in literature.^29,43,44,46^ The combination of kinetic and spectroscopic techniques demonstrates that on an oxidized Au surface in an aqueous–organic electrolyte C_6_H_12_ epoxidation and OER share a common pathway to a reactive O* species before diverging to form C_6_H_12_O or O_2_ respectively.

## 4 Conclusions

The suite of kinetic and spectroscopic measurements outlined here provides compelling evidence that C_6_H_12_O forms by a non-Faradaic reaction with surface O* species, derived from electrochemical activation of H_2_O on Au anodes. Cyclic voltammograms obtained for a range of [C_6_H_12_] demonstrate that the Au surface must electrochemically activate H_2_O for epoxidation to proceed. Furthermore, C_6_H_12_ epoxidation inhibits rates for the O_2_ evolution reaction, because both reactions compete for a common pool of reactive surface oxygen species. *In situ* Raman spectroscopy measurements confirm the presence of O* and OOH* on the Au surface at oxidizing conditions. The C_6_H_12_O formation rates display a first-order dependence on [C_6_H_12_] and zero-order dependence on [H_2_O], consistent with spectroscopic evidence that the Au surface contains high coverages of H_2_O-derived species and no appreciable coverages of C_6_H_12_-derived species. The high epoxidation Tafel slope indicates that the epoxidation mechanism consists of not only Faradaic, but also non-Faradaic steps. Agreement of experimental  and FE_Epox_ reagent dependencies with analytical expressions, *cis*-stilbene epoxidation product distribution, and matching predicted and experimental rate reagent dependencies and Tafel slopes assuming an O* covered surface provide evidence for an epoxidation mechanism that involves the addition of O*, and not OH*, OOH*, or O_2_*, to the alkene reactant. Knowledge of the identity of the reactive oxygen species participating in the epoxidation mechanism on Au anodes provides an opportunity to improve epoxidation rate and selectivity by stabilizing and promoting the formation of the reactive O* species, while minimizing the Faradaic formation of the OOH* species necessary for O_2_ evolution. In addition, the dependence of and FE_Epox_ on potential, [C_6_H_12_], and [H_2_O] demonstrate the promotion of epoxidation selectivity through operation at lower overpotentials, higher [C_6_H_12_], and lower [H_2_O]. Clearly, the combination of rate analysis and *in situ* spectroscopy allows for the elucidation of the reaction mechanism of reactions on electrodes and provides an opportunity to modulate selectivity toward desired products through catalyst and system design.

## Supporting Information Available

Kinetic measurements system, cleaning cyclic voltammetry, electrochemical surface area measurements, cyclic voltammetry reporting, reference electrode potential conversion, GC sensitivity factors, GC-FID chromatograms, epoxidation formation rate and Faradaic efficiency calculation, external mass transfer test, *in situ* Raman system, Raman spectra processing, reagent thermodynamic activities, supplemental cyclic voltammetry, ICP-OES, UV–vis spectroscopy, energy dispersive X-ray fluorescence spectroscopy, supplemental *in situ* Raman spectroscopy, Raman spectra peak assignments, GC–MS analysis, rate expression derivation, rate ratio and epoxidation Faradaic efficiency dependence on reagent thermodynamic activities, *cis*-stilbene epoxidation, Tafel slope analysis (PDF)
