# Supplementary material for: Alkene Epoxidation and Oxygen Evolution Reactions Compete for Reactive Surface Oxygen Atoms on Gold Anodes
Source: J Am Chem Soc. 2024 Dec 11;147(2):1482–96. doi: 10.1021/jacs.4c08948 (PMC11744761; doi:10.1021/jacs.4c08948)
Supplement: Supplementary file 1 — ja4c08948_si_001.pdf [file ja4c08948_si_001.pdf]

Supplementary Information

Alkene Epoxidation and Oxygen Evolution Reactions Compete for Reactive Surface Oxygen Atoms on Gold Anodes

Richa Ghosh[1], Geoffrey M. Hopping[1], Jordan W. Lu[1], Drew W. Hollyfield[2], David W. Flaherty[1*]

[1]School of Chemical and Biomolecular Engineering, Georgia Institute of Technology, Atlanta, Georgia 30332 (USA)

[2] Department of Chemical and Biomolecular Engineering, University of Illinois Urbana-Champaign, Urbana, Illinois 61801 (USA)

[*]Corresponding Author
Phone: (404)-894-5922
Email: dflaherty3@gatech.edu

S1. System for Kinetic Measurements

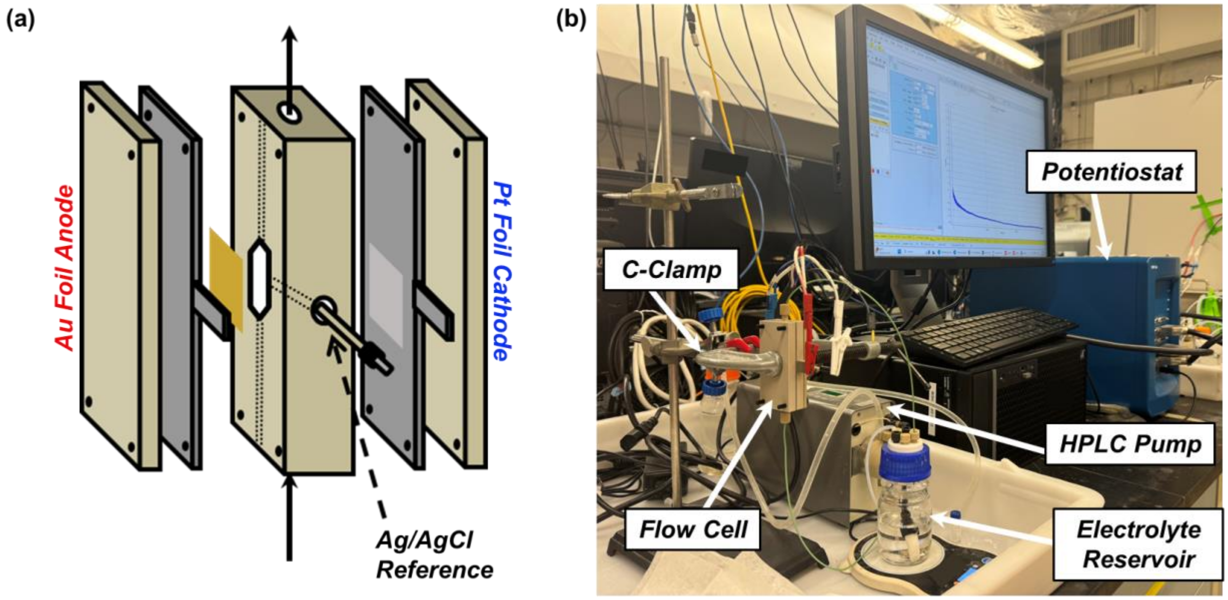

Figure S1. (a) Schematic of electrochemical flow cell. (b) Image of system used for cyclic voltammetry and epoxidation rate measurement experiments.

Figure S1a shows a schematic of the electrochemical flow cell used for all cyclic voltammetry and epoxidation rate measurements. A polycrystalline Au foil (Goodfellow, 99.999%), polycrystalline Pt foil (Thermo Scientific, 99.99%), and leak-free silver-silver chloride electrode (Ag/AgCl, Innovative Instruments, 3.4 M KCl leak-free 1.6 mm diameter) were used as the anode, cathode, and pseudo-reference electrode respectively. A polyetheretherketone (PEEK) plate separated the anode and cathode and contained a trapezoidal flow path with a geometric area of one cm^2. The Ag/AgCl reference electrode was placed so that it did not obstruct and was perpendicular to the electrolyte flow. Stainless-steel plates with extended tabs were used as current collectors for the anode and cathode. Two PEEK plates were the outermost layers of the flow cell to provide electrical insulation. Plastic screws (Nylon, McMaster-Carr) were used to assemble the cell.

Figure S1b shows an image of the system used for cyclic voltammetry and epoxidation rate measurements in a recirculating flow configuration. The potentiostat (Biologic, VSP-3e) was connected to the reference electrode and anode and cathode current collector plates using alligator clips. A C-clamp was used to compress the flow cell to prevent electrolyte leaking from the cell. The electrolyte (55-75 cm^3, 0-0.32 M C_6H_{12}, 0-18 M H_2O, 0.1 M $TBAClO_4$, CH_3CN) was stirred (400 rpm) in the electrolyte reservoir, which was a sealed, media bottle (Pyrex, 100 cm^3) with a cap containing threaded ports. The electrolyte was pumped (10 cm^3 min^{-1}) through the electrochemical flow cell using a high-performance liquid chromatography pump (Chrom Tech, HPLC, M1) with interior PEEK lining. The HPLC pump drew electrolyte from the electrolyte reservoir through an inlet solvent filter connected to ethylene tetrafluoroethylene (ETFE) tubing. The electrolyte was pumped out of the HPLC pump and into the inlet of the electrochemical flow cell through PEEK tubing. The flow cell was placed so the electrolyte flow would be perpendicular

to the ground to prevent accumulation of bubbles on the electrodes. In the recirculating flow configuration, the electrolyte exited the outlet of the electrochemical flow cell and entered the electrolyte media bottle through PEEK tubing. In the single-pass flow configuration, PEEK tubing connected to the outlet of the flow cell was placed in a waste container. During epoxidation rate measurements, the electrolyte was sampled from the reservoir using a syringe and needle through one of the threaded ports on the reservoir cap. The entire system operates at ambient temperature and pressure.

S2. Cleaning of Au Surface with Cyclic Voltammetry

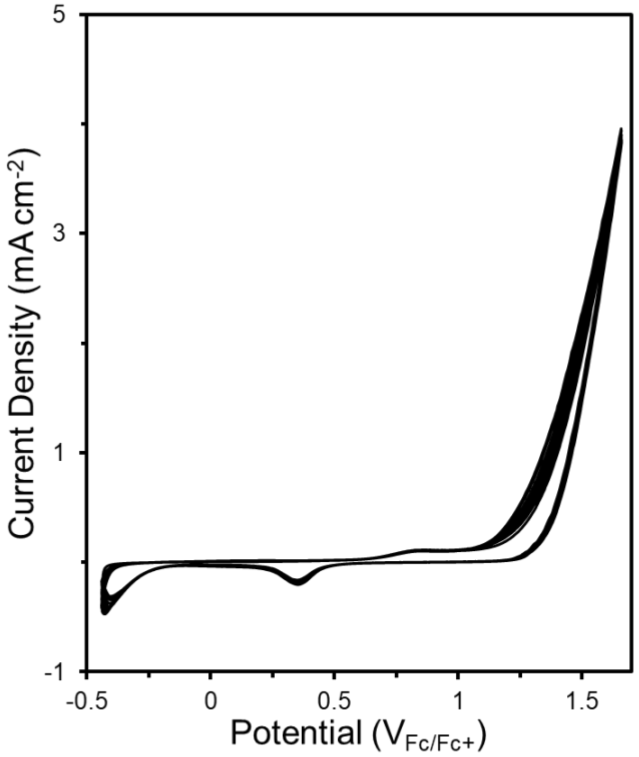

Figure S2 *In situ* cleaning of Au foil using cyclic voltammetry (20 cycles, 50 mV s^{-1}) in a cleaning electrolyte solution (0.1 M $TBAClO_4$, 10 M H_2O, CH_3CN).

Cyclic voltammetry (20 cycles, -0.44 to 1.66 $V_{Fc/Fc+}$, 50 mV s^{-1}) with a cleaning electrolyte solution (0.1 M $TBAClO_4$, 10 M H_2O in CH_3CN) was performed prior to any cyclic voltammetry or epoxidation rate measurements to prepare the electrode surface and remove any organic surface contaminants. **Figure S2** shows an overlay of the 20 cyclic voltammograms. The oxidation feature (0.65 to 0.94 $V_{Fc/Fc+}$) corresponds to Au surface oxidation and the two reduction features correspond to surface $Au_2O_{<3}$ reduction to $Au(OH)_3$ (0.2 to 0.6 $V_{Fc/Fc+}$) and $Au(OH)_3$ reduction to metallic Au (-0.44 to -0.05 $V_{Fc/Fc+}$). The O_2 evolution reaction (OER) occurs at potentials greater than 0.94 $V_{Fc/Fc+}$. Cycling through oxidizing and reducing potentials under flow allows for removal of organic contaminants on the surface through desorption or reaction. Furthermore, cycling the potential until the voltammograms converge allows for consistent surface reconstruction. The cyclic voltammograms converged after the 10th cycle, evident through minimal changes in the Au oxidation and reduction features, indicating that the surface no longer had any adsorbed contaminants.

S3. Electrochemical Surface Area Measurements

The electrochemical surface area (ECSA) was determined through analysis of the double-layer capacitance.[1] Cyclic voltammetry scans from ±50 mV of the open circuit potential (OCP), determined by the potentiostat, were recorded at scan rates of 5, 10, 20, 50, 80, 100 mV s^{-1}.

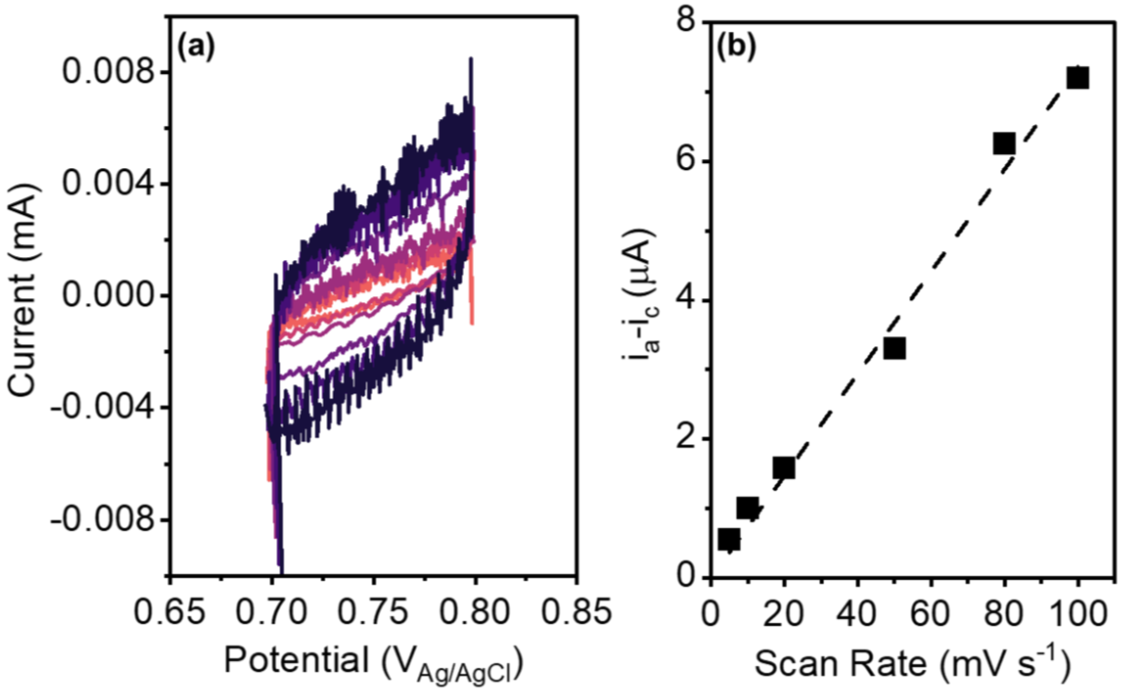

Figure S3. (a) Representative cyclic voltammetry traces (5, 10, 20, 50, 80, 100 mV s^{-1}) measured to determine the electrochemical surface area and (b) linear fit of the current at OCP versus scan rate. The data here is shown for the polycrystalline Au anode (0.1 M C_6H_{12}, 0.1 M $TBAClO_4$, 10 M H_2O, CH_3CN).

The electrode double-layer capacitance (C_{DL}, μF) was calculated by plotting the difference between the electrode currents during anodic (i_a) and cathodic (i_c) scans near the OCP as a function of the scan rate (**Figure S3**) and dividing the resulting slope by two (**Equation S1**). The specific capacitance (C_S) of a pristine polycrystalline Au foil equals 11.95 μF cm^2, which was determined by dividing the electrode double-layer capacitance by the geometric area (1 cm^2), and the ECSA following **Equation S2**. The average ECSA for the kinetic measurements reported in this study is 3.1 ± 0.3 cm^2.

$$C_{Dl} = \frac{1}{2}\frac{\partial(i_a - i_c)}{\partial(scan\ rate)} \quad \text{(S1)}$$

$$ECSA = \frac{C_{Dl}}{C_S} \quad \text{(S2)}$$

S4. Reporting Cyclic Voltammograms

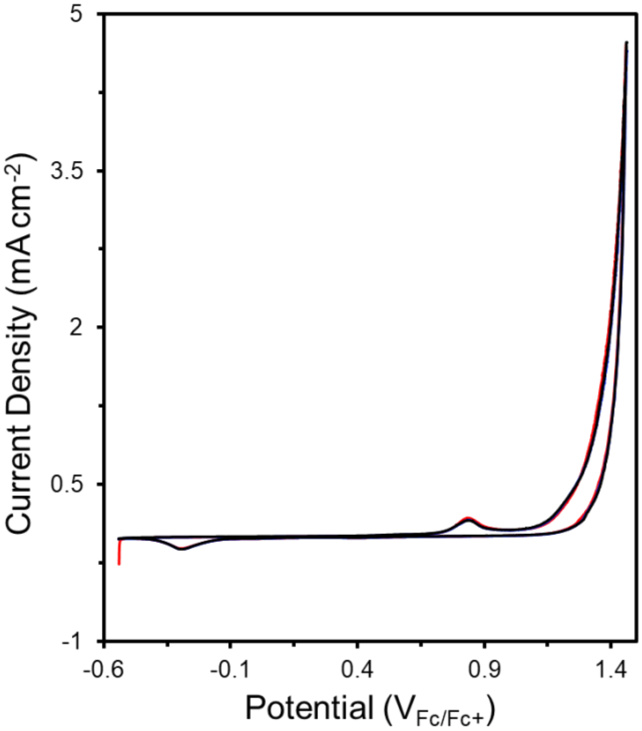

Figure S4. Representative cyclic voltammetry traces (0.1 M C_6H_{12}, 0.1 M $TBAClO_4$, 10 M H_2O, CH_3CN, 10 mV s^{-1}). Cyclic voltammetry was performed in triplicates (first, second, third cycle)

Three cyclic voltammograms were collected per cyclic voltammetry measurement and before chronoamperometry measurements. For all measurements, the second and third cyclic voltammograms converged, which confirms the stability of the Au surface. Reported cyclic voltammograms in this work represent the third cycle.

S5. Conversion of Reference Electrode Potentials

The leak-free Ag/AgCl pseudo-reference electrode $\left(E_{Ag/AgCl}\right)$ was calibrated against the ferrocene/ferrocenium (Fc/Fc^{+}) redox potential (**Equation S3**) in order to report potentials $\left(E_{Fc/Fc^{+}}\right)$ in the aqueous-organic electrolyte. Ferrocene (0.005 M Fc) was added to electrolyte solutions (0.1 M $TBAClO_4$, CH_3CN) with varying [C_6H_{12}] and [H_2O]. Cyclic voltammetry was performed in the flow cell with a carbon paper working electrode (Fuel Cell Earth, LLC, Toray, TGP-H-060) and Pt foil counter electrode. The potential sweep (50 mV s^{-1}) started at open circuit potential (OCP), then went to 0 $V_{Ag/AgCl}$, followed by three cycles from 0 to 0.8 $V_{Ag/AgCl}$, and ended after sweeping back to OCP. The redox potential $\left(E_{\frac{1}{2},Fc/Fc^{+}}\right)$ was calculated by averaging the resulting oxidative and reductive peaks for the second scan (**Figure S5**). Note that the calibration values for electrolytes with varying [C_6H_{12}] and [H_2O] was calculated from the linear fits of **Figures S6a** and **S6b** respectively.

$$E_{Fc/Fc^{+}} = E_{Ag/AgCl} - E_{\frac{1}{2},Fc/Fc^{+}} \quad \text{(S3)}$$

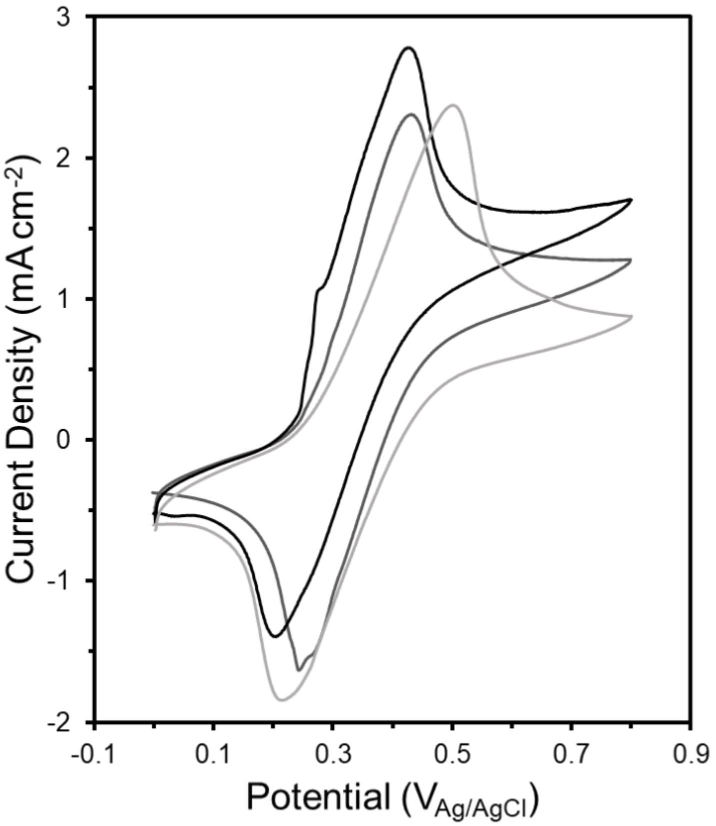

Figure S5. Representative cyclic voltammograms for the Fc/Fc^{+} redox calibrations across a range of H_2O concentrations that encompass those used for rate measurements including 0.5 M H_2O (light gray), 10 M H_2O (dark gray), and 18 M H_2O (black) in aqueous CH_3CN electrolyte (0.1 M C_6H_{12}, 0.1 M $TBAClO_4$, CH_3CN).

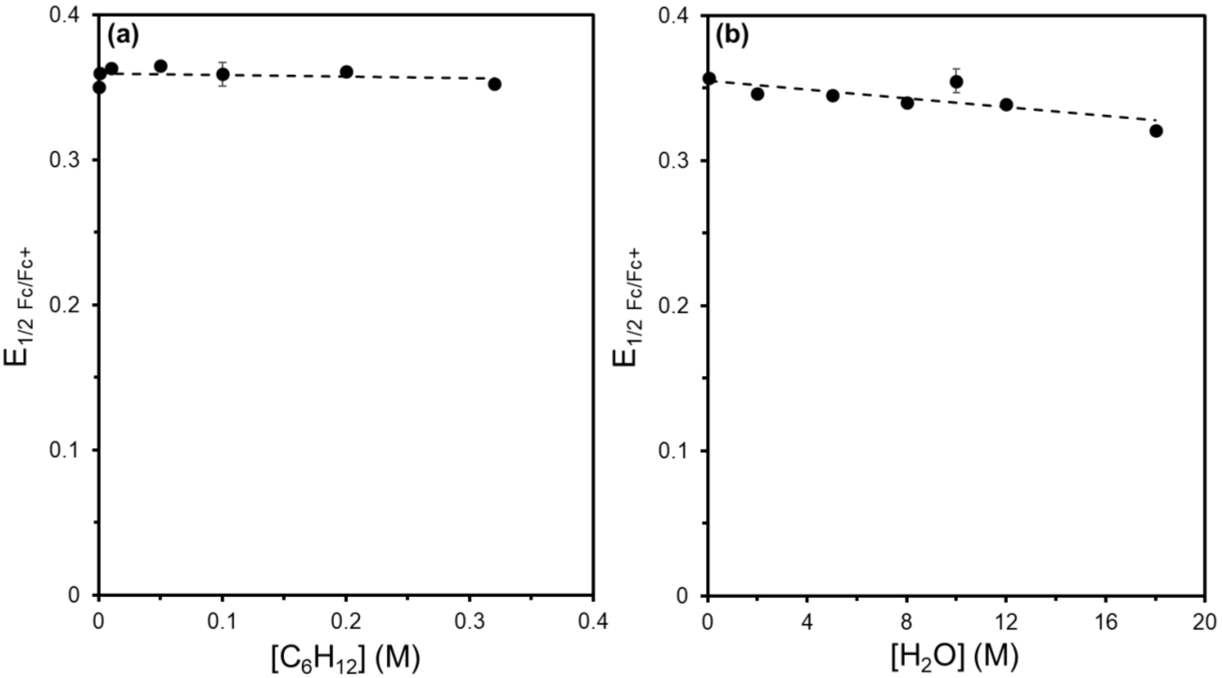

Figure S6. Fc/Fc$^+$ redox potential $\left(E_{\frac{1}{2},Fc/Fc^+}\right)$ as functions of (a) [C_6H_{12}] (0.005 M Fc, 0.1 M TBAClO$_4$, 10 M H_2O, CH_3CN) and (b) [H_2O] (0.005 M Fc, 0.1 M C_6H_{12}, 0.1 M TBAClO$_4$, CH_3CN).

Potentials in aqueous electrolytes are reported versus the reversible hydrogen electrode (RHE). Potentials collected using the Ag/AgCl pseudo-reference electrode were converted to RHE using the following equation:

$$E_{RHE} = E_{Ag/AgCl} + E^{o}_{Ag/AgCl} + 0.059pH \tag{S4}$$

Where E_{RHE} is the converted potential versus RHE, $E_{Ag/AgCl}$ is the experimentally measured potential against Ag/AgCl reference, and $E^{o}_{Ag/AgCl}$ is the standard reduction potential of Ag/AgCl versus the standard hydrogen electrode (0.1976 at 25ºC for 3.4 M KCl).[2]

S6. Calculation of C_6H_{12} and $C_6H_{12}O$ Sensitivity Factors and GC Method Uncertainty

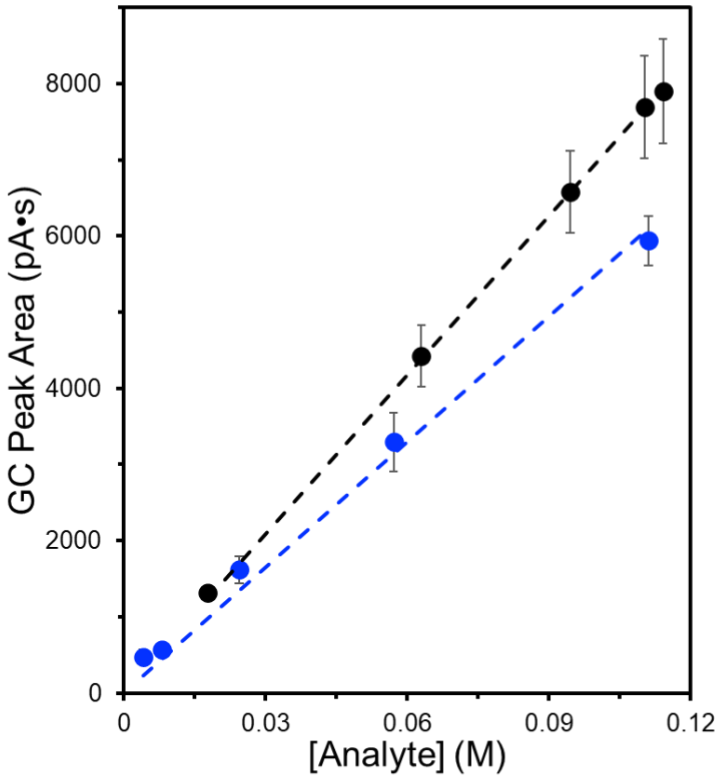

Figure S7. Integrated GC peak areas as functions of concentration for C_6H_{12} (black) and $C_6H_{12}O$ (blue).

Figure S7 shows areas of peaks corresponding to C_6H_{12} and $C_6H_{12}O$ obtained by gas chromatography of samples prepared from solutions with known concentrations of C_6H_{12} and $C_6H_{12}O$. Liquid-liquid extraction of electrolytes (C_6H_{12} and $C_6H_{12}O$, 0.1 M $TBAClO_4$, 10 M H_2O, CH_3CN) with decane was used to produce organic samples injected to the gas chromatograph. **Table S1** reports each analyte's sensitivity factor (S_i), which equals the slope of linear fits to the data in F**igure S7**.

Table S1. Sensitivity factors determined from calibration curves in **Figure S7**.

Analyte	S_i (pA•s M^{-1})
C_6H_{12}	69600
$C_6H_{12}O$	54900

The measured sensitivity factors with integrated peak areas were used to calculate the concentration of $C_6H_{12}O$ ($[C_6H_{12}O]$) using the following equation:

$$[C_6H_{12}O] = \frac{GC\ Peak\ Area}{S_{C_6H_{12}O}} \quad \text{(S5)}$$

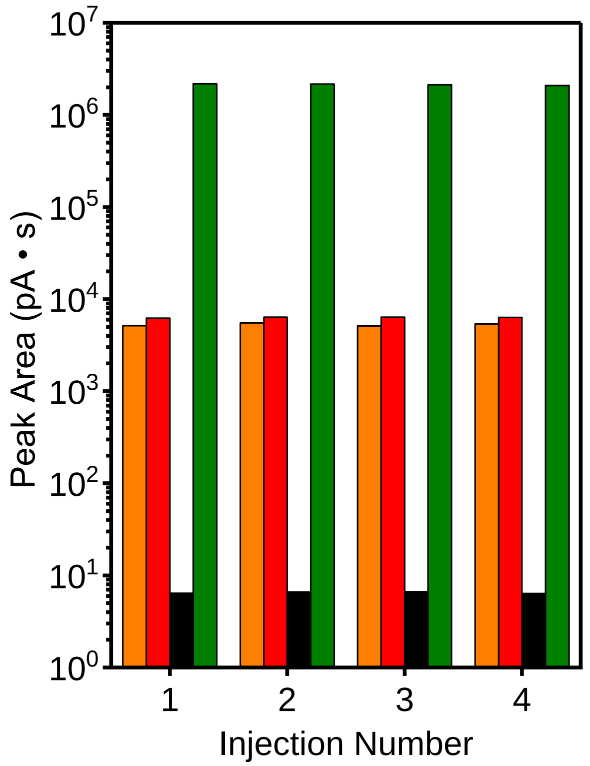

Figure S8. Average integrated GC peak areas of CH_3CN (orange), C_6H_{12} (red), $C_6H_{12}O$ (black), and decane (green) from repeated GC analysis (5 injections) of a representative electrolyte sample (0.1 M C_6H_{12}, 0.00011 M $C_6H_{12}O$, 0.1 M $TBAClO_4$, 10 M H_2O, CH_3CN).

Liquid-liquid extraction of a representative electrolyte (0.1 M C_6H_{12}, 0.00011 M $C_6H_{12}O$, 0.1 M $TBAClO_4$, 10 M H_2O, CH_3CN) with decane was performed, and a portion of the decane phase (1 cm^3) was pipetted into a chromatography autosampler vial. GC analysis was then performed on the same sample vial five times to determine the uncertainty of the GC analysis method. Integration of the peaks for CH_3CN, C_6H_{12}, $C_6H_{12}O$, and decane in the five sequential injections reveal that the GC method has an uncertainty of ~5% (**Figure S8**).

S7. GC Chromatograms of Electrolyte During Chronoamperometry

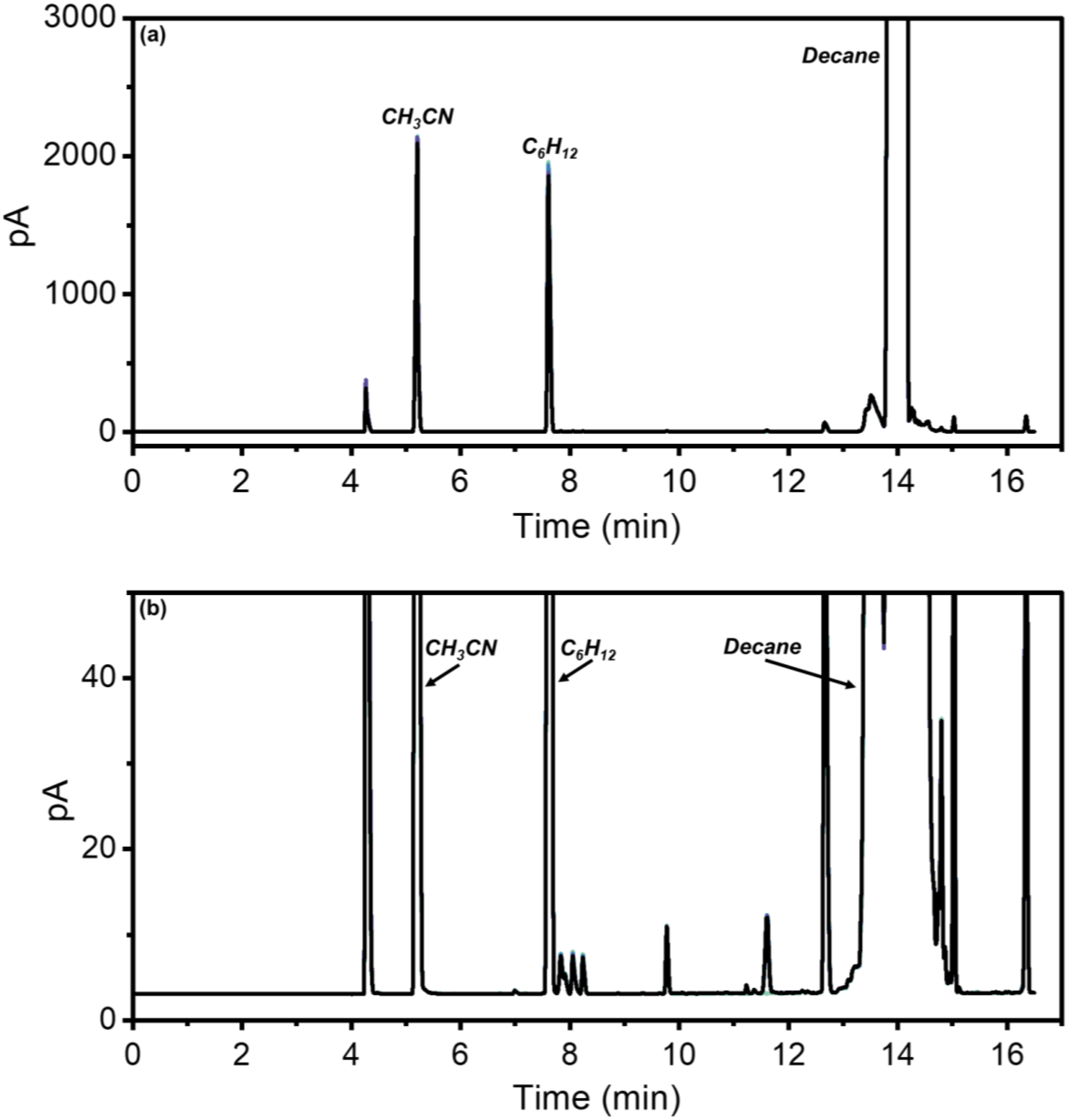

Figure S9. Representative raw GC chromatograms scaled from (a) 0 to 17 minutes and 0 to 3000 pA and (b) 0 to 17 minutes and 0 to 40 pA of an electrolyte sampled at 0, 6, 12, 18, and 22 hours during a chronoamperometry measurement (1.31 $V_{Fc/Fc+}$, 0.1 M C_6H_{12}, 0.1 M $TBAClO_4$, 10 M H_2O, CH_3CN). The chromatograms at different times overlay nearly perfectly with one another at the scale shown.

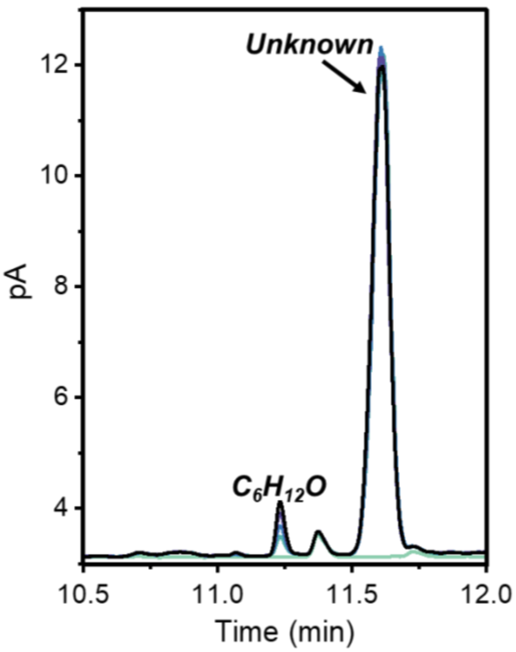

Figure S10. Representative raw GC chromatogram of an electrolyte sampled at 0, 6, 12, 18, and 22 hours scaled to show peaks corresponding to $C_6H_{12}O$ and an unknown product that appears during the chronoamperometry measurement (1.31 $V_{Fc/Fc+}$, 0.1 M C_6H_{12}, 0.1 M $TBAClO_4$, 10 M H_2O, CH_3CN).

Figure S9 shows representative raw GC chromatograms of an electrolyte sampled over the course of a 22-hour chronoamperometry measurement (1.31 $V_{Fc/Fc+}$, 0.1 M C_6H_{12}, 0.1 M $TBAClO_4$, 10 M H_2O, CH_3CN). CH_3CN, C_6H_{12}, and decane elute out at 5.2, 7.6, and 14.1 minutes respectively (**Table S2**). Approximately 60 other peaks appear in the chromatogram before electrolysis. Their areas do not change over the course of electrolysis. The identities of these peaks remain unknown. These peaks may come from unreactive impurities in the decane, CH_3CN, or C_6H_{12}. Only two peaks at 11.2 and 11.6 minutes increase with time (**Figure S10**). The peak at 11.2 minutes corresponds to $C_6H_{12}O$ and increases linearly with time, as shown in **Figure S11a**. The identity of the peak at 11.6 minutes remains unknown. This peak appears once the chronoamperometry measurement begins (within an hour) but does not grow with time (**Figure S11a**). We have ruled out many possible products that may form through side reactions of C_6H_{12} or further oxidation of $C_6H_{12}O$ because these species appear at different retention times than the unknown peak, as shown in **Table S2**. The unknown peak may arise from an impurity in C_6H_{12} that undergoes a rapid, equilibrium-limited reaction. As the peak does not change over the course of the chronoamperometry measurement and the carbon balance closes within 95-101% (**Figure S11b**), we do not believe it reflects a product of C_6H_{12} oxidation.

The carbon balance is calculated with the following equation:

$$Carbon\ Balance = \frac{[C_6H_{12}O]+[C_6H_{12}]}{[C_6H_{12}]_o} \tag{S6}$$

Where *[$C_6H_{12}O$]* and *[C_6H_{12}]* are the concentrations of $C_6H_{12}O$ and C_6H_{12} at a given time point and *[C_6H_{12}]$_o$* is the initial [C_6H_{12}].

Table S2. Measured GC retention times for species within the electrolyte (CH_3CN, C_6H_{12}, decane), epoxidation products, unknown peak, and plausible side and further oxidation products.

GC Retention Time (min)	Species
5.2	CH_3CN
7.6	C_6H_{12}
9.3	Pentanal
10.9	2-Hexanone
11.0	Hexanal
11.1	2-hexanol
11.2	1,2-Epoxyhexane
**11.6**	**Unknown**
12.0	1-Hexanol
14.1	Decane
14.3	1,2-Hexanediol

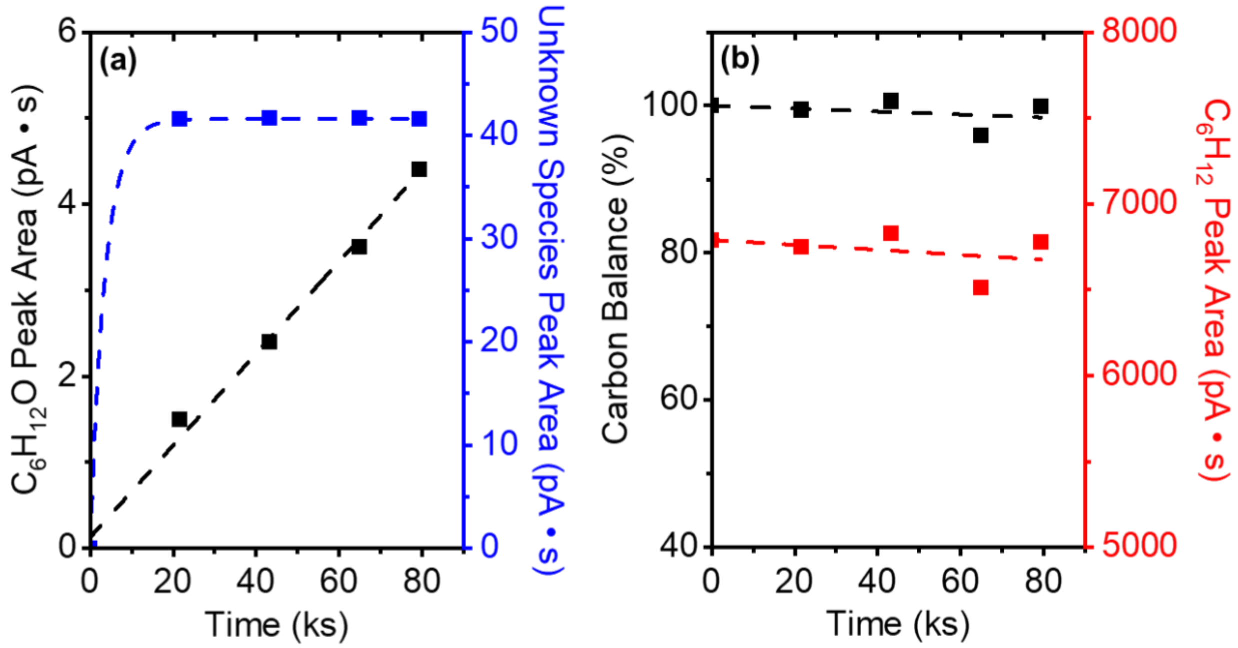

Figure S11. (a) Integrated areas of the $C_6H_{12}O$ (black) and unknown peak (blue) shown in **Figure S10**. (b) Carbon balance (black) and integrated areas of the C_6H_{12} peak (red) for the chromatograms shown in **Figure S9**.

S8. Calculating Epoxidation and O_2 Evolution Formation Rates and Faradaic Efficiency

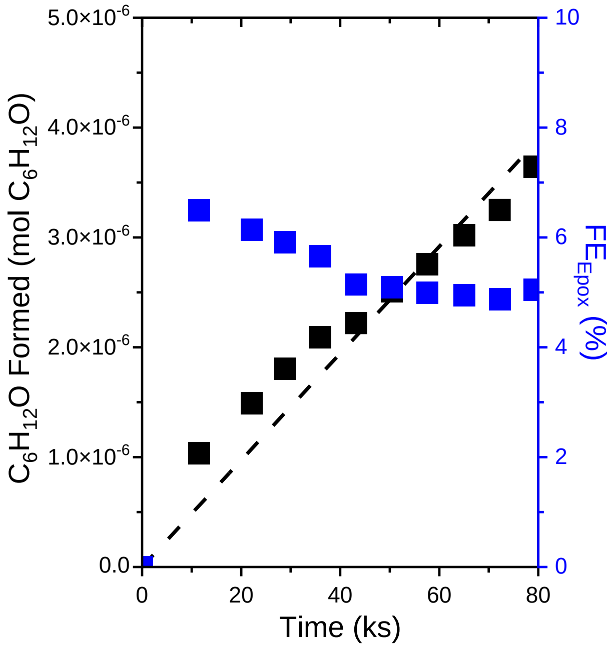

Figure S12. Representative $C_6H_{12}O$ formation (black) and epoxidation Faradaic efficiencies (FE_{Epox}, blue) as a function of time (1.31 $V_{Fc/Fc+}$, 0.1 M C_6H_{12}, 0.1 M $TBAClO_4$, 10 M H_2O, CH_3CN) over a 22-hour chronoamperometry measurement.

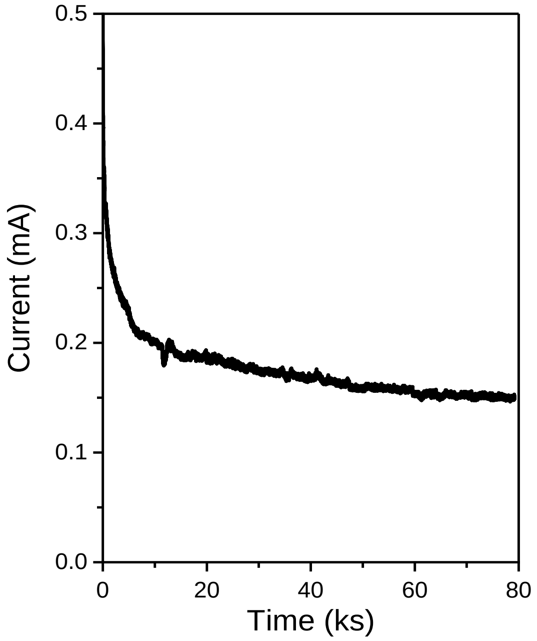

Figure S13. Representative chronoamperogram during an epoxidation rate measurement (1.31 $V_{Fc/Fc+}$, 0.1 M C_6H_{12}, 0.1 M $TBAClO_4$, 10 M H_2O, CH_3CN).

Figure S12 shows that $C_6H_{12}O$ forms, as determined by gas chromatography analysis, consistently over a 22-hour chronoamperometry measurement (1.31 $V_{Fc/Fc+}$, 0.1 M C_6H_{12}, 0.1 M $TBAClO_4$, 10 M H_2O, CH_3CN). Epoxidation rates are determined by taking a linear fit of the $C_6H_{12}O$ formed versus time data with the epoxide formed fixed at zero at time equal to zero. Epoxidation Faradaic efficiency (FE_{Epox}) is calculated by comparing the amount of $C_6H_{12}O$ formed ($n_{C_6H_{12}O,formed}$) to the theoretical amount of $C_6H_{12}O$ formed if all charge passed (Q) contributed to epoxidation, as shown in **Equation 1** in the main text:

$$FE_{Epox} = \frac{n_{C_6H_{12}O,formed}}{\frac{Q}{nF}} \tag{S7}$$

Where F is the Faraday constant (96,485 C mol^{-1}) and n is the number of electrons (e^-) required for the production of one $C_6H_{12}O$ molecule (two e^-). Q is determined through integration of the chronoamperogram (**Figure S13**) up until the time point of the respective sample. FE_{Epox} initially decreases (~20%) for the first 40 ks of the measurement before reaching a stable value. The total current decreases throughout the measurement, with the most significant current decay occurring before 30 ks.

The partial current density of epoxidation (i_{Epox}) is calculated by multiplying the total current density (i_{Total}) with FE_{Epox}:

$$i_{Epox} = i_{Total} * FE_{Epox} \tag{S8}$$

The partial current density of O_2 evolution (i_{OER}) is determined by subtracting i_{Epox} from i_{Total}:

$$i_{OER} = i_{Total} - i_{Epox} \tag{S9}$$

The rate of epoxidation to O_2 evolution (r_{O_2}) can be found using i_{Epox} and i_{OER} as follows:

$$\frac{r_{C_6H_{12}O}}{r_{O_2}} = \frac{n_{Epox} * i_{Epox}}{n_{OER} * i_{OER}} \tag{S10}$$

Where n_{OER} is the number of electrons required to produce one O_2 molecule (four e^-) and n_{Epox} is the number of electrons required for the production of one epoxide molecule (two e^-).

S9. Testing for External Mass Transfer Limitations

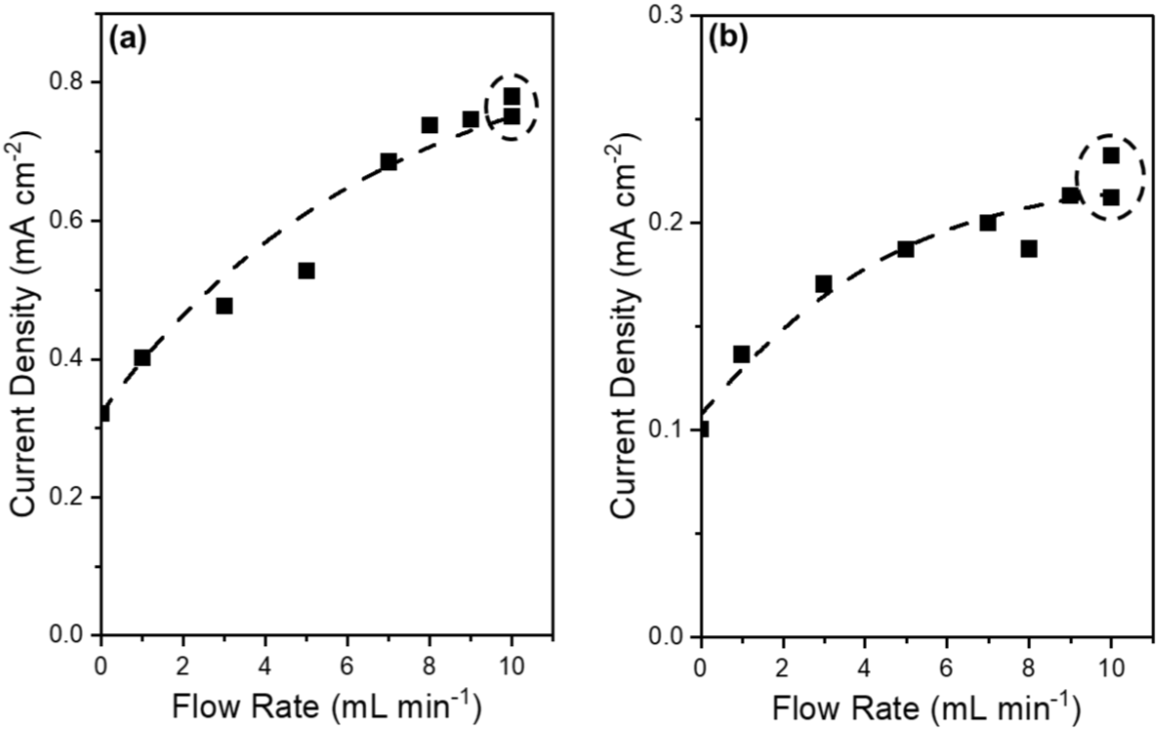

Figure S14. Current density as a function of flow rate in electrolytes without (0.1 M $TBAClO_4$, 10 M H_2O, CH_3CN, 1.41 $V_{Fc/Fc+}$) and with C_6H_{12} (0.1 M C_6H_{12}, 0.1 M $TBAClO_4$, 10 M H_2O, CH_3CN, 1.31 $V_{Fc/Fc+}$). Current density in (a) reflects only OER and in (b) includes both epoxidation and OER. The circled points signify the flow rate used for this study.

Figure S14 presents total current density as a function of electrolyte flow rate. To study intrinsic rates, the system must not be externally mass transfer limited (i.e., transport across the boundary layer from the bulk fluid to anode surface). Internal mass transfer limitations are not of concern because the anode is not porous. External mass transfer limitations are considered insignificant when changing the fluid convection (i.e., flow rate) does not change the reaction rate.[3, 4] Electrochemical rates, which display low current densities (< 1 mA cm^{-2}) depend negligibly on flow rates within the range of potentials and flow rates examined in this study, which suggests contributions from external mass transfer limitations minimally influence rate measurements.

Figure 1b provides further evidence that external mass transfer limitations do not convolute kinetic measurements. The charge passed in the oxidation feature from 0.65 to 0.94 $V_{Fc/Fc+}$, where both Au surface oxidation and C_6H_{12} oxidation occur, reaches saturation as [C_6H_{12}] increases at scan rates greater than 5 mV s^{-1}. This results from diffusion-induced external mass transfer limitations across the concentration boundary layer, which decreases with decreasing scan rate. The charge passed in the oxidation feature (0.65 to 0.94 $V_{Fc/Fc+}$) continues to increase as [C_6H_{12}] increases at scan rates lower than 5 mV s^{-1}, because the system no longer contains external mass transfer limitations related to C_6H_{12} diffusion. The system at slower scan rates reflects the system at constant potential, which achieves the limit of 0 mV s^{-1}.

S10. System for *In Situ* Raman Spectroscopy Measurements

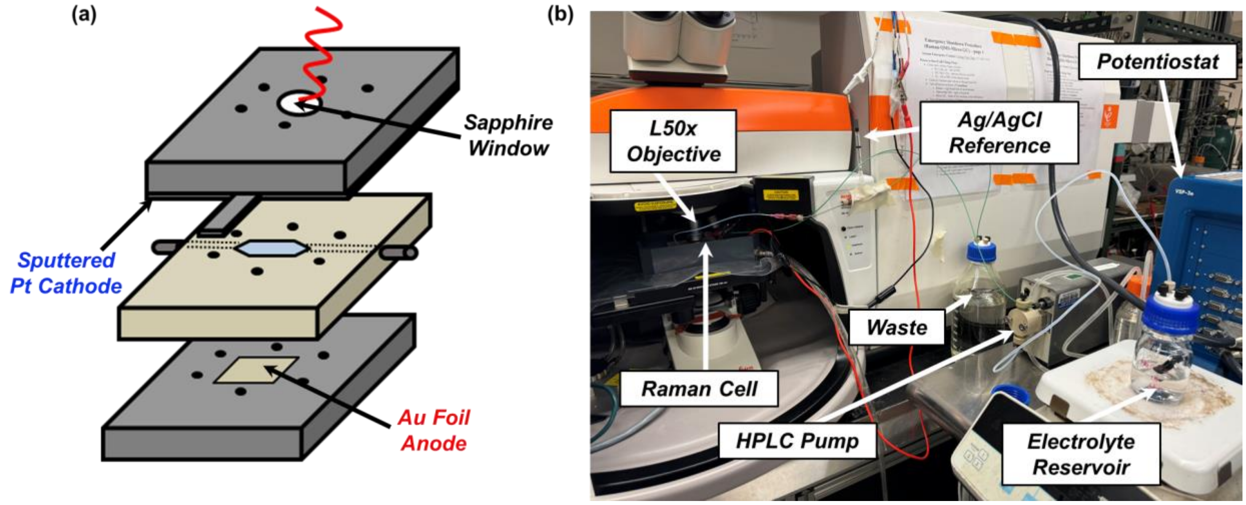

Figure S15. (a) Schematic of flow cell used for *in situ* Raman spectroscopy experiments. (b) Image of system used for *in situ* Raman measurements.

Figure S15a shows the sandwich-type flow cell used for all *in situ* Raman measurements. The Au foil anode was placed on a stainless-steel plate that acted as the current collector and was connected to the potentiostat with a banana plug. A PEEK flow plate containing a trapezoidal flow path (one cm^2 geometric area) was placed atop the anode. Needles (20 gauge) were punched into the flow plate for the purpose of electrolyte flow through the cell. A stainless-steel plate sputter coated with Pt (150 nm) that partially obstructed the trapezoidal flow path served as the cathode and contained a tab for connection to the potentiostat with an alligator clip. A stainless-steel plate with a recess to fit a window (Edmund Optics, sapphire, 2.54 cm diameter, 0.1 cm thick) was placed on top of the cathode plate to allow the laser to contact the Au anode. The distance between the Au anode and the top of the window (0.65 cm) allowed for focusing on the anode surface. O-rings (silicone or Viton) prevented the electrolyte from leaking by sealing between the Au anode and flow plate, flow plate and cathode plate, cathode plate and window plate, and window and window plate. PEEK screws were used to assemble the cell.

The Raman flow cell was placed in a secondary container under the objective lens (long 50x) as shown in **Figure S15b**. The laser (633.2 nm) illuminates the sample covered with constant electrolyte flow (one cm^3 min) in the cell to acquire SERS spectra. All Raman experiments were operated in a single-pass flow configuration. The same HPLC pump used for kinetic measurements pumped electrolyte (0.1 M $NaClO_4$ or 0.1 M KOH, H_2O; 0-0.1 M C_6H_2, 0.1 M $TBAClO_4$, 10 M H_2O, CH_3CN) from the electrolyte reservoir (media bottle, 100-1000 cm^3) to the flow cell. A female PEEK to male luer adapter connected the PEEK tubing from the HPLC pump to the polytetrafluoroethylene (PTFE) syringe tubing attached to the inlet of flow cell. Another female PEEK to male luer adapter connected the PTFE syringe tubing attached to the outlet of the flow cell to the PEEK tubing. This PEEK tubing was then connected to a polypropylene female luer tee using another adapter. A polypropylene male luer to male barb fitting connected to a small piece of PTFE tubing was attached to the tee opening perpendicular to the other tee openings. The

Ag/AgCl reference electrode (BASi, 3 M KCl, 7.5 cm length, 6 mm diameter) was placed within the PTFE tubing. The Ag/AgCl reference electrode was placed downstream of the Raman cell to prevent any potential contamination from Cl^- leaking from the electrode. The final tee opening connected to a male luer to female PEEK adapter, which connected to PEEK tubing that was attached to the waste container. The entire system operates at ambient temperature and pressure.

S11. Spectral Processing to Remove Artifact from Damaged Detector Element

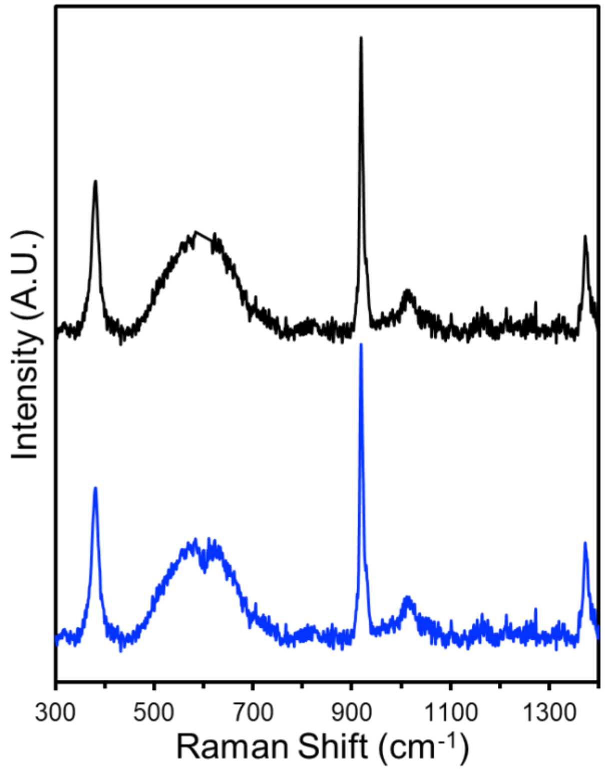

Figure S16. Steady-state Raman spectra of the electrochemically roughened Au anode in flowing electrolyte (1.31 $V_{Fc/Fc+}$, 0.1 M C_6H_{12}, 0.1 M $TBAClO_4$, 10 M H_2O, CH_3CN) without data processing (blue) and with correction to account for damaged detector element (black).

For all collected spectra, an inverse feature (595 cm^{-1}) appeared in the first third of the selected spectra window (210 to 1410 cm^{-1}), as shown in **Figure S16** in blue. This artifact arises from an airborne particle, such as pollen or dust, that reduces the sensitivity of one element on the charge coupled device detector of the spectrometer. The feature was removed using a standard software correction (Zap tool, WiRE Raman software). The black spectrum in **Figure S16** represents the correction to the blue Raman spectra accomplished by removing the artifact with the Zap tool. All reported spectra are processed to remove the inverse feature.

S12. Reagent Thermodynamic Activity Measurements

Thermodynamic activities of H_2O and C_6H_{12} in electrolytes of various compositions were determined by analysis of headspace vapor in equilibrium with the liquid electrolyte as described in Section 2.4 of the main text. Assuming ideal behavior of the vapor phase while considering non-ideality in the liquid phase, Raoult's Law is written as:

$$y_i P = p_i = x_i \gamma_i p_i^{sat} \quad \text{(S11)}$$

Here, y_i is the mole fraction of species i in the vapor phase, P is the total system pressure, p_i is the partial pressure of species i in the vapor phase, x_i is the mole fraction of species i in the liquid phase, γ_i is the activity coefficient of species i, and p_i^{sat} is the saturation pressure of species i. For a pure species, the mole fraction and activity coefficient will equal unity. Therefore, the partial pressure of the pure species (p_i^{sat}) will equal its saturation pressure:

$$p_{i,pure} = p_i^{sat} \quad \text{(S12)}$$

Combination of **Equation S11** and **Equation S12** leads to the following:

$$\frac{p_i}{p_{i,pure}} = x_i \gamma_i \quad \text{(S13)}$$

Injecting a volume of headspace vapor into a gas chromatograph equipped with a thermal conductivity detector to detect H_2O and a flame ionization detector to detect C_6H_{12} will yield chromatograms with peaks whose areas are proportional to the number of moles of H_2O and C_6H_{12} injected. If the temperature and injection volume of headspace vapor remain constant between injections, then the ratio of integrated peak areas between two injections should be equal to the ratio of their partial pressures by the ideal gas law. Therefore, **Equation S13** can be rewritten as:

$$\frac{A_i}{A_{i,pure}} = x_i \gamma_i \quad \text{(S14)}$$

Here, A_i is the integrated peak area of species i from an electrolyte sample and $A_{i,pure}$ is the integrated peak area of species i from a pure species sample. Rearranging **Equation S14** leads to the equation described in the main text (**Equation 2**) used to calculate activity coefficients for H_2O and C_6H_{12}:

$$\gamma_i = \frac{A_i}{A_{i,pure}} \cdot \frac{1}{x_i} \quad \text{(S15)}$$

This analysis was used to collect measurements of activity coefficients for H_2O and C_6H_{12} (**Figure S17**) for a variety of electrolyte compositions at ambient temperature (between 293-297 K depending on the building temperature that day). Vertical error bars represent the uncertainties of activity coefficient measurements propagated from the uncertainty of peak area ratios (determined by the standard deviation of triplicate samples) and uncertainty of H_2O or C_6H_{12} mole fraction (determined using tolerances of pipettes used to prepare electrolyte solutions).

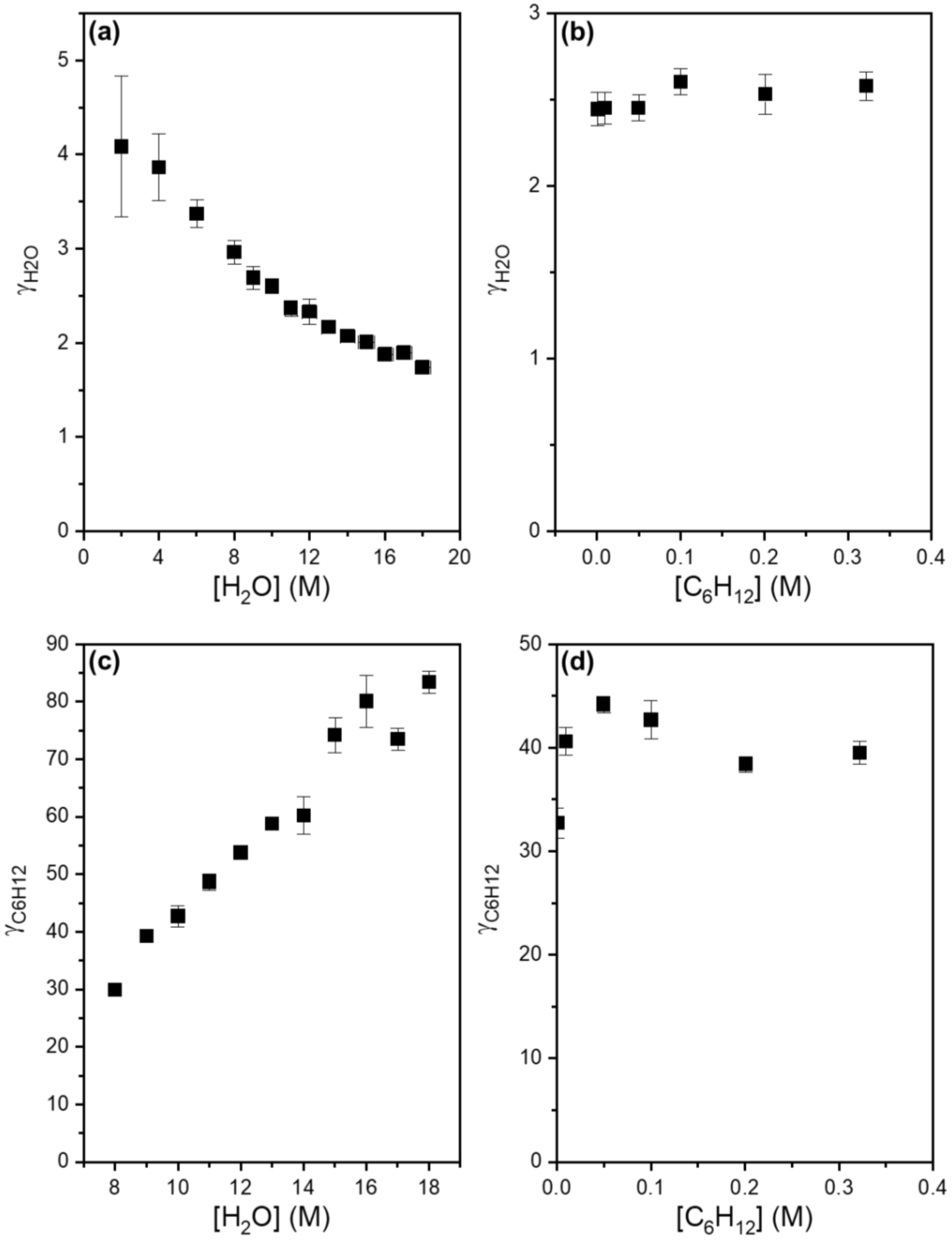

Figure S17. (a) H_2O activity coefficient as a function of [H_2O] (0.1 M C_6H_{12}, 0.1 M $TBAClO_4$, CH_3CN). (b) H_2O activity coefficient as a function of [C_6H_{12}] (0.1 M $TBAClO_4$, 10 M H_2O, CH_3CN). (c) C_6H_{12} activity coefficient as a function of [H_2O] (0.1 M C_6H_{12}, 0.1 M $TBAClO_4$, CH_3CN). (d) C_6H_{12} activity coefficient as a function of [C_6H_{12}] (0.1 M $TBAClO_4$, 10 M H_2O, CH_3CN).

After determining the activity coefficients for this range of electrolyte compositions, thermodynamic activities were calculated with reference to the standard state concentrations of each species where their activities are equal to unity (i.e., the pure species concentrations).

$$a_i = \frac{\gamma_i c_i}{c_{i,0}} \tag{S16}$$

Here, a_i is the thermodynamic activity of species i, c_i is the concentration of species i in the electrolyte solution, and $c_{i,0}$ is the concentration of pure species i. The activities of H_2O and C_6H_{12} as functions of [H_2O] and [C_6H_{12}] are displayed in **Figure S18**. The point at 0.32 M C_6H_{12} has been omitted, as the C_6H_{12} activity at this electrolyte composition was determined to be approximately 1.6, which does not make physical sense. This erroneous result may be associated with error due to 0.32 M C_6H_{12} being close to the solubility limit of C_6H_{12} in the aqueous CH_3CN electrolyte (0.1 M $TBAClO_4$, 10 M H_2O, CH_3CN).

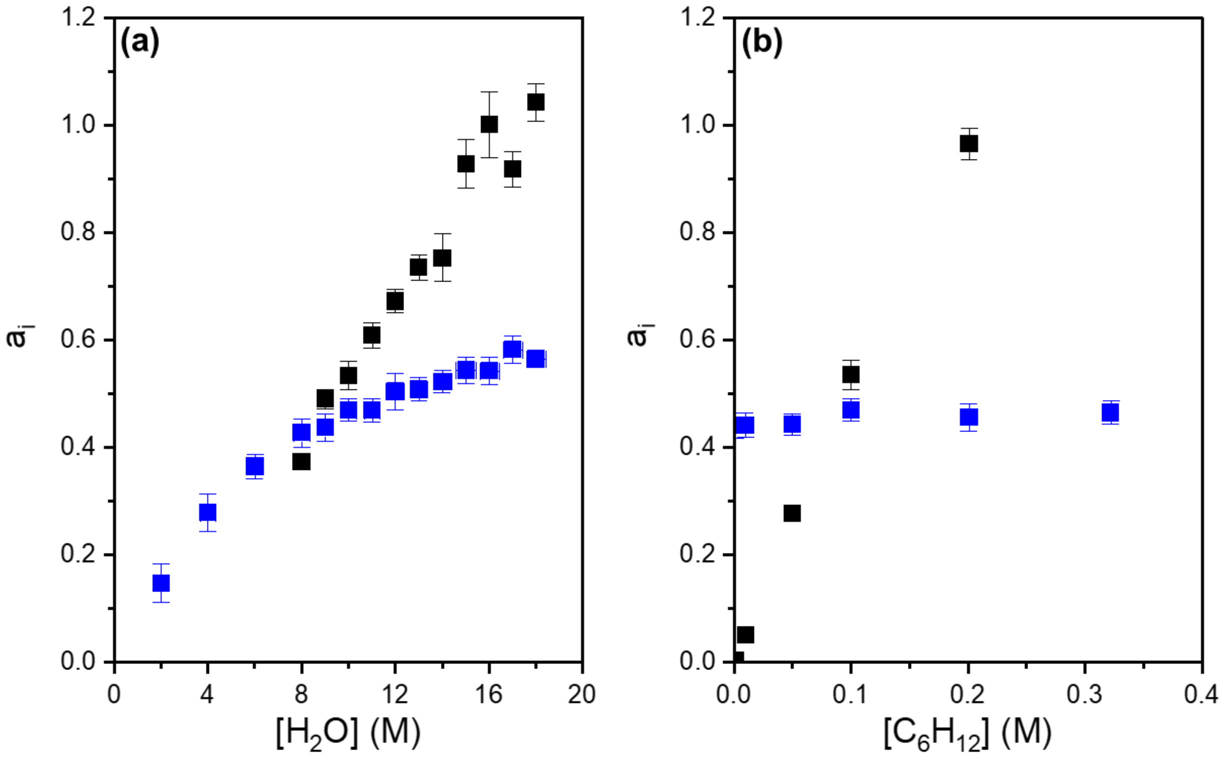

Figure S18. (a) H_2O (blue) and C_6H_{12} (black) activity as a function of [H_2O] (0.1 M C_6H_{12}, 0.1 M $TBAClO_4$, CH_3CN). (b) H_2O (blue) and C_6H_{12} (black) activity as a function of [C_6H_{12}] (0.1 M $TBAClO_4$, 10 M H_2O, CH_3CN).

S13. Analysis of Oxidation and Reduction Features in Cyclic Voltammograms

S13.1. Analysis of Cyclic Voltammetry Varying [C_6H_{12}]

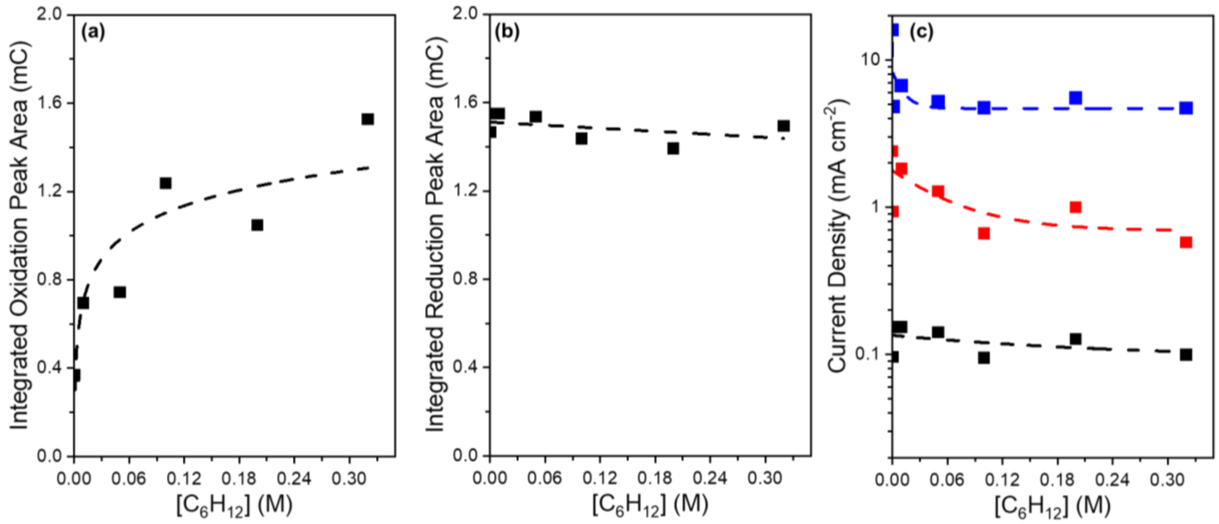

Figure S19. Integrated areas of (a) oxidation feature (0.65 to 0.94 $V_{Fc/Fc+}$) and (b) reduction feature (-0.5 to -0.05 $V_{Fc/Fc+}$) and (c) current density at 1.1 $V_{Fc/Fc+}$ (black), 1.31 $V_{Fc/Fc+}$ (red), and 1.46 $V_{Fc/Fc+}$ (blue) as a function of [C_6H_{12}] from cyclic voltammograms in Figure 1 of main text (0.1 M $TBAClO_4$, 10 M H_2O, CH_3CN, 10 mV s^{-1}).

Figures S19a and **S19b** show the total charge passed, calculated by integration of current with respect to time, in the oxidation feature (0.65 to 0.94 $V_{Fc/Fc+}$) and reduction feature (-0.5 to -0.05 $V_{Fc/Fc+}$) in **Figure 1** from the main text. **Figure S18a** reveals that the total charge passed in the oxidation feature (0.65 to 0.94 $V_{Fc/Fc+}$) increases by a factor of three from 0-0.1 M C_6H_{12} and then no longer changes at concentrations greater than 0.1 M C_6H_{12}. The initial increase in charge arises from more instances of H_2O activation for both C_6H_{12} and Au surface oxidation. Stabilization of total charge passed beyond 0.1 M C_6H_{12} occurs due to limitations on the quantity of C_6H_{12} that can diffuse to the surface within the time scale of the oxidation feature at set scan rate (10 mV s^{-1}). **Figure S19b** demonstrates that C_6H_{12} desorption does not occur at significant rates because the charge passed in the reduction feature (-0.5 to -0.05 $V_{Fc/Fc+}$) does not markedly vary with [C_6H_{12}]. The current density at potentials greater than 0.94 $V_{Fc/Fc+}$ initially decrease from 0-0.1 M C_6H_{12} and then no longer change at higher [C_6H_{12}] (**Figure S19c**). This illustrates that C_6H_{12} oxidation inhibits O_2 evolution and that both reactions compete for oxygen surface intermediates.

S13.2. Analysis of Cyclic Voltammetry Varying [H_2O]

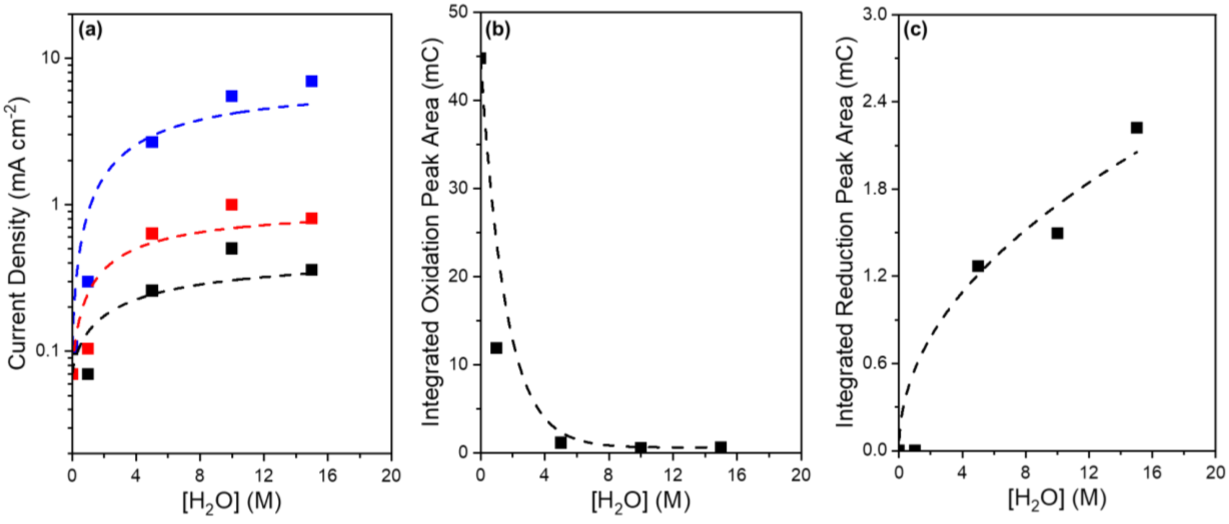

Figure S20. (a) Current density at 1.1 $V_{Fc/Fc+}$ (black), 1.31 $V_{Fc/Fc+}$ (red), and 1.46 $V_{Fc/Fc+}$ (blue) and integrated areas of (b) oxidation feature (0.65 to 0.94 $V_{Fc/Fc+}$) and (c) reduction feature (-0.5 to -0.05 $V_{Fc/Fc+}$) as a function of [H_2O] from cyclic voltammograms in Figure 2 of main text (0.1 M C_6H_{12}, 0.1 M $TBAClO_4$, CH_3CN, 10 mV s^{-1}).

Figure S20a shows that total current densities at potentials greater than 1.1 $V_{Fc/Fc+}$ increase by an order of magnitude from 0-5 M H_2O and then do not significantly change. This depicts the importance of electrochemical H_2O activation to form surface oxygen species for both C_6H_{12} oxidation and O_2 evolution. **Figures S20b** and **S20c** present the total charge passed, calculated by integration of current with respect to time, in the oxidation feature (0.65 to 1.18 $V_{Fc/Fc+}$) and reduction feature (-0.5 to -0.05 $V_{Fc/Fc+}$) in **Figure 2** from the main text. The total charge passed in the oxidation feature (0.65 to 1.18 $V_{Fc/Fc+}$) remains constant from 5-15 M H_2O but then greatly increases with decreasing [H_2O] (0-5 M H_2O). This increase in charge passed arises from dissolution of Au into the electrolyte and subsequent oxidation of the newly uncovered layer of Au atoms. At [H_2O] greater than 5 M, Au dissolution no longer occurs because H_2O and H_2O derived species displace organic species from the Au surface. The total charge passed in the reduction feature (-0.5 to -0.05 $V_{Fc/Fc+}$) significantly increases from 0-5 M H_2O as the reduction of surface Au oxide only occurs when the surface oxidizes. At higher [H_2O] (5-15 M) the total charge remains relatively constant due to the Au surface oxidation process being site limited.

S14. Au Dissolution Into Electrolyte

S14.1. Evidence of Au Dissolution at Low [H_2O]

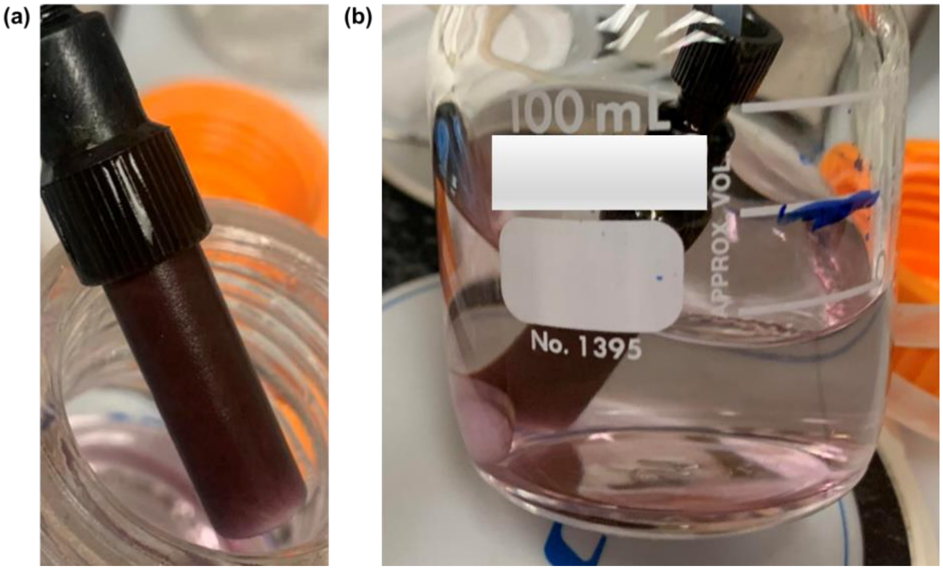

Figure S21. (a) HPLC pump solvent inlet filter and (b) electrolyte in reservoir after cyclic voltammetry with an electrolyte containing a low [H_2O] (0.1 M C_6H_{12}, 0.1 M $TBAClO_4$, 1 M H_2O, CH_3CN, 10 mV s^{-1}).

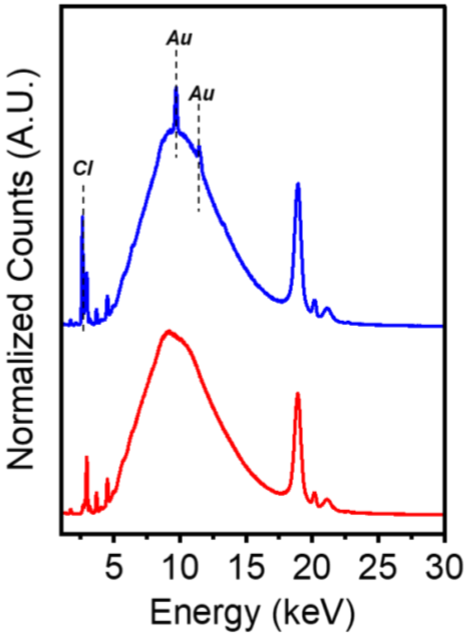

Figure S22. EDXRF spectra of filter before (red) and after (blue) cyclic voltammetry with an electrolyte containing low [H_2O] (0.1 M C_6H_{12}, 0.1 M $TBAClO_4$, 1 M H_2O, CH_3CN, 10 mV s^{-1}).

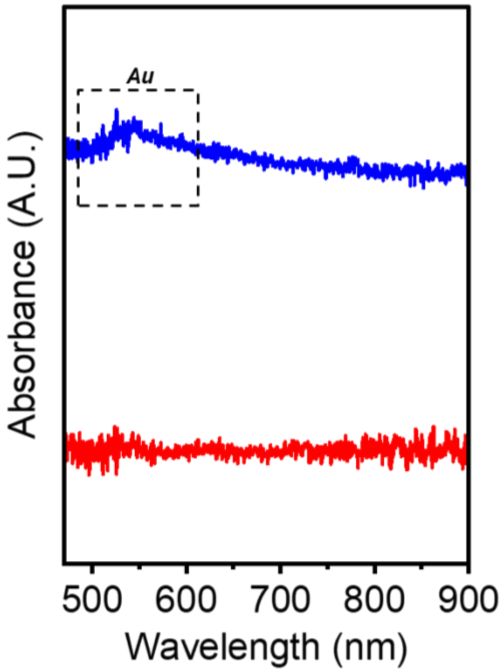

Figure S23. UV-Vis spectra of electrolyte containing low [H_2O] (0.1 M C_6H_{12}, 0.1 M $TBAClO_4$, 1 M H_2O, CH_3CN) before (red) and after (blue) cyclic voltammetry (10 mV s^{-1}).

Figure S21 shows an image of the pump inlet filter and electrolyte after cyclic voltammetry measurements in an electrolyte containing 1 M H_2O. The purple discoloration of the inlet filter, which was originally white, and electrolyte comes from Au nanoparticles. EDXRF analysis shows that features correlated to Au (9.70, 11.43 keV)[5] appear in the spectrum (**Figure S22**) after cyclic voltammetry, which validates that Au nanoparticles deposit on the pump inlet filter. ICP-OES analysis also confirms the presence of Au (21 μmol L^{-1}) in the electrolyte after cyclic voltammetry. UV-Vis spectroscopy (**Figure S23**) demonstrates that a feature at 545 nm related to surface plasmon resonance of Au nanoparticles (50-100 nm in diameter)[6] appears in the electrolyte after cyclic voltammetry measurements.

S14.2. Evidence for Lack of Au Dissolution at High [H_2O]

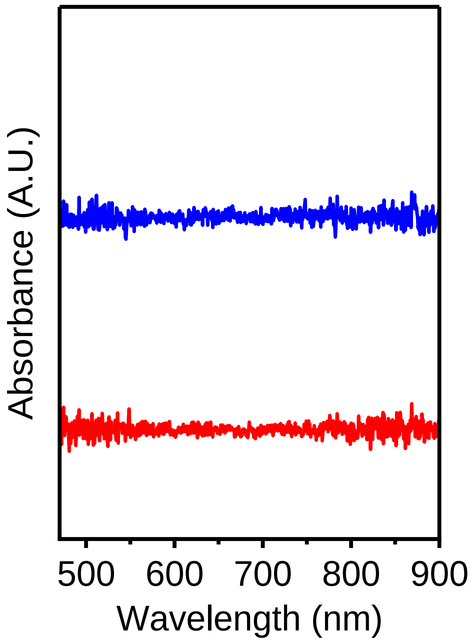

Figure S24. UV-Vis spectra of the electrolyte before (red) and after (blue) a 22 hour chronoamperometry measurement (1.31 $V_{Fc/Fc+}$, 0.1 M C_6H_{12}, 0.1 M $TBAClO_4$, 10 M H_2O, CH_3CN).

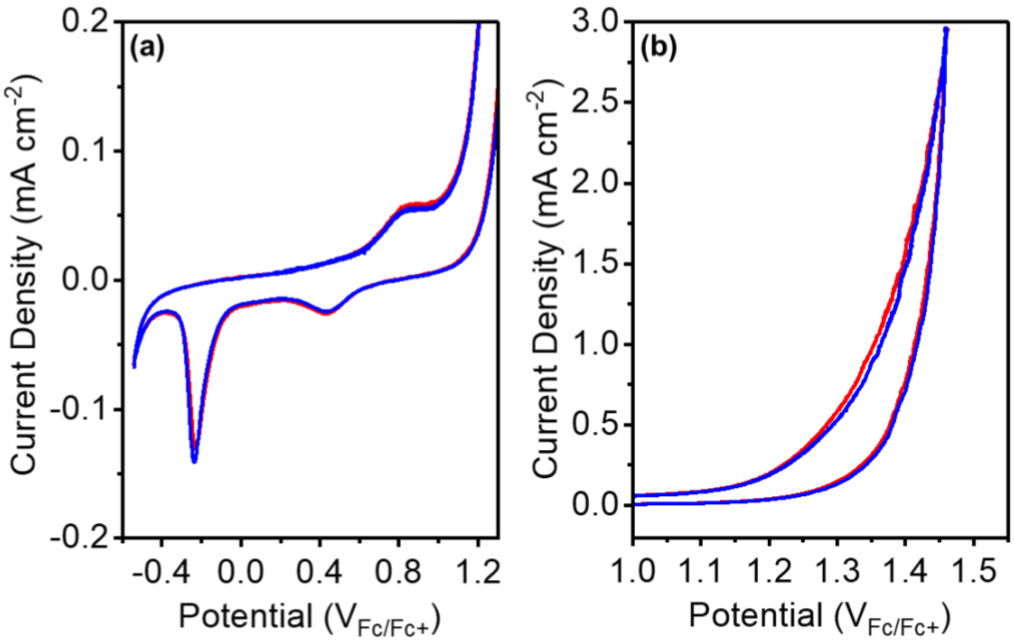

Figure S25. Cyclic voltammetry (10 mV s^{-1}) traces scaled to show features between (a) -0.6 to 1.3 $V_{Fc/Fc+}$ and (b) between 1.0 to 1.5 $V_{Fc/Fc+}$ before (red) and after (blue) a 22 hour chronoamperometry measurement (1.31 $V_{Fc/Fc+}$, 0.1 M C_6H_{12}, 0.1 M $TBAClO_4$, 10 M H_2O, CH_3CN).

UV-Vis and ICP-OES analysis on the electrolyte, ECSA measurements, and cyclic voltammetry measurements before and after a 24-hours chronoamperometry measurement provide evidence against Au dissolution and redeposition at high [H_2O] (10 M H_2O). UV-Vis (**Figure S24**) and ICP-OES demonstrate that no detectable amounts of Au nanoparticles exist in the electrolyte. Au dissolution and redeposition will roughen the surface and increase the ECSA. The ECSA does not change over the course of the chronoamperometry measurement (2.98 cm^2 before and 2.95 cm^{-2} after), which indicates that Au dissolution and redeposition does not occur. Previous reports demonstrate that Au dissolution and redeposition can lead to increases in the anodic oxidation peak area, increase in the anodic peak onset potential, and formation of a new cathodic peak (indicative of Au redeposition).[7-10] Cyclic voltammetry measurements before and after chronoamperometry display no significant changes in size or onset potentials of oxidation and reduction features (**Figure S25**). Collectively, these results demonstrate that at higher [H_2O] (> 8 M H_2O) Au dissolution and subsequent redeposition does not occur at significant rates and thus does not affect reported rates.

S15. Cyclic Voltammetry of Au in Neutral pH Aqueous Electrolyte

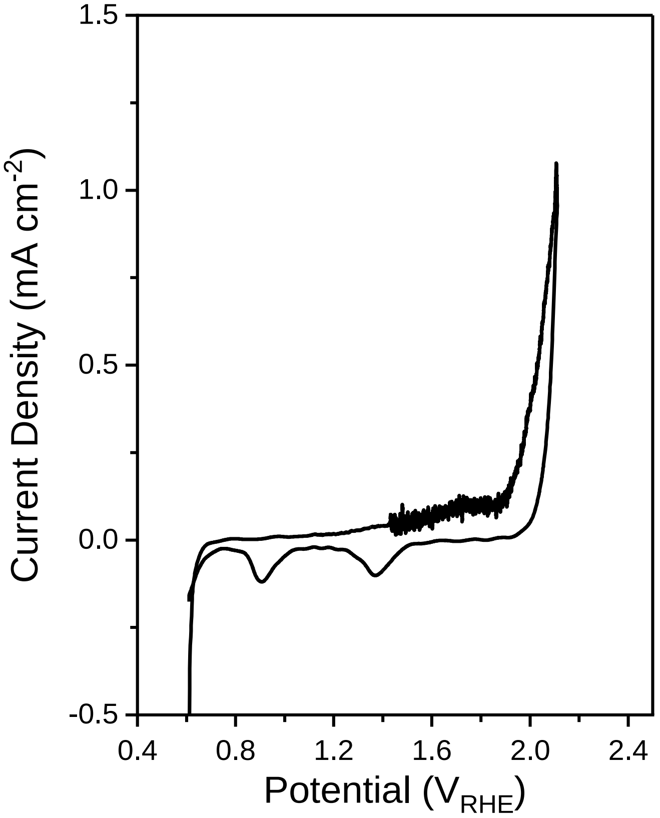

Figure S26. Cyclic voltammetry of Au collected in the electrochemical Raman cell (0.1 M $NaClO_4$, H_2O, 10 mV s^{-1}).

Figure S26 shows that Au surface oxidation occurs from 1.3 to 1.9 V_{RHE} and that OER begins at 1.9 V_{RHE}. The two surface reduction features correspond to the reduction of $Au_2O_{<3}$ to $Au(OH)_3$ (1.5 to 1.15 V_{RHE}) and $Au(OH)_3$ to metallic Au (0.9 to 0.8 V_{RHE}).

S16. Cyclic Voltammetry and Raman Spectra on Au in Alkaline Electrolyte

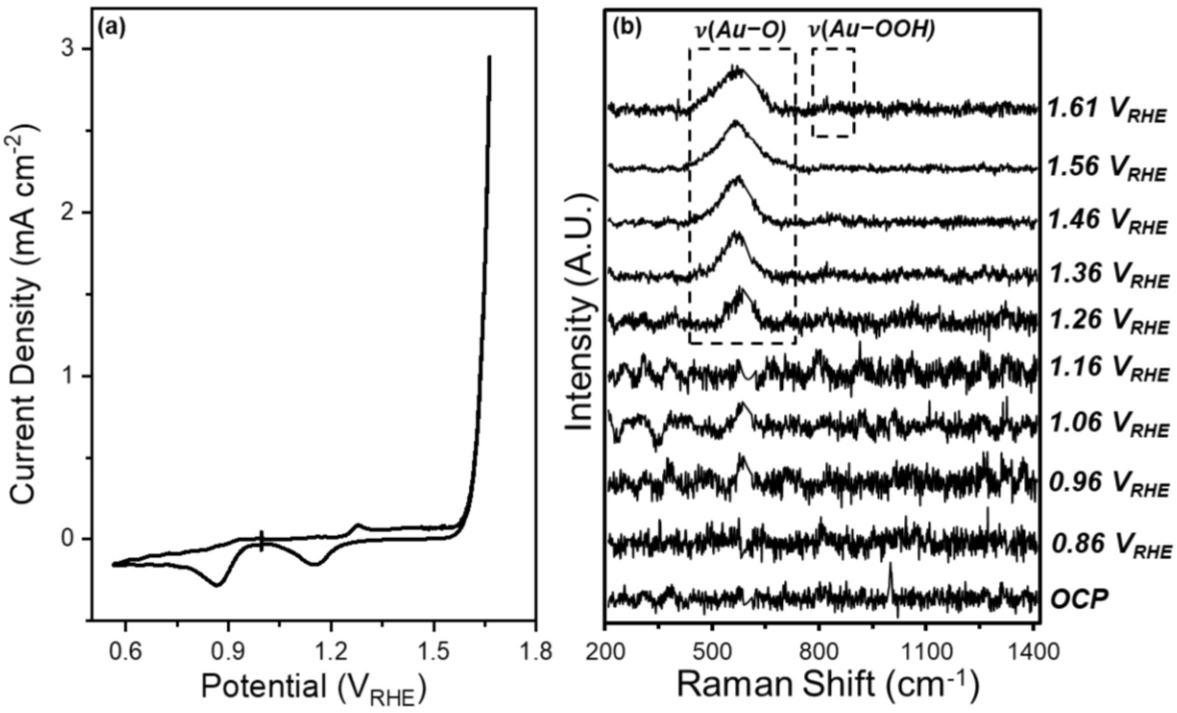

Figure S27. (a) Cyclic voltammetry of Au collected in the electrochemical Raman cell (0.1 M KOH, H_2O, 10 mV s^{-1}). (b) Potential-dependent steady-state Raman spectra of Au under flowing alkaline electrolyte (0.1 M KOH, H_2O).

Figure S27a shows that Au surface oxidation occurs from 1.25 to 1.3 V_{RHE} and that OER begins at 1.6 V_{RHE}. The two surface reduction features correspond to the reduction of $Au_2O_{<3}$ to $Au(OH)_3$ (1.2 to 1.05 V_{RHE}) and $Au(OH)_3$ to metallic Au (0.95 to 0.7 V_{RHE}). **Figure S27b** depicts the potential-dependent steady-state spectra of Au under flowing alkaline electrolyte (0.1 M KOH, H_2O). A feature centered at 580 cm^{-1} that corresponds to ν(Au-O) of monoatomic O* appears at all potentials greater than 1.25 V_{RHE}. This confirms Au surface oxidation to $Au_2O_{<3}$. A feature (820 cm^{-2}) assigned to the ν(O-O) mode of OOH* appears at potentials greater than the onset of OER (1.6 V_{RHE}).

S17. Extended Scan Steady-State Raman Spectra

S17.1. Raman Spectra in Neutral pH Aqueous Electrolyte

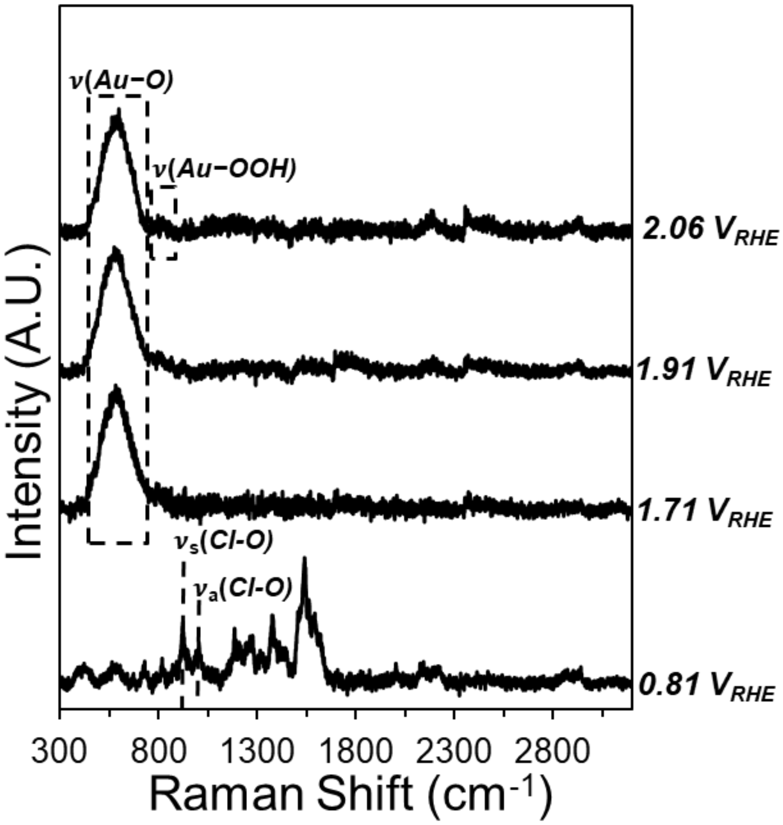

Figure S28. Potential-dependent steady-state Raman spectra of Au under flowing aqueous electrolyte (0.1 M $NaClO_4$, H_2O).

Figure S28 shows the extended scan potential-dependent steady-state Raman spectra of Au under flowing aqueous electrolyte (0.1 M $NaClO_4$, H_2O). Two vibrational modes of ClO_4^- appear at 925 cm^{-1} ν_s(Cl-O) and 1005 cm^{-1} ν_a(Cl-O) at potentials below the onset of Au surface oxidation. At potentials greater than the onset of Au surface oxidation (1.5 V_{RHE}) a feature related to the ν(Au-O) of monoatomic O* (580 cm^{-1}) remains present in the spectra. A feature (820 cm^{-2}) assigned to the ν(O-O) mode of OOH* appears at potentials greater than the onset of OER (1.9 V_{RHE}). No features appear at wavenumbers greater than 820 cm^{-1} at oxidizing potentials in the aqueous electrolyte.

S17.2. Raman Spectra in Aqueous-Organic Electrolyte in the Absence of C_6H_{12}

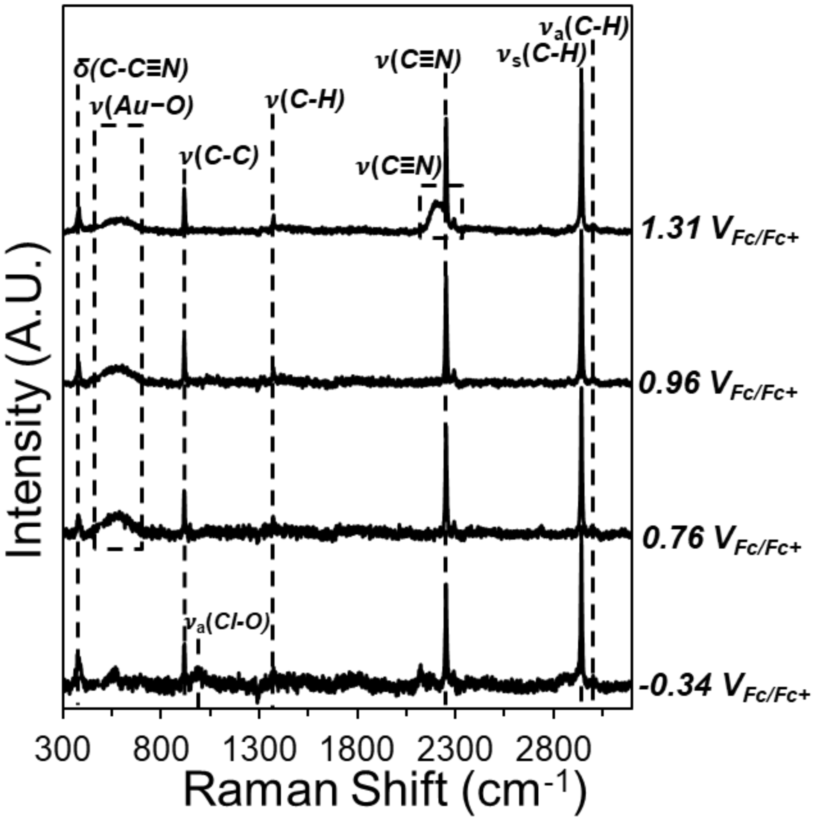

Figure S29. Potential-dependent steady-state Raman spectra of Au under flowing aqueous-organic electrolyte without C_6H_{12} (0.1 M $TBAClO_4$, 10 M H_2O, CH_3CN).

Figure S29 shows the extended scan potential-dependent steady-state Raman spectra of Au under flowing aqueous-organic electrolyte without C_6H_{12} (0.1 M $TBAClO_4$, 10 M H_2O, CH_3CN). At potentials greater than the onset of Au surface oxidation (0.65 $V_{Fc/Fc+}$) a feature related to the ν(Au-O) of monatomic O* (580 cm^{-1}) remains present in the spectra. At potentials lower than Au surface oxidation a feature that reflects ν_a(Cl-O) of ClO_4^- in the electrochemical double layer appears but does not appear at higher potentials. This may stem from the increased intensity of other vibrational modes. Several sharp features (380 cm^{-1}, 918 cm^{-1}, 1375 cm^{-1}, 2250 cm^{-1}, 2950 cm^{-1}, 3000 cm^{-1}) related to different vibrational modes of CH_3CN (δ(C-C≡N), ν(C-C), ν(C-H), ν(C≡N), ν_s(C-H), ν_a(C-H)) appear at all applied potentials, which indicates the presence of CH_3CN in the electrochemical double layer. A broad feature at 2200 cm^{-1} attributed to the ν(C≡N) mode of CH_3CN coordinated to Lewis acidic Au^{n+} surface sites materializes at 1.31 $V_{Fc/Fc+}$.

S17.3. Raman Spectra in Aqueous-Organic Electrolyte in the Presence of C_6H_{12}

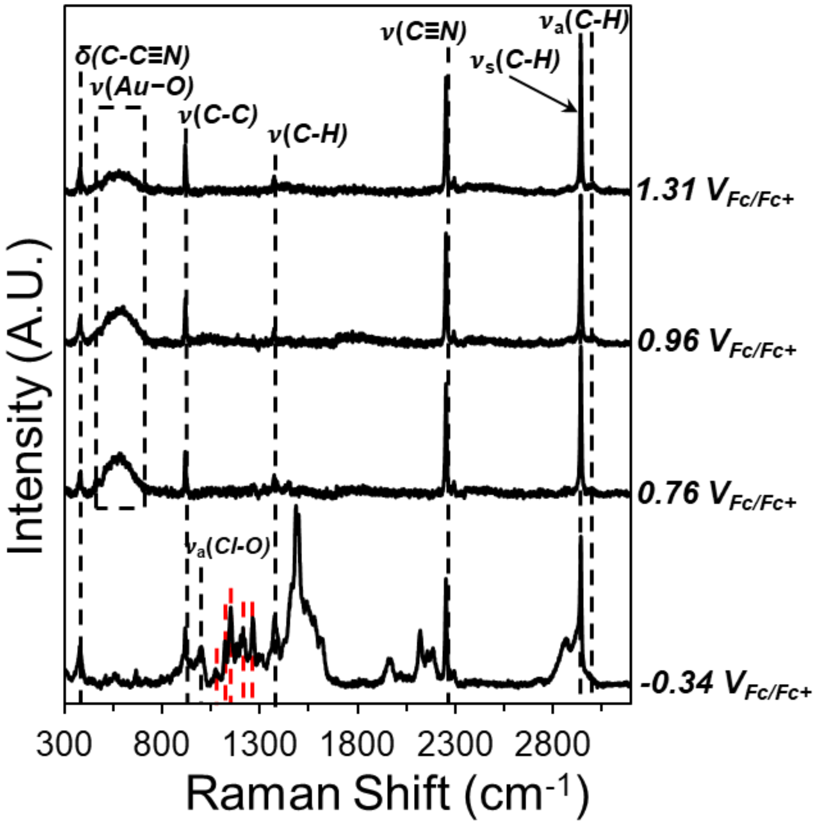

Figure S30. Potential-dependent steady-state Raman spectra of Au under flowing aqueous-organic electrolyte with C_6H_{12} (0.1 M C_6H_{12}, 0.1 M $TBAClO_4$, 10 M H_2O, CH_3CN). The red dashed lines correspond to vibrational modes of liquid-phase C_6H_{12}.

Figure S30 shows the extended scan potential-dependent steady-state Raman spectra of Au under flowing aqueous-organic electrolyte with C_6H_{12} (0.1 M C_6H_{12}, 0.1 M $TBAClO_4$, 10 M H_2O, CH_3CN). At potentials greater than the onset of Au surface oxidation (0.65 $V_{Fc/Fc+}$) a feature related to the ν(Au-O) of monoatomic O* (580 cm^{-1}) remains present in the spectra. The same features observed in the absence of C_6H_{12} corresponding to CH_3CN and ClO_4^- in the electrochemical double layer appear in the presence of C_6H_{12}. At -0.34 $V_{Fc/Fc+}$ five features (1080 cm^{-1}, 1115 cm^{-1}, 1150 cm^{-1}, 1210 cm^{-1}, 1285 cm^{-1}) that represent the ν(C-C), ν(C-C), ν(C-C), τ(C-H sp^3), and ρ(C-H sp^2) of C_6H_{12} appear. These features do not appear at higher potentials, likely due to the structure of the electrochemical double layer.

S18. Peak Assignments of Features in Raman Spectra

Table S3. Assignment of Raman features and corresponding vibrational modes.

Raman Shift (cm^{-1})	Species	Assignment	References
380	CH_3CN	C-C≡N bend	[11], [12]
580	O*	Au-O stretching	[13]-[19]
820	OOH*	O-O stretching	[16]-[18]
918	CH_3CN	C-C stretching	[11], [12]
925	ClO_4^-	Symmetric Cl-O stretching	[20], [21]
1005	ClO_4^-	Asymmetric Cl-O stretching	[22]-[25]
1080	C_6H_{12}	C-C stretching	[26]
1115	C_6H_{12}	C-C stretching	[26]
1150	C_6H_{12}	C-C stretching	[26]
1210	C_6H_{12}	C-H sp^3 out-of-plane twisting	[26]
1285	C_6H_{12}	C-H sp^2 in-plane rocking	[26]
1375	CH_3CN	C-H stretching	[11], [12]
2200	CH_3CN*	C≡N stretching	[27], [28], [29]
2250	CH_3CN	C≡N stretching	[11], [12], [27], [28], [29]
2950	CH_3CN	Symmetric C-H stretching	[11], [12]
3000	CH_3CN	Asymmetric C-H stretching	[11], [12]

Table S3 lists the peak assignments for intermediates and surface species observed on the Au anode in the aqueous (0.1 M $NaClO_4$, H_2O) and aqueous-organic electrolytes (0 or 0.1 M C_6H_{12}, 0.1 M $TBAClO_4$, 10 M H_2O, CH_3CN) in **Figure 3** of the main text. Electrochemical H_2O activation results in the adsorption of atomic oxygen (O*) on Au and the ν(Au-O) corresponding to O* is observed at 580 cm^{-1}.[13-19] The O* then oxidizes through electrochemical reaction with H_2O to form OOH*, whose ν(Au-OOH) feature appears in the spectra at 820 cm^{-1} at oxidizing conditions.[16-18] In the aqueous electrolyte, ClO_4^- present in the electrochemical double layer displays a feature at 925 cm^{-1} related to ν_s(Cl-O).[20, 21] For both the aqueous and aqueous-organic electrolytes, the asymmetric stretching mode of ClO_4^- appears at 1005 cm^{-1} before the onset of surface Au oxidation.[22-25] Sharp features assigned to different vibrational modes of CH_3CN appear at all applied potentials in the aqueous-organic electrolyte. These include the C-C≡N bending (380 cm^{-1}), C-C stretching (918 cm^{-1}), C-H stretching (1375 cm^{-1}), C≡N stretching (2250 cm^{-1}), symmetric C-H stretching (2950 cm^{-1}), and asymmetric C-H stretching (3000 cm^{-1}).[11, 12, 27-29] A broad feature centered at 2200 cm^{-1} that appears in the absence of C_6H_{12} at 1.31 $V_{Fc/Fc+}$ represents the adsorption of CH_3CN to the Lewis acidic Au^{n+} surface sites.[27-29] In the aqueous-organic electrolyte with features at 1080 cm^{-1}, 1115 cm^{-1}, 1150 cm^{-1}, 1210 cm^{-1}, and 1285 cm^{-1}, which correspond to the ν(C-C), ν(C-C), ν(C-C), τ(C-H sp^3), and ρ(C-H sp^2) of C_6H_{12} respectively, appear at potentials lower than the onset of Au surface oxidation.[26]

S19. Comparison of Raman Spectra in Electrolytes With and Without C_6H_{12}

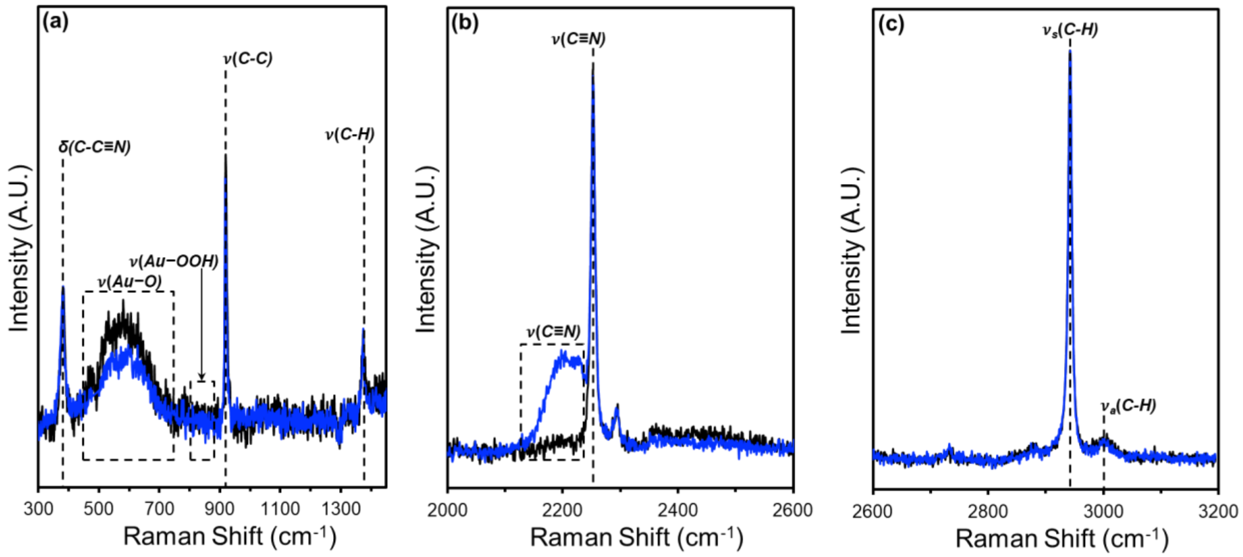

Figure S31. Overlaid steady-state Raman spectra of Au under flowing electrolyte (1.31 $V_{Fc/Fc+}$, 0 or 0.1 M C_6H_{12}, 0.1 M $TBAClO_4$, 10 M H_2O, CH_3CN) with (blue) and without (black) C_6H_{12}. Spectra are scaled to show features between (a) 300 to 1450 cm^{-1}, (b) 2000 to 2600 cm^{-1}, and (c) 2600 to 3200 cm^{-1}.

Figure S31 compares Raman spectra of Au in the absence and presence of C_6H_{12} (1.31 $V_{Fc/Fc+}$, 0 or 0.1 M C_6H_{12}, 0.1 M $TBAClO_4$, 10 M H_2O, CH_3CN). In the absence of C_6H_{12} a broad feature centered at 2200 cm^{-1} appears as a shoulder to the feature of the C≡N stretching mode (2250 cm^{-1}) of CH_3CN. We assign the feature at 2200 cm^{-1} to the C≡N stretching mode of CH_3CN adsorbed onto the Lewis acidic Au. The presence of C_6H_{12} disrupts CH_3CN adsorption onto Au as the broad shoulder does not appear with electrolytes containing C_6H_{12}. The addition of C_6H_{12} to the electrolyte does not lead to any additional features or changes beyond the absence of the feature related to CH_3CN adsorption. The absence of features attributed to adsorbed or solution-phase C_6H_{12} likely arise from the lower concentrations of C_6H_{12} in comparison to concentrations of CH_3CN and other species in the electrolyte.

S20. Distinguishing Between ν(Cl-O) and ν(C-C) in the Aqueous and Aqueous-Organic Electrolytes

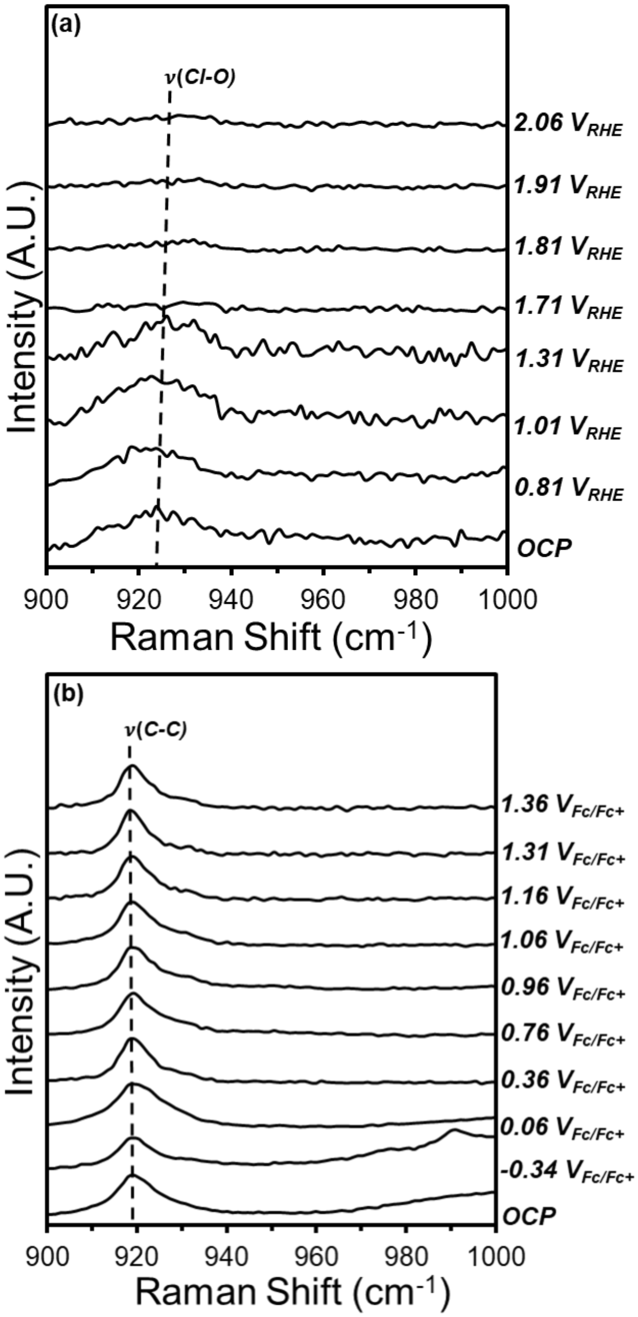

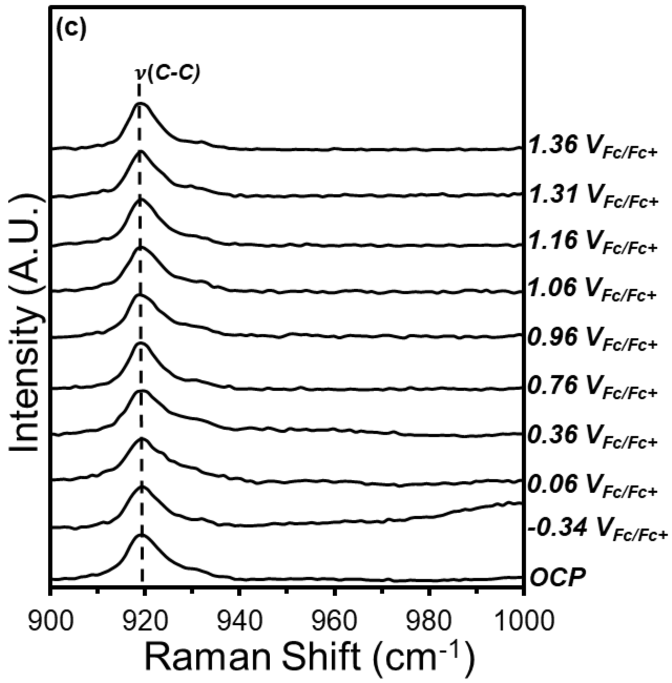

Figure S32. Potential-dependent steady-state Raman spectra of Au under flowing (a) aqueous electrolyte (0.1 M $NaClO_4$, H_2O) and aqueous-organic electrolytes (b) without (0.1 M $TBAClO_4$, 10 M H_2O, CH_3CN) and (c) with C_6H_{12} (0.1 M C_6H_{12}, 0.1 M $TBAClO_4$, 10 M H_2O, CH_3CN).

Figure S32 shows spectra of Au under aqueous (0.1 M $NaClO_4$, H_2O) and aqueous-organic (0 or 0.1 M C_6H_{12}, 0.1 M $TBAClO_4$, 10 M H_2O, CH_3CN) electrolytes. In the aqueous electrolyte a feature appears at 925 cm^{-1} at the open circuit potential and redshifts with applied oxidizing potentials. We assign this feature to the ν_s(Cl-O) of ClO_4^- derived from the $TBAClO_4$ supporting electrolyte. In the aqueous-organic electrolytes, a feature appears at 918 cm^{-1} and blueshifts with applied oxidizing potentials. This feature represents the C-C stretching mode of CH_3CN. The differences in peak centers and shifts with applied potentials allows for the distinction of ν_s(Cl-O) and ν(C-C) in the aqueous and aqueous-organic electrolyte. We note that in both the aqueous and aqueous-organic electrolytes, the feature related to the asymmetric stretching of ClO_4^- remains present at potentials lower than the onset of Au surface oxidation (**Figures 3, S28, S29, S30**).

S21. Product Distribution of Epoxidation with $H_2{}^{18}O$

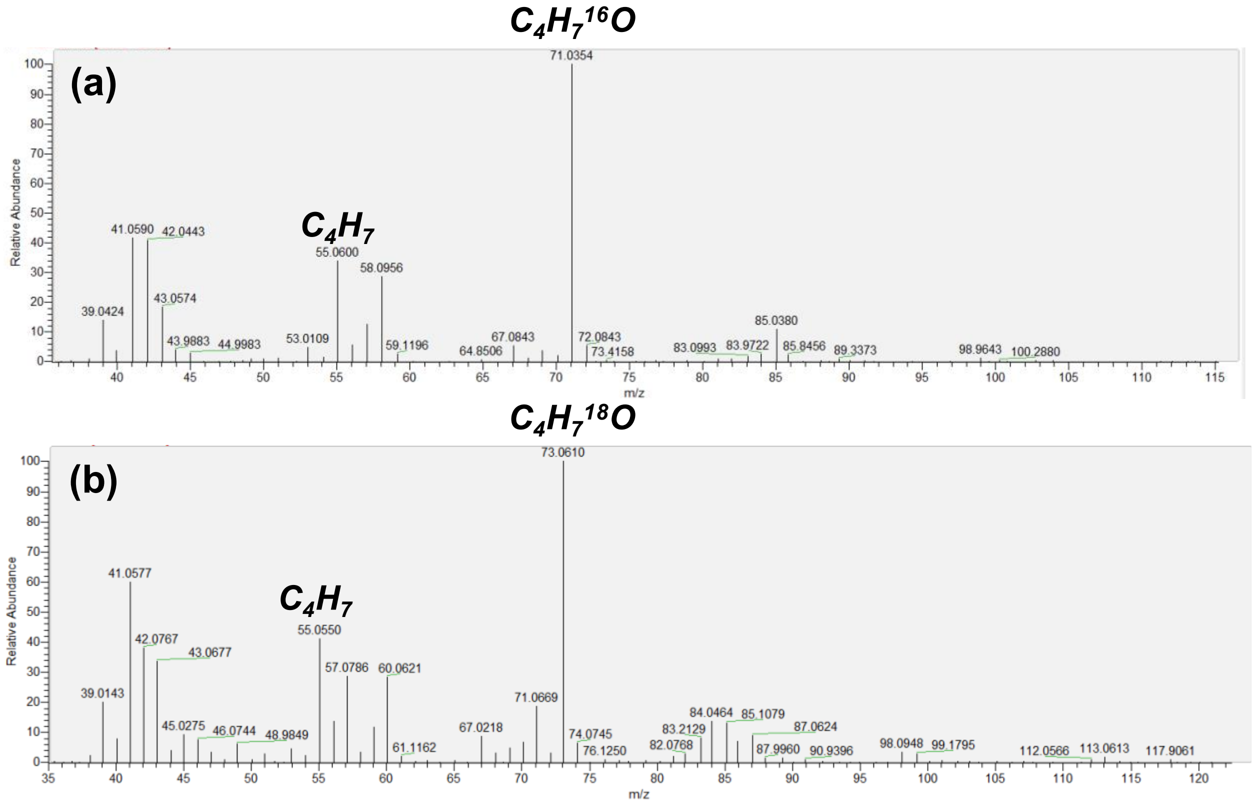

Figure S33. GC-MS spectrum of $C_6H_{12}O$ product derived from epoxidation in an electrolyte with (a) $H_2{}^{16}O$ (1.31 $V_{Fc/Fc+}$, 0.25 M C_6H_{12}, 10 M H_2O, 0.1 M $TBAClO_4$, CH_3CN) and (b) $H_2{}^{18}O$ (1.31 $V_{Fc/Fc+}$, 0.25 M C_6H_{12}, 10 M $H_2{}^{18}O$, 0.1 M $TBAClO_4$, CH_3CN).

Figure S33 shows GC-MS spectrum after chronoamperometry in an electrolyte with $H_2{}^{16}O$ (1.31 $V_{Fc/Fc+}$, 0.25 M C_6H_{12}, 10 M H_2O, 0.1 M $TBAClO_4$, CH_3CN) and $H_2{}^{18}O$ (1.31 $V_{Fc/Fc+}$, 0.25 M C_6H_{12}, 10 M $H_2{}^{18}O$, 0.1 M $TBAClO_4$, CH_3CN). $C_6H_{12}O$ has a C_4H_7O and C_4H_7 electron ion impact at a mass-to-charge ratio of 71 and 55, respectively. The prominent ion peak (C_4H_7O) shifts from a mass-to-charge ratio of 71 to 73, respectively, which suggests the presence of ^{18}O in $C_6H_{12}O$ when the electrolyte contains $H_2{}^{18}O$. This indicates that H_2O serves as the oxygen atom source during epoxidation on Au anodes.

S22. Derivation of Rate Expressions for C_6H_{12} Epoxidation and O_2 Evolution

S22.1. C_6H_{12} Reaction with O* *via* Eley-Rideal Mechanism

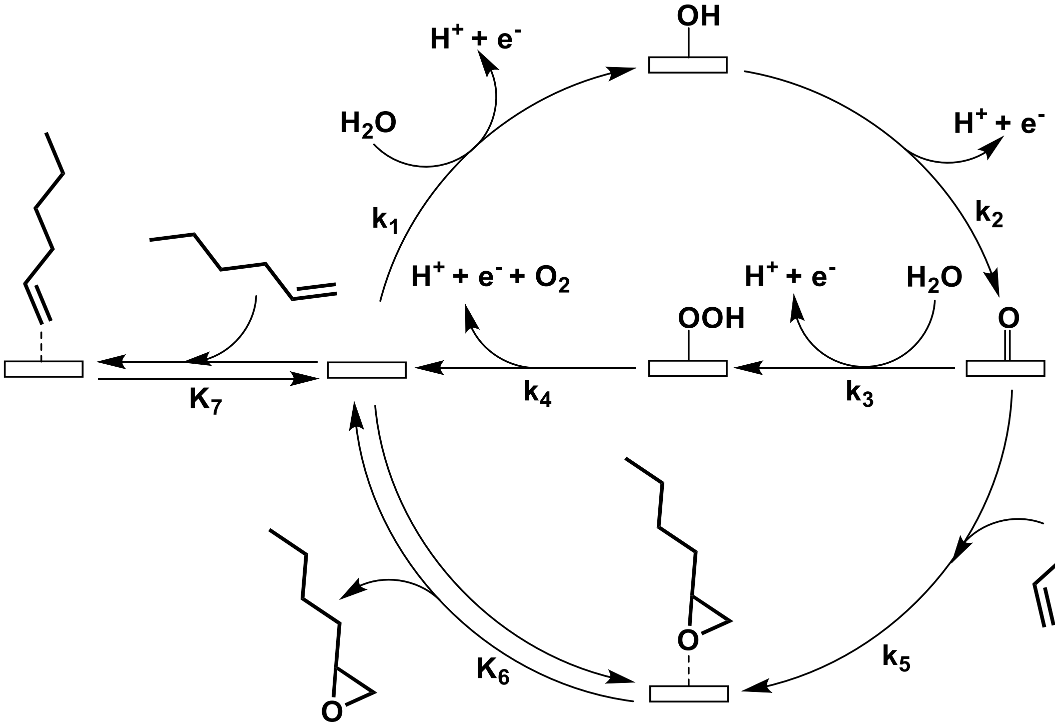

Scheme S1. Proposed catalytic cycles for C_6H_{12} epoxidation with O* species derived from electrochemical H_2O activation and O_2 evolution. All adsorption steps and proton-electron transfer steps are currently assumed as reversible and irreversible, respectively.

The rate expression for the steady-state formation of the epoxide product corresponds to the net rate of step 6 and step 5, in which the O* reacts to form $C_6H_{12}O$*:

$$r_{C_6H_{12}O} = r_6 - r_{-6} = r_5 = \; k_5\,[C_6H_{12}][O\,*] \tag{S17}$$

where k_i and k_{-i} are the forward and reverse rate constants for step *i*. The pseudo-steady state hypothesis (PSSH) can be applied to the number of O* species to derive an expression for [O*] that only contains rate constants and concentrations of species in the fluid (i.e., C_6H_{12}, H_2O). PSSH allows for the assumption that the net rate of active intermediates (i.e., $C_6H_{12}O$*, OH*, O*, OOH*) equals zero because the highly energetic and reactive intermediates undergo consumption *via* reaction at virtually the same rate as formation. Performing a mass balance and applying PSSH on O* yields the following expression:

$$r_{O*} = 0 = r_2 - r_3 - \; r_5 = k_2[OH\,*] - k_3[O\,*]\,[H_2O] - k_5[O\,*]\,[C_6H_{12}] \tag{S18}$$

Where [*] is the number of available Au active sites that can bind and react with a species in solution and $[H^+]$ is the concentration of protons. The following equation for [O*] can be derived by rearranging **Equation S18**:

$$[O*] = \frac{k_2[OH*]}{k_5[C_6H_{12}] + k_3[H_2O]} \tag{S19}$$

Application of the pseudo-steady state hypothesis (PSSH) and mass balance to the number of OH* species yields:

$$r_{OH*} = 0 = r_1 - r_2 = k_1[H_2O]\,[*] - k_2[OH*] \tag{S20}$$

Equation S20 can then be rearranged to:

$$[OH*] = \frac{k_1[H_2O]\,[*]}{k_2} \tag{S21}$$

Equation S19 combines with **Equation S21** to result in the following expression for [O*]:

$$[O*] = \frac{k_2[OH*]}{k_5[C_6H_{12}] + k_3[H_2O]} = \frac{k_2\frac{k_1[H_2O]\,[*]}{k_2}}{k_5[C_6H_{12}] + k_3[H_2O]} = \frac{k_1[H_2O]\,[*]}{k_5[C_6H_{12}] + k_3[H_2O]} \tag{S22}$$

The substitution of **Equation S22** into **Equation S17** yields the following expression for $C_6H_{12}O$ formation:

$$r_{C_6H_{12}O} = \frac{k_1k_5[C_6H_{12}][H_2O]\,[*]}{k_5[C_6H_{12}] + k_3[H_2O]} \tag{S23}$$

An expression for [*] derives from the sum of all likely surface intermediates and recognition that the total number of active sites ($[L]$) remains constant:

$$[L] = [*] + [OH^*] + [O^*] + [OOH^*] + [C_6H_{12}O^*] + [C_6H_{12}{}^*] \tag{S24}$$

where [*x**] is the number of adsorbed species *x**. The following equations show the application off a mass balance and PSSH on [OOH*], [$C_6H_{12}O$*], and [C_6H_{12}*]:

$$r_{OOH*} = 0 = r_3 - r_4 = k_3[O*][H_2O] - k_4[OOH*] \tag{S25}$$

$$r_{C_6H_{12}O*} = 0 = r_5 - r_6 + r_{-6} = k_5[O*]\,[C_6H_{12}] - k_6[C_6H_{12}O*] + k_{-6}[C_6H_{12}O] \tag{S26}$$

$$r_{C_6H_{12}*} = 0 = r_7 - r_{-7} = -k_7[C_6H_{12}][*] + k_{-7}[C_6H_{12}*] \tag{S27}$$

Rearranging **Equation S25** and combining with **Equation S21** leads to the following expression for [OOH*]:

$$[OOH*] = \frac{k_3[O*][H_2O]}{k_4[*]} = \frac{k_3\frac{k_1[H_2O]\,[*]}{k_5[C_6H_{12}] + k_3[H_2O]}[H_2O]}{k_4[*]} = \frac{k_1k_3\,[H_2O]^2[*]}{k_4(k_5[C_6H_{12}] + k_3[H_2O])} \tag{S28}$$

We assume that $C_6H_{12}O$ desorption occurs irreversibly (i.e., r_{-6} approaches zero due to low values of $[C_6H_{12}O]$). This assumption, in addition to combination of **Equations S22** and **S26** lead to the following expression of $[C_6H_{12}O^*]$:

$$[C_6H_{12}O*] = \frac{k_5[O*]\,[C_6H_{12}]}{k_6} = \frac{k_5\frac{k_1[H_2O]\,[*]}{k_5[C_6H_{12}]+k_3[H_2O]}[C_6H_{12}]}{k_6} = \frac{k_1k_5[H_2O][C_6H_{12}][*]}{k_6(k_5[C_6H_{12}]+k_3[H_2O])} \tag{S29}$$

Rearrangement of **Equation S27** leads to the following expression for $[C_6H_{12}^*]$:

$$[C_6H_{12}*] = \frac{k_7[C_6H_{12}]\,[*]}{k_{-7}} \tag{S30}$$

The combination of **Equations S21**, **S22**, **S24**, **S28**, **S29**, and **S30** leads to the following expression for the complete site balance:

$$[L] = [*] + \frac{k_1[H_2O]}{k_2}[*] + \frac{k_1[H_2O]}{k_5[C_6H_{12}]+k_3[H_2O]}[*] + \frac{k_1k_3\,[H_2O]^2}{k_4(k_5[C_6H_{12}]+k_3[H_2O])}[*] + \frac{k_1k_5[H_2O][C_6H_{12}]}{k_6(k_5[C_6H_{12}]+k_3[H_2O])}[*] + \frac{k_7[C_6H_{12}]}{k_{-7}}[*] \tag{S31}$$

The combination of **Equations S23** and **S31** yields the complete expression for the epoxidation turnover rate:

$$\frac{r_{C_6H_{12}O}}{[L]} = \frac{\frac{k_1k_5[C_6H_{12}][H_2O]\,[*]}{k_5[C_6H_{12}]+k_3[H_2O]}}{1+\frac{k_1[H_2O]}{k_2}+\frac{k_1[H_2O]}{k_5[C_6H_{12}]+k_3[H_2O]}+\frac{k_1k_3\,[H_2O]^2}{k_4(k_5[C_6H_{12}]+k_3[H_2O])}+\frac{k_1k_5[H_2O][C_6H_{12}]}{k_6(k_5[C_6H_{12}]+k_3[H_2O])}+\frac{k_7[C_6H_{12}]}{k_{-7}}} \tag{S32}$$

Similar methods yield turnover rate expressions for the OER. The O_2 formation rate equals the rate of step 4, in which the OOH* intermediate dissociates to form O_2:

$$r_{O_2} = r_4 = k_4[OOH*] \tag{S33}$$

The combination of equations **S28** and **S33** gives:

$$r_{O_2} = \frac{k_1k_3\,[H_2O]^2[*]}{k_5[C_6H_{12}]+k_3[H_2O]} \tag{S34}$$

Combining **Equations S31** and **S34** yields an expression for the turnover rate for O_2 evolution turnover rate:

$$\frac{r_{O_2}}{[L]} = \frac{\frac{k_1k_3\,[H_2O]^2}{k_5[C_6H_{12}]+k_3[H_2O]}}{1+\frac{k_1[H_2O]}{k_2}+\frac{k_1[H_2O]}{k_5[C_6H_{12}]+k_3[H_2O]}+\frac{k_1k_3\,[H_2O]^2}{k_4(k_5[C_6H_{12}]+k_3[H_2O])}+\frac{k_1k_5[H_2O][C_6H_{12}]}{k_6(k_5[C_6H_{12}]+k_3[H_2O])}+\frac{k_7[C_6H_{12}]}{k_{-7}}} \tag{S35}$$

The ratio of $C_6H_{12}O$ formation rates to O_2 formation rates collapses to a simple form, because these reactions share a subset of steps that activate H_2O and involve reactions of the common O* intermediate:

$$\frac{r_{C_6H_{12}O}}{r_{O_2}} = \frac{k_5[C_6H_{12}]}{k_3\,[H_2O]} \tag{S36}$$

The FE_{Epox} expression relates the rate of epoxidation and O_2 evolution to the number of electrons each process produces (two for epoxidation and four for OER):

$$FE_{Epox} = \frac{2r_{C_6H_{12}O}}{2r_{C_6H_{12}O}+4r_{O_2}} = \frac{k_5[C_6H_{12}]}{k_5[C_6H_{12}]+2k_3\,[H_2O]} \tag{S37}$$

S22.2. C_6H_{12} Reaction with OH* *via* Eley-Rideal Mechanism

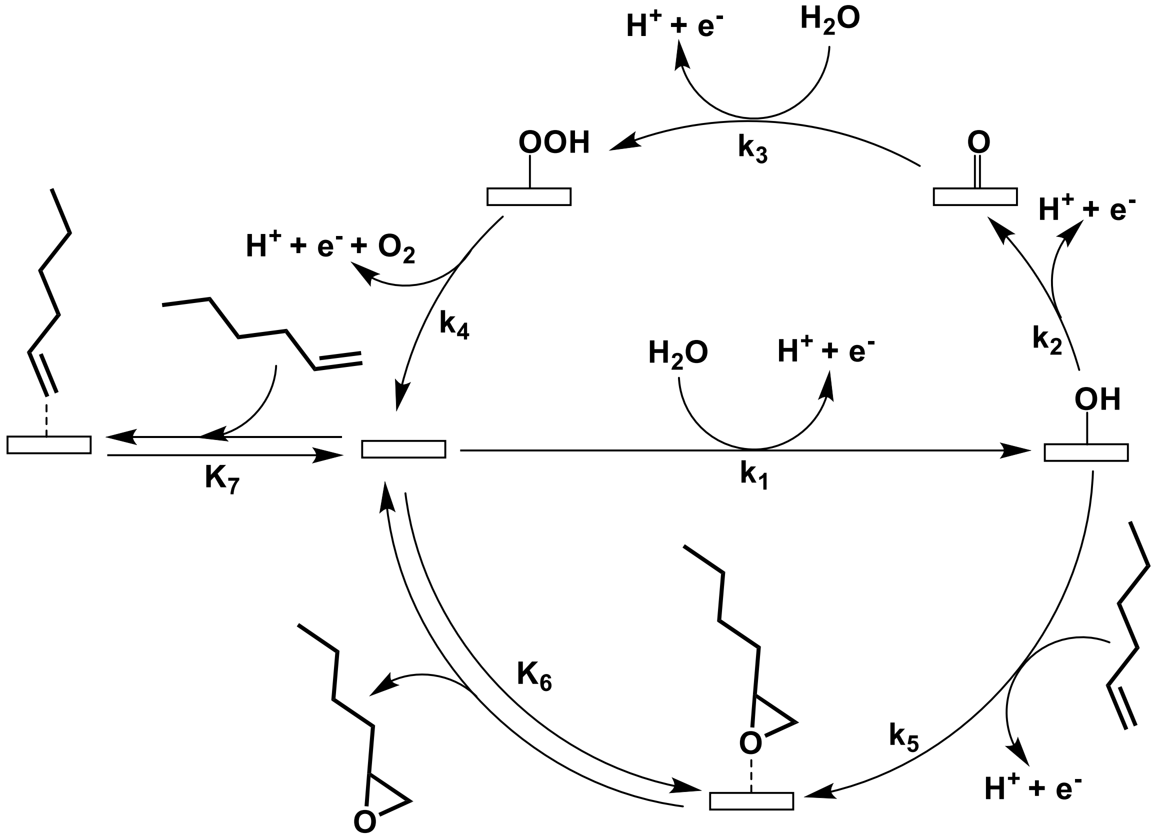

Scheme S2. Proposed catalytic cycle for C_6H_{12} epoxidation with OH* species derived from H_2O and O_2 evolution. All adsorption steps and proton-electron transfer steps are currently assumed as reversible and irreversible, respectively.

Scheme S2 presents the proposed catalytic cycles for O_2 evolution and an Eley-Rideal mechanism of C_6H_{12} epoxidation with OH* species. The epoxidation mechanism consists of steps 1, 5, 6. Here, a solvent C_6H_{12} molecule reacts with an OH* species, formed from step 1, and undergoes a proton-electron transfer to form an epoxide intermediate, denoted by $C_6H_{12}O$* (step 5). The $C_6H_{12}O$* species desorbs to form the $C_6H_{12}O$ product (step 6).

The rate expression for the formation of the epoxide product can be modeled using the net rate of step 6 and step 5, in which the OH* reacts to form $C_6H_{12}O$*:

$$r_{C_6H_{12}O} = r_6 - r_{-6} = r_5 = k_5[C_6H_{12}][O*] \quad \text{(S38)}$$

where k_i and k_{-i} are the forward and reverse rate constants for step i.

Performing a mass balance and applying PSSH on OH* yields the following expression:

$$r_{OH*} = 0 = k_1[H_2O]\,[*] - k_2[OH*] - k_5[OH*][C_6H_{12}] \quad \text{(S39)}$$

Where [*] is the number of available Au active sites that can bind and react with a species in solution and [H^+] is the concentration of protons. The following equation for [OH*] can be derived by rearranging **Equation S39**:

$$[OH\,*] = \frac{k_1[H_2O]\,[*]}{k_5[C_6H_{12}]+k_2} \quad \text{(S40)}$$

The combination of **Equations S38** and **S40** yields the following expression for $C_6H_{12}O$ formation:

$$r_{C_6H_{12}O} = \frac{k_1k_5[C_6H_{12}][H_2O]\,[*]}{k_5[C_6H_{12}]+k_2} \quad \text{(S41)}$$

An expression for [*] can be derived though a site balance of all likely surface intermediates:

$$[L] = [*] + [OH^*] + [O^*] + [OOH^*] + [C_6H_{12}O^*] + [C_6H_{12}{}^*] \quad \text{(S42)}$$

Where [L] is the total number of active sites, [O*] is the number of O* species, [OOH*] is the number of OOH* species, and [C_6H_{12}*] is the number of C_6H_{12}* species. Applying the PSSH and a mass balance to each species results in the following expressions:

$$r_{O*} = 0 = r_2 - r_3 = k_2[OH\,*] - k_3[H_2O]\,[O\,*] \quad \text{(S43)}$$

$$r_{OOH*} = 0 = r_3 - r_4 = k_3[H_2O]\,[O\,*] - k_4[OOH\,*] \quad \text{(S44)}$$

$$r_{C_6H_{12}O*} = 0 = r_5 - r_6 + r_{-6} = k_5[OH\,*]\,[C_6H_{12}] - k_6[C_6H_{12}O\,*] + k_{-6}[C_6H_{12}O] \quad \text{(S45)}$$

$$r_{C_6H_{12}*} = 0 = r_7 - r_{-7} = k_7[C_6H_{12}][*] - k_{-7}[C_6H_{12}\,*] \quad \text{(S46)}$$

Rearranging **Equation S43** and combining with **Equation S40** leads to the following expression for [O*]:

$$[O\,*] = \frac{k_2[OH*]}{k_3[H_2O]} = \frac{k_2\frac{k_1[H_2O]\,[*]}{k_5[C_6H_{12}]+k_2}}{k_3[H_2O]} = \frac{k_1k_2[*]}{k_3(k_5[C_6H_{12}]+k_2)} \quad \text{(S47)}$$

Rearranging **Equation S44** and combining with **Equation S47** leads to the following expression for [OOH*]:

$$[OOH\,*] = \frac{k_3[H_2O][O*]}{k_4} = \frac{k_3[H_2O]\frac{k_1k_2[*]}{k_3(k_5[C_6H_{12}]+k_2)}}{k_4} = \frac{k_1k_2[H_2O][*]}{k_4(k_5[C_6H_{12}]+k_2)} \quad \text{(S48)}$$

We assume that $C_6H_{12}O$ desorption occurs irreversibly (i.e., r_{-6} approaches zero due to low calues of [$C_6H_{12}O$]). This assumption, in addition to combination of **Equations S40** and **S45** lead to the following expression of [$C_6H_{12}O$]:

$$[C_6H_{12}O\,*] = \frac{k_5[OH*]\,[C_6H_{12}]}{k_6} = \frac{k_5\frac{k_1[H_2O]\,[*]}{k_5[C_6H_{12}]+k_2}[C_6H_{12}]}{k_6} = \frac{k_1k_5[C_6H_{12}][H_2O]\,[*]}{k_6(k_5[C_6H_{12}]+k_2)} \quad \text{(S49)}$$

Rearrangement of Equation **S46** leads to the following expression for [C_6H_{12}*]:

$$[C_6H_{12}\,*] = \frac{k_7[C_6H_{12}]\,[*]}{k_{-7}} \quad \text{(S50)}$$

The combination of **Equations S40**, **S42**, **S467**, **S48**, **S49**, and **S50** leads to the following expression for the complete site balance:

$$[L] = [*] + \frac{k_1[H_2O]\,[*]}{k_5[C_6H_{12}]+k_2} + \frac{k_1k_2[*]}{k_3(k_5[C_6H_{12}]+k_2)} + \frac{k_1k_2[H_2O][*]}{k_4(k_5[C_6H_{12}]+k_2)} + \frac{k_1k_5[C_6H_{12}][H_2O]\,[*]}{k_6(k_5[C_6H_{12}]+k_2)} + \frac{k_7[C_6H_{12}]\,[*]}{k_{-7}} \quad \text{(S51)}$$

Combining **Equations S41** and **S51** yields an expression for the epoxidation turnover rate:

$$\frac{r_{C_6H_{12}O}}{[L]} = \frac{\frac{k_1k_5[C_6H_{12}][H_2O]}{k_5[C_6H_{12}]+k_2}}{1+\frac{k_1[H_2O]}{k_5[C_6H_{12}]+k_2}+\frac{k_1k_2}{k_3(k_5[C_6H_{12}]+k_2)}+\frac{k_1k_2[H_2O]}{k_4(k_5[C_6H_{12}]+k_2)}+\frac{k_1k_5[C_6H_{12}][H_2O]}{k_6(k_5[C_6H_{12}]+k_2)}+\frac{k_7[C_6H_{12}]}{k_{-7}}} \quad \text{(S52)}$$

After substituting in terms for [OOH*] and using the site balance for [L], the O_2 formation rate can be modeled as follows:

$$r_{O_2} = r_4 = k_4[OOH\,*] = \frac{\frac{k_1k_2[H_2O]}{k_5[C_6H_{12}]+k_2}}{1+\frac{k_1[H_2O]}{k_5[C_6H_{12}]+k_2}+\frac{k_1k_2}{k_3(k_5[C_6H_{12}]+k_2)}+\frac{k_1k_2[H_2O]}{k_4(k_5[C_6H_{12}]+k_2)}+\frac{k_1k_5[C_6H_{12}][H_2O]}{k_6(k_5[C_6H_{12}]+k_2)}+\frac{k_7[C_6H_{12}]}{k_{-7}}} \quad \text{(S53)}$$

Thus, the rate ratio of $C_6H_{12}O$ formation to O_2 formation and epoxidation Faradaic efficiency expressions are:

$$\frac{r_{C_6H_{12}O}}{r_{O_2}} = \frac{k_5[C_6H_{12}]}{k_2} \quad \text{(S54)}$$

$$FE_{Epox} = \frac{2r_{C_6H_{12}O}}{2r_{C_6H_{12}O}+4r_{O_2}} = \frac{k_5[C_6H_{12}]}{k_5[C_6H_{12}]+2k_2} \quad \text{(S55)}$$

S22.3. C_6H_{12} Reaction with OOH* *via* Eley-Rideal Mechanism

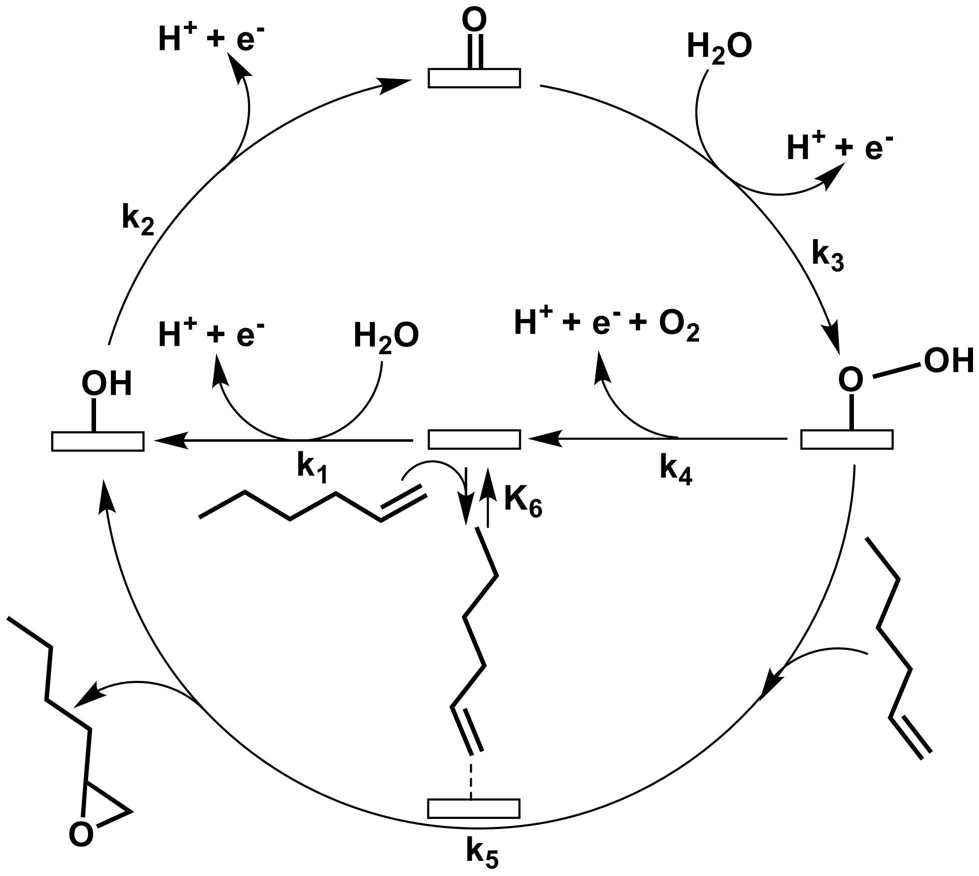

Scheme S3. Proposed catalytic cycle for C_6H_{12} epoxidation with OOH* species derived from H_2O and O_2 evolution. All adsorption steps and proton-electron transfer steps are currently assumed as reversible and irreversible, respectively.

Scheme S3 presents the proposed catalytic cycles for O_2 evolution and an Eley-Rideal mechanism of C_6H_{12} epoxidation with OOH* species. The epoxidation mechanism consists of steps 2, 3, and 5. The mechanism begins and ends with an OH* species rather than an empty site to maintain that the epoxidation reaction only results in the transfer of two electrons. As in the O_2 evolution reaction, the OH* species forms an O* species through a proton-electron transfer step (step 2) and this O* species then reacts with a solvent H_2O molecule to form an OOH* species (step 3). A solvent C_6H_{12} molecule then reacts with the OOH* species and produces the $C_6H_{12}O$ product in one step, returning to the OH* species (step 5). Note that the reversible C_6H_{12} adsorption step is denoted as step 6 in this mechanism.

The rate expression for the formation of the epoxide product can be modeled with the rate of step 5, in which a solvent C_6H_{12} molecule reacts with an OOH* species to form $C_6H_{12}O$:

$$r_{C_6H_{12}O} = r_5 = k_5[OOH*]\,[C_6H_{12}] \quad \text{(S56)}$$

Applying a site balance and PSSH on the OOH* intermediate leads to the following expression:

$$r_{OOH*} = 0 = r_3 - r_4 - r_5 = k_3[O*][H_2O] - - k_4[OOH*] - k_5[OOH*]\,[C_6H_{12}] \quad \text{(S57)}$$

Which can be rearranged to:

$$[OOH*] = \frac{k_3[O*][H_2O]}{k_5[C_6H_{12}] + k_4} \quad \text{(S58)}$$

To derive an expression for [O*], we apply a site balance and PSSH to both the [O*] and [OH*] species:

$$r_{O*} = 0 = r_2 - r_3 = k_2[OH*] - k_3[O*]\,[H_2O] \quad \text{(S59)}$$

$$r_{OH*} = 0 = r_1 - r_2 - r_5 = -k_2[OH*] + k_5[OOH*]\,[C_6H_{12}] + k_1[*]\,[H_2O] \quad \text{(S60)}$$

Equation S60 contains [OOH*]. In order to derive an expression for [OH*] that does not include [OOH*], which would not allow us to obtain an expression for surface intermediates in terms of rate constants and liquid-phase species, we rearrange **Equation S59** to obtain the following expression for [O*]:

$$[O*] = \frac{k_2[OH*]}{k_3[H_2O]} \quad \text{(S61)}$$

The combination of **Equations S58** and **S61** leads to the following:

$$[OOH*] = \frac{k_3[O*][H_2O]}{k_5[C_6H_{12}] + k_4} = \frac{k_3\frac{k_2[OH*]}{k_3[H_2O]}[H_2O]}{k_5[C_6H_{12}] + k_4} = \frac{k_2[OH*]}{k_5[C_6H_{12}] + k_4} \quad \text{(S62)}$$

Substituting **Equation S62** into **Equation S60** results in:

$$r_{OH*} = 0 = r_1 - r_2 - r_5 = k_1[*]\,[H_2O] - k_2[OH*] + k_5[OOH*]\,[C_6H_{12}] = k_1[*]\,[H_2O] - k_2[OH*] + \frac{k_2k_5[OH*]}{k_5[C_6H_{12}] + k_4} \quad \text{(S63)}$$

Equation S63 rearranges to give an expression for [OH*]:

$$[OH*] = \frac{k_1[H_2O][*]}{k_2 - \frac{k_2k_5[C_6H_{12}]}{k_5[C_6H_{12}] + k_4}} \quad \text{(S64)}$$

Combination of **Equations S61** and **S64** results in the following expression for [O*]:

$$[O*] = \frac{k_2[OH*]}{k_3[H_2O]} = \frac{k_2\frac{k_1[H_2O][*]}{k_2 - \frac{k_2k_5[C_6H_{12}]}{k_5[C_6H_{12}] + k_4}}}{k_3[H_2O]} = \frac{k_1k_2[*]}{k_3\left(k_2 - \frac{k_2k_5[C_6H_{12}]}{k_5[C_6H_{12}] + k_4}\right)} \quad \text{(S65)}$$

Substitution of **Equation S65** into **Equation S58** leads to the following [OOH*] expression:

$$[OOH *] = \frac{k_3[O*][H_2O]}{k_5[C_6H_{12}]+ k_4} = \frac{k_3\frac{k_2[OH*]}{k_3[H_2O]}[H_2O]}{k_5[C_6H_{12}]+ k_4} = \frac{k_2[OH*]}{k_5[C_6H_{12}]+ k_4} = \frac{k_2\frac{k_1[H_2O][*]}{k_2-\frac{k_2k_5[C_6H_{12}]}{k_5[C_6H_{12}]+ k_4}}}{k_5[C_6H_{12}]+ k_4} = \frac{k_1k_2[H_2O][*]}{(k_5[C_6H_{12}]+ k_4)\left(k_2-\frac{k_2k_5[C_6H_{12}]}{k_5[C_6H_{12}]+ k_4}\right)} \quad \text{(S66)}$$

The combination of **Equations S56** and **S66** lead to the following expression for $C_6H_{12}O$ formation:

$$r_{C_6H_{12}O} = k_5[OOH *]\,[C_6H_{12}] = \frac{k_1k_2k_5[C_6H_{12}][H_2O][*]}{(k_5[C_6H_{12}]+ k_4)\left(k_2-\frac{k_2k_5[C_6H_{12}]}{k_5[C_6H_{12}]+ k_4}\right)} \quad \text{(S67)}$$

The total number of active sites ([L]) can be written as:

$$[L] = [*] + [OH^*] + [O^*] + [OOH^*] + [C_6H_{12}{}^*] \quad \text{(S68)}$$

An expression for $[C_6H_{12}*]$ can be found with a site balance and application of the PSSH on C6H12*:

$$r_{C_6H_{12}*} = 0 = r_6 - r_{-6} = k_6[C_6H_{12}][*] - k_{-6}[C_6H_{12}\,*] \quad \text{(S69)}$$

Rearrangement of **Equation S69** leads to the following expression for $[C_6H_{12}*]$:

$$[C_6H_{12}\,*] = \frac{k_6[C_6H_{12}]\,[*]}{k_{-6}} \quad \text{(S70)}$$

The combination of **Equations S64**, **S65**, **S66**, **S68**, and **S69** leads to the following expression for the complete site balance:

$$[L] = [*] + \frac{k_1[H_2O][*]}{k_2-\frac{k_2k_5[C_6H_{12}]}{k_5[C_6H_{12}]+ k_4}} + \frac{k_1k_2[*]}{k_3\left(k_2-\frac{k_2k_5[C_6H_{12}]}{k_5[C_6H_{12}]+ k_4}\right)} + \frac{k_1k_2[H_2O][*]}{(k_5[C_6H_{12}]+ k_4)\left(k_2-\frac{k_2k_5[C_6H_{12}]}{k_5[C_6H_{12}]+ k_4}\right)} + \frac{k_6[C_6H_{12}]\,[*]}{k_{-6}} \quad \text{(S71)}$$

Combining **Equations S67** and **S71** yields an expression for the epoxidation turnover rate:

$$\frac{r_{C_6H_{12}O}}{[L]} = \frac{\frac{k_1k_2k_5[C_6H_{12}][H_2O]}{(k_5[C_6H_{12}]+ k_4)\left(k_2-\frac{k_2k_5[C_6H_{12}]}{k_5[C_6H_{12}]+ k_4}\right)}}{1+\frac{k_1[H_2O]}{k_2-\frac{k_2k_5[C_6H_{12}]}{k_5[C_6H_{12}]+ k_4}}+\frac{k_1k_2}{k_3\left(k_2-\frac{k_2k_5[C_6H_{12}]}{k_5[C_6H_{12}]+ k_4}\right)}+\frac{k_1k_2[H_2O]}{(k_5[C_6H_{12}]+ k_4)\left(k_2-\frac{k_2k_5[C_6H_{12}]}{k_5[C_6H_{12}]+ k_4}\right)}+\frac{k_6[C_6H_{12}]}{k_{-6}}} \quad \text{(S72)}$$

After substituting in terms for [OOH*] and using the site balance for [L], the O_2 formation rate can be modeled as follows:

$$r_{O_2} = r_4 = k_4[OOH*] = \frac{\frac{k_1 k_2 k_4 [H_2O]}{k_3\left(k_2 - \frac{k_2 k_5 [C_6H_{12}]}{k_5[C_6H_{12}] + k_4}\right)}}{1 + \frac{k_1[H_2O]}{k_2 - \frac{k_2 k_5 [C_6H_{12}]}{k_5[C_6H_{12}] + k_4}} + \frac{k_1 k_2}{k_3\left(k_2 - \frac{k_2 k_5 [C_6H_{12}]}{k_5[C_6H_{12}] + k_4}\right)} + \frac{k_1 k_2 [H_2O]}{(k_5[C_6H_{12}] + k_4)\left(k_2 - \frac{k_2 k_5 [C_6H_{12}]}{k_5[C_6H_{12}] + k_4}\right)} + \frac{k_6[C_6H_{12}]}{k_{-6}}} \qquad (S73)$$

Thus, the rate ratio of $C_6H_{12}O$ formation to O_2 formation and epoxidation Faradaic efficiency expressions are:

$$\frac{r_{C_6H_{12}O}}{r_{O_2}} = \frac{k_5[C_6H_{12}]}{k_4} \qquad (S74)$$

$$FE_{Epox} = \frac{2r_{C_6H_{12}O}}{2r_{C_6H_{12}O} + 4r_{O_2}} = \frac{k_5[C_6H_{12}]}{k_5[C_6H_{12}] + 2k_4} \qquad (S75)$$

S22.4. C_6H_{12} Reaction with O_2* *via* Eley-Rideal Mechanism

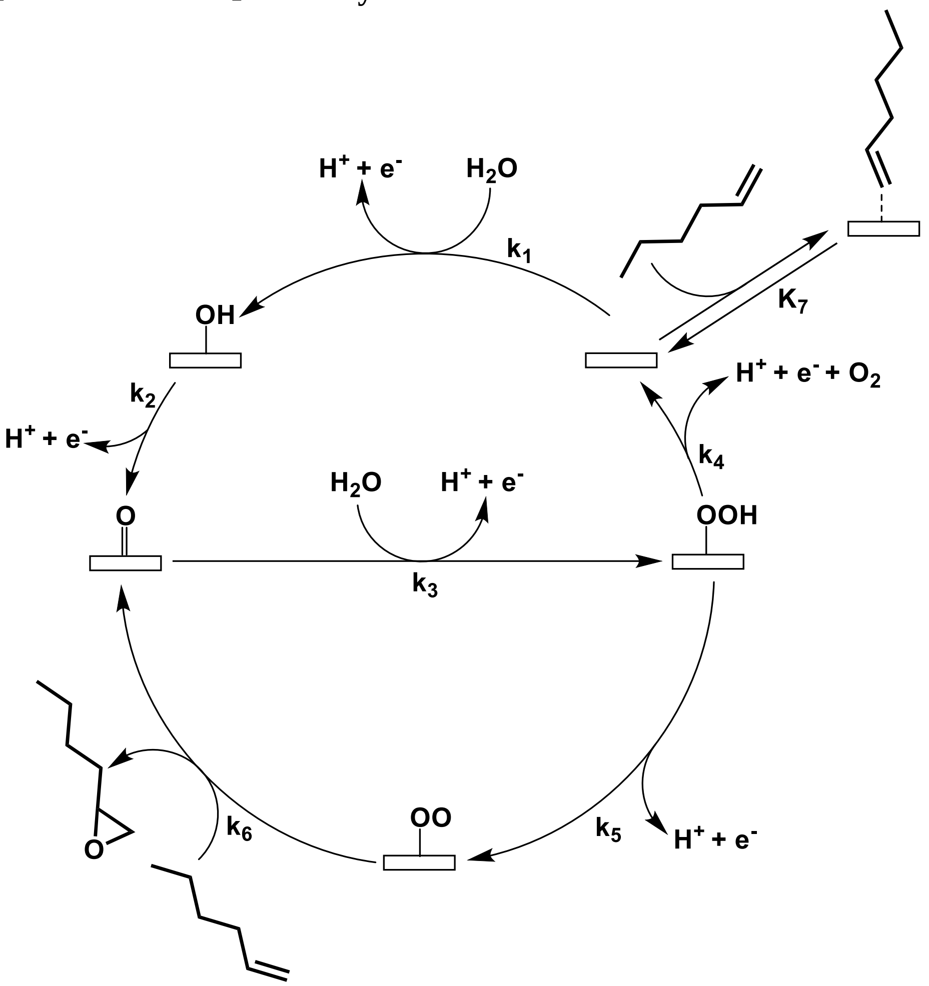

Scheme S4. Proposed catalytic cycle for C_6H_{12} epoxidation with O_2* species derived from H_2O and O_2 evolution. All adsorption steps and proton-electron transfer steps are currently assumed as reversible and irreversible, respectively.

Scheme S4 presents the proposed catalytic cycles for O_2 evolution and an Eley-Rideal mechanism of C_6H_{12} epoxidation with O_2* species. The epoxidation mechanism consists of steps 3, 5, and 6. The mechanism begins and ends with an O* species rather than an empty site to maintain that the epoxidation reaction only results in the transfer of two electrons. As in the O_2 evolution reaction, the O* species forms an OOH* species through reaction with a solvent H_2O molecule (step 3). This OOH* species undergoes a proton-electron transfer to form an O_2* species (step 5), which then reacts with a solvent C_6H_{12} molecule to produce the $C_6H_{12}O$ product in one step, returning to the O* species (step 6).

The rate expression for the formation of the epoxide product can be modeled with the rate of step 6, in which a solvent C_6H_{12} molecule reacts with an O_2* species to form $C_6H_{12}O$:

$$r_{C_6H_{12}O} = r_6 = k_6[O_2 *]\,[C_6H_{12}] \qquad \text{(S76)}$$

Applying a site balance and PSSH on the O_2* intermediate leads to the following expression:

$$r_{OO*} = 0 = r_5 - r_6 = k_5[OOH *] - k_6[O_2 *]\,[C_6H_{12}] \tag{S77}$$

Rearrangement of **Equation S77** leads to the following expression for [OO*]:

$$[O_2 *] = \frac{k_5[OOH*]}{k_6[C_6H_{12}]} \tag{S78}$$

To derive an expression for [O_2*], we apply a site balance and PSSH on the [OOH*], [O*], and [OH*] species:

$$r_{OOH*} = 0 = r_3 - r_4 - r_5 = k_3[H_2O]\,[O *] - k_4[OOH *] - k_5[OOH *] \tag{S79}$$

$$r_{O*} = 0 = r_2 - r_3 + r_6 = k_2[OH *] - k_3[H_2O]\,[O *] + k_6[C_6H_{12}][O_2 *] \tag{S80}$$

$$r_{OH*} = 0 = r_1 - r_2 = k_1[H_2O]\,[*] - k_2\,[OH *] \tag{S81}$$

Rearrangement of **Equation S79** leads to the following expression for [OH*]:

$$[OH *] = \frac{k_1[H_2O][*]}{k_2[H_2O]} = \frac{k_1}{k_2}[*] \tag{S82}$$

Equation S80 contains [O_2*]. In order to derive an expression for [O*] that does not include [O_2*], which would not allow us to obtain an expression for surface intermediates in terms of rate constants and liquid-phase species, we rearrange **Equation S79** to obtain the following expression for [OOH*]:

$$[OOH *] = \frac{k_3[O*]\,[H_2O]}{k_5+k_4} \tag{S83}$$

Combination of **Equation S83** with **Equation S78** results in the following expression for [O_2*]:

$$[O_2 *] = \frac{k_5[OOH*]}{k_6[C_6H_{12}]} = \frac{k_5\frac{k_3[O*]\,[H_2O]}{k_5+k_4}}{k_6[C_6H_{12}]} = \frac{k_3k_5[O*]\,[H_2O]}{k_6(k_5+k_4)[C_6H_{12}]} \tag{S84}$$

Substitution of **Equation S84** into **Equation S80** leads to:

$$r_{O*} = 0 = r_2 - r_3 + r_6 = k_2[OH *] - k_3[H_2O]\,[O *] + k_6[C_6H_{12}][O_2 *] = k_2[OH *] - k_3[H_2O]\,[O *] + k_6[C_6H_{12}]\frac{k_3k_5[O*]\,[H_2O]}{k_6(k_5+k_4)[C_6H_{12}]} = k_2[OH *] - k_3[H_2O]\,[O *] + \frac{k_3k_5[O*]\,[H_2O]}{k_5+k_4} \tag{S85}$$

Rearrangement of **Equation S85** and combination with **Equation S82** gives an expression for [O*]:

$$[O *] = \frac{k_2[OH*]}{k_3[H_2O] - \frac{k_3k_5[H_2O]}{k_5+k_4}} = \frac{k_2\frac{k_1}{k_2}[*]}{k_3[H_2O] - \frac{k_3k_5[H_2O]}{k_5+k_4}} = \frac{k_1[*]}{[H_2O]\left(k_3 - \frac{k_3k_5}{k_5+k_4}\right)} \tag{S86}$$

Combination of **Equations S83** and **S86** leads to the following expression for [OOH*]:

$$[OOH\ *] = \frac{k_3[O*]\ [H_2O]}{k_5+k_4} = \frac{k_3\frac{k_1[*]}{[H_2O]\left(k_3-\frac{k_3k_5}{k_5+k_4}\right)}\ [H_2O]}{k_5+k_4} = \frac{k_1k_3[*]}{(k_5+k_4)\left(k_3-\frac{k_3k_5}{k_5+k_4}\right)} \tag{S87}$$

Equations S84 and **S87** combine to form an expression for [O_2*]:

$$[O_2\ *] = \frac{k_5[OOH*]}{k_6[C_6H_{12}]} = \frac{k_5\frac{k_1k_3[*]}{(k_5+k_4)\left(k_3-\frac{k_3k_5}{k_5+k_4}\right)}}{k_6[C_6H_{12}]} = \frac{k_1k_3k_5[*]}{k_6[C_6H_{12}](k_5+k_4)\left(k_3-\frac{k_3k_5}{k_5+k_4}\right)} \tag{S88}$$

The combination of **Equations S76** and **S88** lead to the following expression for $C_6H_{12}O$ formation:

$$r_{C_6H_{12}O} = r_6 = k_6[O_2\ *]\ [C_6H_{12}] = k_6\frac{k_1k_3k_5[*]}{k_6[C_6H_{12}](k_5+k_4)\left(k_3-\frac{k_3k_5}{k_5+k_4}\right)}\ [C_6H_{12}] = \frac{k_1k_3k_5[*]}{k_6[C_6H_{12}](k_5+k_4)\left(k_3-\frac{k_3k_5}{k_5+k_4}\right)} \tag{S89}$$

The total number of active sites ([L]) can be written as:

$$[L] = [*] + [OH^*] + [O^*] + [OOH^*] + [O_2{}^*] + [C_6H_{12}{}^*] \tag{S90}$$

An expression for [C_6H_{12}*] can be found with a site balance and application of the PSSH on C_6H12*:

$$r_{C_6H_{12}*} = 0 = r_7 - r_{-7} = k_7[C_6H_{12}][*] - k_{-7}[C_6H_{12}\ *] \tag{S91}$$

Rearrangement of **Equation S91** leads to the following expression for [C_6H_{12}*]:

$$[C_6H_{12}\ *] = \frac{k_7[C_6H_{12}]\ [*]}{k_{-7}} \tag{S92}$$

The combination of **Equations S82**, **S86**, **S87**, **S88**, **S90**, and **S9** leads to the following expression for the complete site balance:

$$[L] = [*] + \frac{k_1[*]}{k_2} + \frac{k_1[*]}{[H_2O]\left(k_3-\frac{k_3k_5}{k_5+k_4}\right)} + \frac{k_1k_3[*]}{(k_5+k_4)\left(k_3-\frac{k_3k_5}{k_5+k_4}\right)} + \frac{k_1k_3k_5[*]}{k_6[C_6H_{12}](k_5+k_4)\left(k_3-\frac{k_3k_5}{k_5+k_4}\right)} + \frac{k_7[C_6H_{12}]\ [*]}{k_{-7}} \tag{S93}$$

Combining **Equations S89** and **S93** yields an expression for the epoxidation turnover rate:

$$\frac{r_{C_6H_{12}O}}{[L]} = \frac{\frac{k_1 k_3 k_5 [*]}{k_6[C_6H_{12}](k_5+k_4)\left(k_3-\frac{k_3k_5}{k_5+k_4}\right)}}{1+\frac{k_1}{k_2}+\frac{k_1}{[H_2O]\left(k_3-\frac{k_3k_5}{k_5+k_4}\right)}+\frac{k_1k_3}{(k_5+k_4)\left(k_3-\frac{k_3k_5}{k_5+k_4}\right)}+\frac{k_1k_3k_5}{k_6[C_6H_{12}](k_5+k_4)\left(k_3-\frac{k_3k_5}{k_5+k_4}\right)}+\frac{k_7[C_6H_{12}]}{k_{-7}}}$$

(S94)

After substituting in terms for [OOH*] and using the site balance for [L], the O_2 formation rate can be modeled as follows:

$$r_{O_2} = r_4 = k_4[OOH *] = \frac{\frac{k_1 k_3 k_4 [*]}{k_6[C_6H_{12}](k_5+k_4)\left(k_3-\frac{k_3k_5}{k_5+k_4}\right)}}{1+\frac{k_1}{k_2}+\frac{k_1}{[H_2O]\left(k_3-\frac{k_3k_5}{k_5+k_4}\right)}+\frac{k_1k_3}{(k_5+k_4)\left(k_3-\frac{k_3k_5}{k_5+k_4}\right)}+\frac{k_1k_3k_5}{k_6[C_6H_{12}](k_5+k_4)\left(k_3-\frac{k_3k_5}{k_5+k_4}\right)}+\frac{k_7[C_6H_{12}]}{k_{-7}}} \quad \text{(S95)}$$

Thus, the rate ratio of $C_6H_{12}O$ formation to O_2 formation and epoxidation Faradaic efficiency expressions are:

$$\frac{r_{C_6H_{12}O}}{r_{O_2}} = \frac{k_5}{k_4} \quad \text{(S96)}$$

$$FE_{Epox} = \frac{2r_{C_6H_{12}O}}{2r_{C_6H_{12}O}+4r_{O_2}} = \frac{k_5}{k_5+2k_4} \quad \text{(S97)}$$

S22.5. C_6H_{12} Reaction with O* via Langmuir-Hinshelwood Mechanism

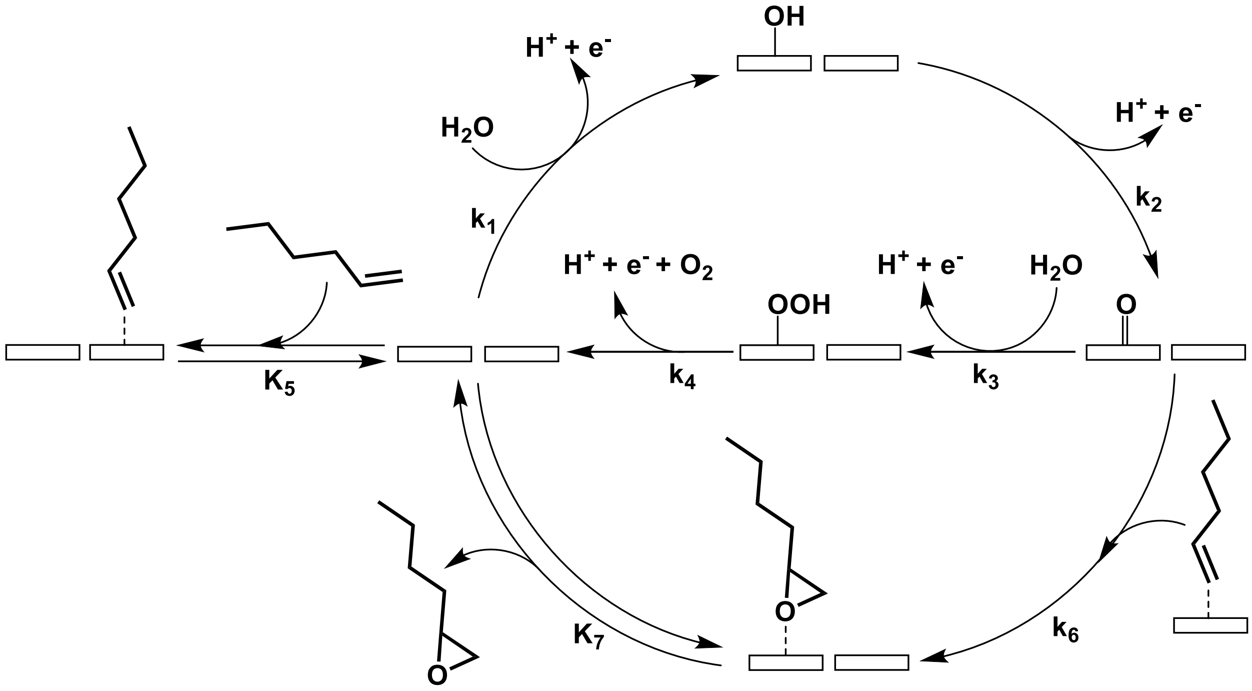

Scheme S5. Proposed catalytic cycle for C_6H_{12} epoxidation with O* species derived from H_2O following a Langmuir-Hinshelwood mechanism and O_2 evolution. All adsorption steps and proton-electron transfer steps are currently assumed as reversible and irreversible, respectively.

Scheme S5 presents the proposed catalytic cycles for O_2 evolution and a Langmuir-Hinshelwood mechanism of C_6H_{12} epoxidation with O* species. All empty sites are assumed to be identical and allow for the binding of either C_6H_{12} or H_2O. The epoxidation mechanism consists of steps 1, 2, 5, 6, and 7. The O* species forms following the same steps as described for the O_2 evolution reaction mechanism (steps 1 and 2). A solvent C_6H_{12} molecule molecularly adsorbs to form a C_6H_{12}* species (step 5). This C_6H_{12}* species and O* species react to form the epoxide intermediate $C_6H_{12}O$* (step 6), which then desorbs to form the $C_6H_{12}O$ product (step 7).

The rate expression for the formation of the epoxide product corresponds to the net rate of steps 7 and 6, in which C_6H_{12}* and O* react to form $C_6H_{12}O$*:

$$r_{C_6H_{12}O} = r_7 - r_{-7} = r_6 = k_6[C_6H_{12}\ *][O\ *] \tag{S98}$$

A site balance and PSSH to the to the C_6H_{12}* and O* intermediates result in the following expressions:

$$r_{C_6H_{12}*} = 0 = r_5 - r_{-5} - r_6 = k_5[C_6H_{12}][*]^2 - k_{-5}[C_6H_{12}\ *][*] - k_6[C_6H_{12}\ *][O\ *] \tag{S99}$$

$$r_{O*} = 0 = r_2 - r_3 - r_6 = k_2[OH\ *][*] - k_3[H_2O]\ [O\ *][*] - k_6[C_6H_{12}\ *][O\ *] \tag{S100}$$

Equation S99 rearranges to the following:

$$[C_6H_{12}*] = \frac{k_5[C_6H_{12}][*]^2}{k_{-5}[*] + k_6[O*]} \quad \text{(S101)}$$

A site balance and PSSH on the [OH*] species lead to:

$$r_{OH*} = 0 = r_1 - r_2 = k_1[H_2O]\,[*]^2 - k_2\,[OH*][*] \quad \text{(S102)}$$

Rearrangement of **Equation S102** leads to the following expression for [OH*]:

$$[OH*] = \frac{k_1[H_2O][*]}{k_2} \quad \text{(S103)}$$

Substitution of **Equations S101** and **S103** into **Equation S100** and then algebraic rearrangement leads to the following expression for r_{O*}:

$$r_{O*} = 0 = r_2 - r_3 - r_6 = k_2[OH*][*] - k_3[H_2O]\,[O*][*] - k_6[C_6H_{12}*][O*]$$
$$= k_1[H_2O][*]^2 - k_3[H_2O]\,[O*][*] - k_6[C_6H_{12}*][O*]$$
$$= k_1[H_2O][*]^2 - k_3[H_2O]\,[O*][*] - k_6\frac{k_5[C_6H_{12}][*]^2}{k_{-5}[*] + k_6[O*]}[O*]$$
$$= k_1k_{-5}[H_2O][*]^3 + k_1k_6[H_2O][O*][*]^2 - k_3k_{-5}[H_2O]\,[O*][*]^2 - k_3k_6[H_2O]\,[O*]^2[*] - k_5k_6[C_6H_{12}][O*][*]^2$$
$$= -k_3k_6[H_2O]\,[O*]^2 + [O*](k_1k_6[H_2O][*] - k_3k_{-5}[H_2O][*] - k_5k_6[C_6H_{12}][*]) + k_1k_{-5}[H_2O][*]^2 \quad \text{(S104)}$$

An expression for [O*] can be derived by solving the quadratic equation in **Equation S104**:

$$[O*] = \frac{-(k_1k_6[H_2O][*]-k_3k_{-5}[H_2O][*]-k_5k_6[C_6H_{12}][*]) \pm \sqrt{(k_1k_6[H_2O][*]-k_3k_{-5}[H_2O][*]-k_5k_6[C_6H_{12}][*])^2 + k_1k_3k_{-5}k_6[H_2O]^2[*]^2}}{-2k_3k_6[H_2O]} \quad \text{(S105)}$$

Only the negative root of **Equation S105** will lead to a value of [O*] that makes physical sense. [O*] can then be defined as:

$$[O*] = \beta[*] \quad \text{(S106)}$$

Where β represents the group of rate constants and reactant concentrations described below:

$$\beta = \frac{(k_1k_6[H_2O]-k_3k_{-5}[H_2O]-k_5k_6[C_6H_{12}]) + \sqrt{(k_1k_6[H_2O]-k_3k_{-5}[H_2O]-k_5k_6[C_6H_{12}])^2 + k_1k_3k_{-5}k_6[H_2O]^2}}{-2k_3k_6[H_2O]} \quad \text{(S107)}$$

Combination of **Equations S101** and **S106** leads to an expression for [C_6H_{12}*]:

$$[C_6H_{12}*] = \frac{k_5[C_6H_{12}][*]^2}{k_{-5}[*]+ k_6[O*]} = \frac{k_5[C_6H_{12}][*]^2}{k_{-5}[*]+ k_6[*]\beta} = \frac{k_5[C_6H_{12}][*]}{k_{-5}+ k_6\beta} \quad \text{(S108)}$$

The combination of **Equations S98**, **S106**, and **S108** lead to the following expression for $C_6H_{12}O$ formation:

$$r_{C_6H_{12}O} = r_7 - r_{-7} = r_6 = k_6[C_6H_{12}*][O*] = \frac{\beta k_5 k_6[C_6H_{12}][*]^2}{(k_{-5}+ k_6\beta)[L]} \quad \text{(S109)}$$

The total number of active sites ([L]) can be written as:

$$[L] = [*] + [OH^*] + [O^*] + [OOH^*] + [C_6H_{12}{}^*] + [C_6H_{12}O^*] \quad \text{(S110)}$$

Expressions for [OOH*] and [$C_6H_{12}O$*] come from their site balance with applied PSSH:

$$r_{OOH*} = 0 = r_3 - r_4 = k_3[O*]\,[H_2O]\,[*] - k_4[OOH*][*] \quad \text{(S111)}$$

$$r_{C_6H_{12}O*} = 0 = r_6 - r_7 + r_{-7} = k_6[C_6H_{12}*][O*] - k_7[C_6H_{12}O*][*] + k_{-7}[C_6H_{12}O][*]^2 \quad \text{(S112)}$$

Rearrangement of **Equation S111** and combination with **Equation S106** leads to the following expression for [OOH*]:

$$[OOH*] = \frac{k_3[O*]\,[H_2O][*]}{k_4[*]} = \frac{\beta k_3\,[H_2O][*]}{k_4} \quad \text{(S113)}$$

We assume that $C_6H_{12}O$ desorption occurs irreversibly (i.e., r_{-7} approaches zero due to low values of [$C_6H_{12}O$]). This assumption, in addition to combination of **Equations S108** and **S112** lead to the following expression of [$C_6H_{12}O$*]:

$$[C_6H_{12}O*] = \frac{k_6[C_6H_{12}*][O*]}{k_7[*]} = \frac{k_6\frac{k_5[C_6H_{12}][*]}{k_{-5}+ k_6\beta}\beta[*]}{k_7[*]} = \frac{\beta k_5 k_6[C_6H_{12}][*]}{k_7(k_{-5}+ k_6\beta)} \quad \text{(S114)}$$

The combination of **Equations S103**, **S106**, **S108**, **S110**, **S113**, and **S114** leads to the following expression for the complete site balance:

$$[L] = [*] + \frac{k_1[H_2O][*]}{k_2} + \beta[*] + \frac{\beta k_3\,[H_2O][*]}{k_4} + \frac{k_5[C_6H_{12}][*]}{k_{-5}+ k_6\beta} + \frac{\beta k_5 k_6[C_6H_{12}][*]}{k_7(k_{-5}+ k_6\beta)} \quad \text{(S115)}$$

Combining **Equations S109** and **S115** yields an expression for the epoxidation turnover rate:

$$\frac{r_{C_6H_{12}O}}{[L]} = \frac{\frac{\beta k_5 k_6[C_6H_{12}]}{k_{-5}+ k_6\beta}}{1 + \frac{k_1[H_2O]}{k_2} + \beta + \frac{\beta k_3\,[H_2O]}{k_4} + \frac{k_5[C_6H_{12}]}{k_{-5}+ k_6\beta} + \frac{\beta k_5 k_6[C_6H_{12}]}{k_7\left(k_{-5}+ k_6\beta\right)}} \quad \text{(S116)}$$

After substituting in terms for [OOH*] and using the site balance for [L], the O_2 formation rate can be modeled as follows:

$$r_{O_2} = r_4 = k_4[OOH*] = \frac{\beta k_3\,[H_2O]}{1 + \frac{k_1[H_2O]}{k_2} + \beta + \frac{\beta k_3\,[H_2O]}{k_4} + \frac{k_5[C_6H_{12}]}{k_{-5} + k_6\beta} + \frac{\beta k_5 k_6[C_6H_{12}]}{k_7(k_{-5} + k_6\beta)}} \quad \text{(S117)}$$

Thus, the rate ratio of $C_6H_{12}O$ formation to O_2 formation and epoxidation Faradaic efficiency expressions are:

$$\frac{r_{C_6H_{12}O}}{r_{O_2}} = \frac{\frac{k_5k_6[C_6H_{12}]}{k_{-5} + k_6\beta}}{k_3[H_2O]} \quad \text{(S118)}$$

$$FE_{Epox} = \frac{2r_{C_6H_{12}O}}{2r_{C_6H_{12}O} + 4r_{O_2}} = \frac{\frac{k_5k_6[C_6H_{12}]}{k_{-5} + k_6\beta}}{\frac{k_5k_6[C_6H_{12}]}{k_{-5} + k_6\beta} + 2k_3[H_2O]} \quad \text{(S119)}$$

S23. Rate Ratio and Epoxidation Faradaic Efficiency Dependencies

S23.1. Rate Ratio Dependency on Ratio of [C_6H_{12}] to [H_2O]

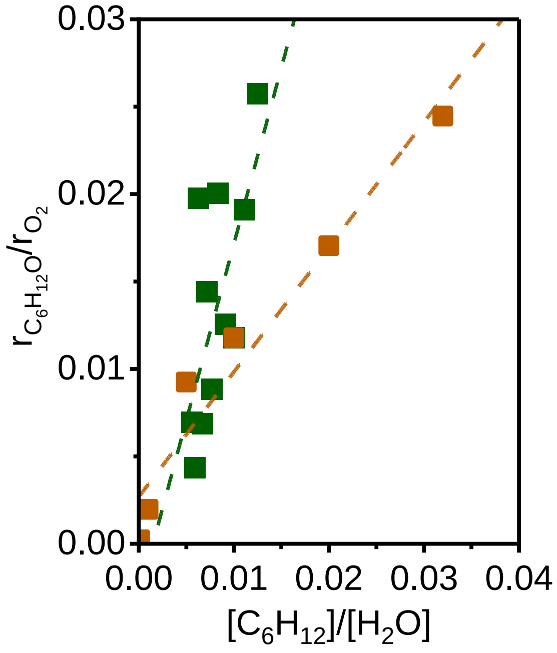

Figure S34. Epoxidation and O_2 evolution rate ratios (black) as a function of [C_6H_{12}]/[H_2O] (1.31 $V_{Fc/Fc+}$, 0.1 M TBAClO$_4$, CH_3CN). Green and orange squares correspond to data from measurements with varying [C_6H_{12}] (**Figure 5a**) and [H_2O] (**Figure 5b**), respectively. Orange dash and green dash lines reflect the linear fit of measurements with varying [C_6H_{12}] and measurements with varying [H_2O], respectively.

Figure S34 shows that the $\frac{r_{C_6H_{12}O}}{r_{O_2}}$ depends linearly on $\frac{[C_6H_{12}]}{[H_2O]}$. This linear relationship arises only for the Eley-Rideal and Langmuir-Hinshelwood mechanisms with a reactive O* species. The linear dependence of $\frac{r_{C_6H_{12}O}}{r_{O_2}}$ on $\frac{[C_6H_{12}]}{[H_2O]}$ excludes the mechanisms that involve a reactive OH*, OOH*, or O_2* species.

The respective linear fits of the data from varying [C_6H_{12}] or [H_2O] have different slopes and intercepts. The fit of the data from the varying [C_6H_{12}] measurements has a slope of 0.7±0.1 and intercept of 0.002±0.001. The fit of the data from the varying [H_2O] measurements has a slope of 2.0±0.7 and intercept of -0.003±0.006. The difference in slopes between the two data sets indicate that the ratio of epoxidation to O_2 evolution rate constants (k_5/k_3) responds sensitively to the reaction microenvironment in a way that cannot be captured by only the ratio of [C_6H_{12}] to [H_2O]. C_6H_{12} and H_2O will likely not interact with or stabilize surface species and epoxidation and O_2 evolution transition states in the same manner. Furthermore, changes in [H_2O] more greatly changes the [CH_3CN] in comparison to changes in [C_6H_{12}]. Although CH_3CN does not explicitly

appear in the epoxidation or O_2 evolution reaction mechanisms, its presence in the electrolyte affects nonideal interactions of C_6H_{12} and H_2O with species within the electrical double layer.

S23.2. Dependency on C_6H_{12} and H_2O Activity

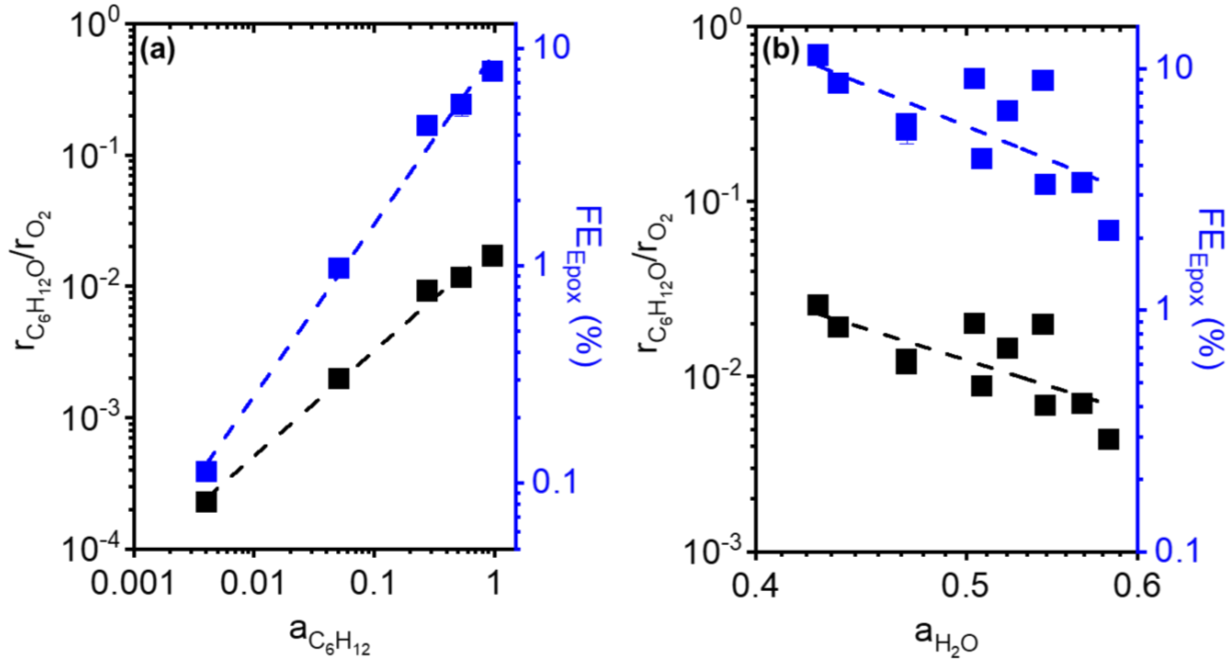

Figure S35. Epoxidation and O_2 evolution rate ratios (black) and epoxidation Faradaic efficiencies (blue) as a function of (a) C_6H_{12} activity (1.31 $V_{Fc/Fc+}$, 0.1 M $TBAClO_4$, 10 M H_2O, CH_3CN) and (b) H_2O activity (1.31 $V_{Fc/Fc+}$, 0.1 M C_6H_{12}, 0.1 M $TBAClO_4$, CH_3CN).

Figure S35 shows that both the $\frac{r_{C_6H_{12}O}}{r_{O_2}}$ and FE_{Epox} have an approximate first-order dependence on C_6H_{12} activity. The first order dependence of $\frac{r_{C_6H_{12}O}}{r_{O_2}}$ (0.8 ± 0.2) and FE_{Epox} (0.8 ± 0.2) on C_6H_{12} activity (**Figure S35a**) matches the dependence on [C_6H_{12}] and disproves the Eley-Rideal mechanism in which C_6H_{12} reacts with O_2*, as this mechanism has a predicted zero-order dependence on C_6H_{12} activity for both values. The $\frac{r_{C_6H_{12}O}}{r_{O_2}}$ (-3.9 ± 0.5) and FE_{Epox} (-3.7 ± 0.4) both have an approximate inverse fourth-order dependence on H_2O activity (**Figure S35b**). The inverse dependence of these values on H_2O activity disproves the Eley-Rideal mechanisms in which C_6H_{12} reacts with an OH*, OOH*, or O_2*, as these three mechanisms have a predicted zero-order dependence on H_2O activity for both values. The large magnitude of dependence on H_2O activity likely arises from the required assumptions to experimentally determine H_2O activity. These include assumption of equilibrium between the bulk electrolyte and electrified interface and no consideration of the non-ideality of transition states.[30] Furthermore, C_6H_{12} activity changes over the range of [H_2O] (8-18 M) studied (**Figure S18b**), and it is not straightforward to normalize $\frac{r_{C_6H_{12}O}}{r_{O_2}}$ and FE_{Epox} by C_6H_{12} activity when determining a dependence on H_2O activity, as the apparent order of C_6H_{12} activity may change over the range of 8-18 M H_2O.

S24. Product Distribution of *Cis*-Stilbene Epoxidation with H_2O

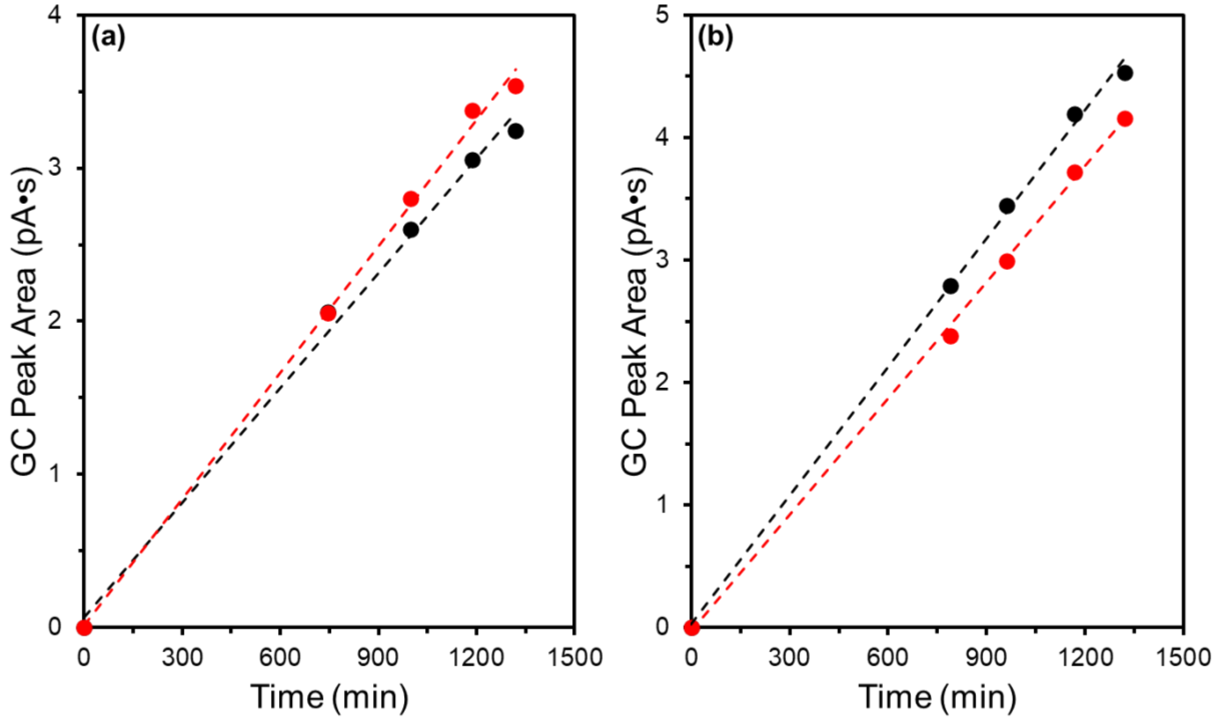

Figure S36. Formation of *cis*-stilbene oxide (black) and *trans*-stilbene oxide (red) during the epoxidation of *cis*-stilbene with initial [*cis*-stilbene] of (a) 0.05 M *cis*-stilbene and (b) 0.1 M *cis*-stilbene (1.31 $V_{Fc/Fc+}$, 0.1 M $TBAClO_4$, 10 M H_2O, CH_3CN) as functions of time.

Figure S36 shows the formation of *cis*-stilbene oxide and *trans*-stilbene oxide during the epoxidation of *cis*-stilbene at two different initial [*cis*-stilbene] as functions of time (1.31 $V_{Fc/Fc+}$, 0.05 or 0.1 M cis-stilbene, 0.1 M $TBAClO_4$, 10 M H_2O, CH_3CN). The formation profiles indicate that both epoxide isomers are primary products. *Cis*-stilbene oxide forms through a concerted mechanism that does not permit isomerization during oxygen atom transfer to the C=C bond. *Trans*-stilbene oxide forms through a stepwise homolytic mechanism in which reaction of oxygen atoms with the C=C bond allows for rotation, resulting in nearly equimolar mixtures of *trans*- and *cis*-stilbene oxides. *Cis*-stilbene epoxidation occurs exclusively through the stepwise mechanism on O_2*[31-33] and concerted mechanism on OOH*.[34-36] O* species on metal catalysts and inorganic complexes can epoxidize *cis*-stilbene through either the stepwise[37-40] or concerted mechanism.[41-43] *Cis*-stilbene oxide and *trans*-stilbene oxide form at equal rates on Au, which disproves a reactive OOH* species involved in the epoxidation mechanism and supports a stepwise mechanism with either a reactive O* or O_2* species. Kinetic results in Section 3.3. of the main text disprove the participation of a reactive O_2* species. Thus, the *cis*-stilbene product distribution results support the conclusion that Au epoxidizes alkenes primarily with O* species.

S25. Tafel Slope Analysis with Most Abundant Surface Intermediate Assumption

Tafel slopes can be predicted from proposed mechanisms because rate constants for steps containing a proton-electron transfer have a dependence on the overpotential determined by:

$$e^{\left(\frac{\alpha FE}{RT}\right)} \tag{S120}$$

Where α is the charge transfer coefficient (assumed to be 0.5), *F* is the Faraday constant (96,485 C mol^{-1}), *E* is the applied potential, *R* is the gas constant and *T* is temperature.[2] With the assumption that the O* species acts as the most abundant reactive intermediate, the rate expression for the Eley-Rideal mechanism (**Equation 9**) collapses to the following form:

$$\frac{r_{C_6H_{12}O}}{[L]} = k_5[C_6H_{12}] \tag{S121}$$

and the rate expression for the Langmuir-Hinshelwood mechanism (**Equation S116)** collapses to the following form:

$$\frac{r_{C_6H_{12}O}}{[L]} = \frac{k_5 k_6[C_6H_{12}]}{k_{-5} + k_6\beta} \tag{S122}$$

Both epoxide formation rate expressions for the mechanisms involving a reactive O* species (**Equations S121** and **S122**) have a predicted Tafel slope of ∞ as all the rate constants in the equation come from chemical steps.

Using the same assumption of a surface covered in O* species, we can simplify the O_2 formation rate expression for both the O* Eley-Rideal mechanism (**Equation 12**) and O* Langmuir-Hinshelwood mechanism (**Equation S117**) to:

$$\frac{r_{O_2}}{[L]} = k_3[H_2O]e^{\left(\frac{\alpha FE}{RT}\right)} \tag{S123}$$

Where k_3 represents an electrochemical step in both mechanisms. The predicted Tafel slope of **Equation S123** is 120 mV decade^{-1}.

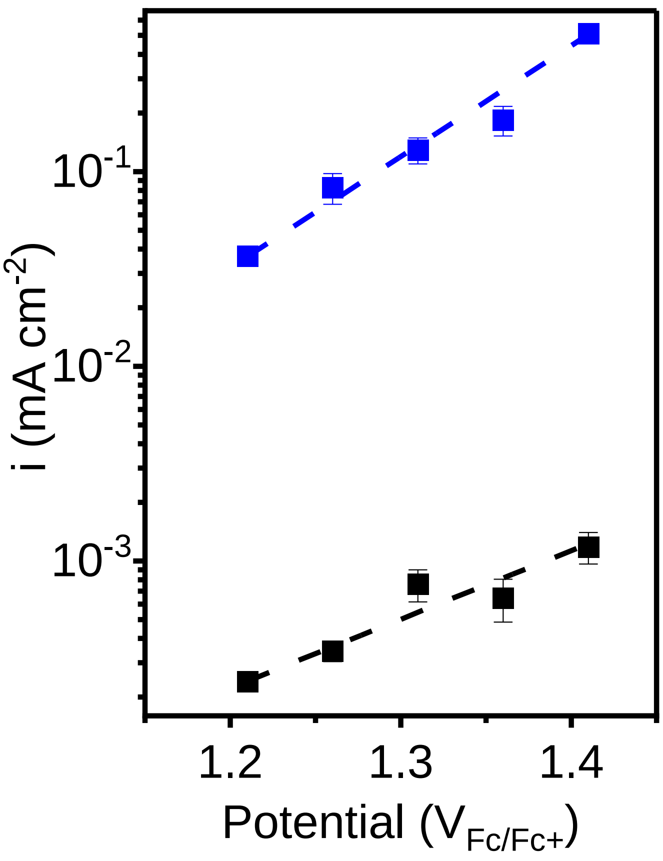

Figure S37. Partial current density of epoxidation (black) and OER (blue) as a function of potential (0.1 M C_6H_{12}, 0.1 M $TBAClO_4$, 10 M H_2O, CH_3CN).

Figure S37 depicts the partial current density of epoxidation and OER as a function of potential (0.1 M C_6H_{12}, 0.1 M $TBAClO_4$, 10 M H_2O, CH_3CN). The epoxidation and OER Tafel slopes are 273 ± 54 mV decade^{-1} and 184 ± 19 mV decade^{-1} respectively. These Tafel slopes agree with the predicted Tafel slopes, ∞ mV decade^{-1} for epoxidation and 120 mV decade^{-1} for OER, from the simplified epoxidation (**Equations S121, S122**) and OER (**Equation S123**) rate expressions when assuming an O* covered surface. The large magnitude of the epoxidation Tafel slope supports an epoxidation mechanism that contains both Faradaic and non-Faradaic steps. The epoxidation rate depends indirectly on potential, which leads to a finite Tafel slope, because the numbers of reactive O* species at steady state can increase with the applied potential. Although O* appears to be more abundant than all other surface intermediates, the fractional coverage of this species increases toward a value of unity when more positive potentials lead to greater rate constants for H_2O activation relative to C_6H_{12} epoxidation. The discrepancy between the predicted and experimental Tafel slopes likely arises from deviations of the charge transfer coefficient from 0.5. Charge transfer coefficients differ for each elementary step, depend on potential, and change with surface coverages.[44-46] Thus, the assumption that the charge transfer coefficient equals 0.5 for all steps and potentials may lead to inaccurate predictions of Tafel slopes. With these considerations, we conclude that the experimental epoxidation and OER Tafel slopes qualitatively agree with those predicted in the case of an O* covered surface.

References

1. Yoon, Y.; Yan, B.; Surendranath, Y. Suppressing Ion Transfer Enables Versatile Measurements of Electrochemical Surface Area for Intrinsic Activity Comparisons. *Journal of the American Chemical Society* **2018**, *140* (7), 2397-2400. DOI: 10.1021/jacs.7b10966.
2. Bard, A. J.; Faulkner, L. R.; White, H. S. *Electrochemical methods: fundamentals and applications*; John Wiley & Sons, 2022.
3. Chorkendorff, I.; Niemantsverdriet, J. W. *Concepts of modern catalysis and kinetics*; John Wiley & Sons, 2017.
4. Flaherty, D. W.; Bhan, A. Improving the rigor and reproducibility of catalyst testing and evaluation in the laboratory. *Journal of Catalysis* **2024**, *431*, 115408. DOI: https://doi.org/10.1016/j.jcat.2024.115408.
5. Thompson, A. C.; Vaughan, D. *X-ray data booklet*; Lawrence Berkeley National Laboratory, University of California Berkeley, CA, 2001.
6. Haiss, W.; Thanh, N. T. K.; Aveyard, J.; Fernig, D. G. Determination of Size and Concentration of Gold Nanoparticles from UV−Vis Spectra. *Analytical Chemistry* **2007**, *79* (11), 4215-4221. DOI: 10.1021/ac0702084.
7. Liu, Y.-C.; Jang, L.-Y. Relationship between Crystalline Orientations of Gold and Surface-Enhanced Raman Scattering Spectroscopy of Polypyrrole and Mechanism of Roughening Procedure on Gold via Cyclic Voltammetry. *The Journal of Physical Chemistry B* **2002**, *106* (26), 6748-6753. DOI: 10.1021/jp020644o.
8. Chinnaiah, J.; Kasian, O.; Dekshinamoorthy, A.; Vijayaraghavan, S.; Mayrhofer, K. J. J.; Cherevko, S.; Scholz, F. Tuning the Anodic and Cathodic Dissolution of Gold by Varying the Surface Roughness. *ChemElectroChem* **2021**, *8* (8), 1524-1530. DOI: https://doi.org/10.1002/celc.202100366.
9. Xu, X.; Makaraviciute, A.; Pettersson, J.; Zhang, S.-L.; Nyholm, L.; Zhang, Z. Revisiting the factors influencing gold electrodes prepared using cyclic voltammetry. *Sensors and Actuators B: Chemical* **2019**, *283*, 146-153. DOI: https://doi.org/10.1016/j.snb.2018.12.008.
10. Ramírez-Rico, D. S.; Larios-Durán, E. R. Electrochemical Study on Electrodissolution of Gold in Acidic Medium Using Chlorides as Ligands. *Journal of The Electrochemical Society* **2017**, *164* (14), H994-H1002. DOI: 10.1149/2.0351714jes.
11. Aldous, I. M.; Hardwick, L. J. Influence of Tetraalkylammonium Cation Chain Length on Gold and Glassy Carbon Electrode Interfaces for Alkali Metal–Oxygen Batteries. *The Journal of Physical Chemistry Letters* **2014**, *5* (21), 3924-3930. DOI: 10.1021/jz501850u.
12. Shimanouchi, T. *Tables of molecular vibrational frequencies*; National Bureau of Standards Washington, DC, 1972.
13. Li, C.-Y.; Dong, J.-C.; Jin, X.; Chen, S.; Panneerselvam, R.; Rudnev, A. V.; Yang, Z.-L.; Li, J.-F.; Wandlowski, T.; Tian, Z.-Q. In Situ Monitoring of Electrooxidation Processes at Gold Single Crystal Surfaces Using Shell-Isolated Nanoparticle-Enhanced Raman Spectroscopy. *Journal of the American Chemical Society* **2015**, *137* (24), 7648-7651. DOI: 10.1021/jacs.5b04670.
14. Beltramo, G. L.; Shubina, T. E.; Koper, M. T. M. Oxidation of Formic Acid and Carbon Monoxide on Gold Electrodes Studied by Surface-Enhanced Raman Spectroscopy and DFT. *ChemPhysChem* **2005**, *6* (12), 2597-2606. DOI: https://doi.org/10.1002/cphc.200500198.
15. Yang, S.; Hetterscheid, D. G. H. Redefinition of the Active Species and the Mechanism of the Oxygen Evolution Reaction on Gold Oxide. *ACS Catalysis* **2020**, *10* (21), 12582-12589. DOI: 10.1021/acscatal.0c03548.

16. Diaz-Morales, O.; Calle-Vallejo, F.; de Munck, C.; Koper, M. T. M. Electrochemical water splitting by gold: evidence for an oxide decomposition mechanism. *Chemical Science* **2013**, *4* (6), 2334-2343, 10.1039/C3SC50301A. DOI: 10.1039/C3SC50301A.
17. Yeo, B. S.; Klaus, S. L.; Ross, P. N.; Mathies, R. A.; Bell, A. T. Identification of Hydroperoxy Species as Reaction Intermediates in the Electrochemical Evolution of Oxygen on Gold. *ChemPhysChem* **2010**, *11* (9), 1854-1857. DOI: https://doi.org/10.1002/cphc.201000294.
18. Perini, N.; Ticianelli, E. A. Oxygen evolution on gold: The effects of alkali-metal cations and iron impurities from alkaline electrolytes. *Journal of Catalysis* **2019**, *378*, 277-282. DOI: https://doi.org/10.1016/j.jcat.2019.09.003.
19. Sampath, A.; Ricciardulli, T.; Priyadarshini, P.; Ghosh, R.; Adams, J. S.; Flaherty, D. W. Spectroscopic Evidence for the Involvement of Interfacial Sites in O–O Bond Activation over Gold Catalysts. *ACS Catalysis* **2022**, *12* (15), 9549-9558. DOI: 10.1021/acscatal.2c02076.
20. Nyquist, R. A.; Putzig, C. L.; Leugers, M. A. *Infrared and Raman Spectral Atlas of Inoganic Compounds and Organic Salts: Text and Explanations*; Academic Press, 1997.
21. Ratcliffe, C.; Irish, D. Vibrational spectral studies of solutions at elevated temperatures and pressures. VI. Raman studies of perchloric acid. *Canadian journal of chemistry* **1984**, *62* (6), 1134-1144.
22. Mele, C.; Bozzini, B. Silver electrodeposition from water–acetonitrile mixed solvents in the presence of tetrabutylammonium perchlorate. *Journal of Solid State Electrochemistry* **2009**, *13* (10), 1553-1559. DOI: 10.1007/s10008-008-0724-y.
23. Li, X.; Gewirth, A. A. Peroxide Electroreduction on Bi-Modified Au Surfaces: Vibrational Spectroscopy and Density Functional Calculations. *Journal of the American Chemical Society* **2003**, *125* (23), 7086-7099. DOI: 10.1021/ja034125q.
24. Zapata, F.; García-Ruiz, C. The discrimination of 72 nitrate, chlorate and perchlorate salts using IR and Raman spectroscopy. *Spectrochimica Acta Part A: Molecular and Biomolecular Spectroscopy* **2018**, *189*, 535-542. DOI: https://doi.org/10.1016/j.saa.2017.08.058.
25. Miller, A. G.; Macklin, J. W. Vibrational spectroscopic studies of sodium perchlorate contact ion pair formation in aqueous solution. *The Journal of Physical Chemistry* **1985**, *89* (7), 1193-1201. DOI: 10.1021/j100253a028.
26. Shinozaki, A.; Kawano, J.; Nagai, T. Phase Relation and Reactivity of 1-Hexene under High-Pressure and High-Temperature Conditions. *The Journal of Physical Chemistry C* **2024**, *128* (14), 5956-5963. DOI: 10.1021/acs.jpcc.3c07946.
27. Bregante, D. T.; Flaherty, D. W. Impact of Specific Interactions Among Reactive Surface Intermediates and Confined Water on Epoxidation Catalysis and Adsorption in Lewis Acid Zeolites. *ACS Catalysis* **2019**, *9* (12), 10951-10962. DOI: 10.1021/acscatal.9b03323.
28. Bregante, D. T.; Johnson, A. M.; Patel, A. Y.; Ayla, E. Z.; Cordon, M. J.; Bukowski, B. C.; Greeley, J.; Gounder, R.; Flaherty, D. W. Cooperative Effects between Hydrophilic Pores and Solvents: Catalytic Consequences of Hydrogen Bonding on Alkene Epoxidation in Zeolites. *Journal of the American Chemical Society* **2019**, *141* (18), 7302-7319. DOI: 10.1021/jacs.8b12861.
29. Kwon, O.; Ayla, E. Z.; Potts, D. S.; Flaherty, D. W. Effects of Solvent–Pore Interaction on Rates and Barriers for Vapor Phase Alkene Epoxidation with Gaseous H2O2 in Ti-BEA Catalysts. *ACS Catalysis* **2023**, *13* (9), 6430-6444. DOI: 10.1021/acscatal.3c00730.
30. Williams, K.; Limaye, A.; Weiss, T.; Chung, M.; Manthiram, K. Accounting for species' thermodynamic activities changes mechanistic interpretations of electrochemical kinetic data. *ChemRxiv Preprint* **2022**, 1.

31. Kuznetsov, M. L.; Pessoa, J. C. Epoxidation of olefins catalysed by vanadium–salan complexes: a theoretical mechanistic study. *Dalton Transactions* **2009**, (28), 5460-5468, 10.1039/B902424G. DOI: 10.1039/B902424G.
32. Mimoun, H. Do metal peroxides as homolytic and heterolytic oxidative reagents. Mechanism of the halcon epoxidation process. *Catalysis Today* **1987**, *1* (3), 281-295. DOI: https://doi.org/10.1016/0920-5861(87)80012-3.
33. Nam, W.; Lim, M. H.; Lee, H. J.; Kim, C. Evidence for the Participation of Two Distinct Reactive Intermediates in Iron(III) Porphyrin Complex-Catalyzed Epoxidation Reactions. *Journal of the American Chemical Society* **2000**, *122* (28), 6641-6647. DOI: 10.1021/ja000289k.
34. Clerici, M. G.; Ingallina, P. Epoxidation of Lower Olefins with Hydrogen Peroxide and Titanium Silicalite. *Journal of Catalysis* **1993**, *140* (1), 71-83. DOI: https://doi.org/10.1006/jcat.1993.1069.
35. Raj, N. K. K.; Ramaswamy, A. V.; Manikandan, P. Oxidation of norbornene over vanadium-substituted phosphomolybdic acid catalysts and spectroscopic investigations. *Journal of Molecular Catalysis A: Chemical* **2005**, *227* (1), 37-45. DOI: https://doi.org/10.1016/j.molcata.2004.10.005.
36. Notari, B. Microporous Crystalline Titanium Silicates. In *Advances in Catalysis*, Eley, D. D., Haag, W. O., Gates, B. Eds.; Vol. 41; Academic Press, 1996; pp 253-334.
37. Adam, W.; Roschmann, K. J.; Saha-Möller, C. R.; Seebach, D. cis-Stilbene and (1α,2β,3α)-(2-Ethenyl-3-methoxycyclopropyl)benzene as Mechanistic Probes in the MnIII(salen)-Catalyzed Epoxidation: Influence of the Oxygen Source and the Counterion on the Diastereoselectivity of the Competitive Concerted and Radical-Type Oxygen Transfer. *Journal of the American Chemical Society* **2002**, *124* (18), 5068-5073. DOI: 10.1021/ja0177206.
38. Linde, C.; Koliaï, N.; Norrby, P.-O.; Åkermark, B. Experimental Evidence for Multiple Oxidation Pathways in the (salen)Mn-Catalyzed Epoxidation of Alkenes. *Chemistry – A European Journal* **2002**, *8* (11), 2568-2573. DOI: https://doi.org/10.1002/1521-3765(20020603)8:11<2568::AID-CHEM2568>3.0.CO;2-Z.
39. Singh, K. K.; Tiwari, M. k.; Dhar, B. B.; Vanka, K.; Sen Gupta, S. Mechanism of Oxygen Atom Transfer from FeV(O) to Olefins at Room Temperature. *Inorganic Chemistry* **2015**, *54* (13), 6112-6121. DOI: 10.1021/ic503053q.
40. Hughes, M. D.; Xu, Y.-J.; Jenkins, P.; McMorn, P.; Landon, P.; Enache, D. I.; Carley, A. F.; Attard, G. A.; Hutchings, G. J.; King, F.; et al. Tunable gold catalysts for selective hydrocarbon oxidation under mild conditions. *Nature* **2005**, *437* (7062), 1132-1135. DOI: 10.1038/nature04190.
41. Choe, C.; Yang, L.; Lv, Z.; Mo, W.; Chen, Z.; Li, G.; Yin, G. Redox-inactive metal ions promoted the catalytic reactivity of non-heme manganese complexes towards oxygen atom transfer. *Dalton Transactions* **2015**, *44* (19), 9182-9192, 10.1039/C4DT03993A. DOI: 10.1039/C4DT03993A.
42. Dobson, J. C.; Seok, W. K.; Meyer, T. J. Epoxidation and catalytic oxidation of olefins based on a RuIV:O/RuII-OH2 couple. *Inorganic Chemistry* **1986**, *25* (10), 1513-1514. DOI: 10.1021/ic00230a001.
43. Wang, B.; Lee, Y.-M.; Tcho, W.-Y.; Tussupbayev, S.; Kim, S.-T.; Kim, Y.; Seo, M. S.; Cho, K.-B.; Dede, Y.; Keegan, B. C.; et al. Synthesis and reactivity of a mononuclear non-haem cobalt(IV)-oxo complex. *Nature Communications* **2017**, *8* (1), 14839. DOI: 10.1038/ncomms14839.

44. Guidelli, R.; Compton, R. G.; Feliu, J. M.; Gileadi, E.; Lipkowski, J.; Schmickler, W.; Trasatti, S. Defining the transfer coefficient in electrochemistry: An assessment (IUPAC Technical Report). *Pure and Applied Chemistry* **2014**, *86* (2), 245-258. DOI: doi:10.1515/pac-2014-5026 (acccessed 2024-08-26).
45. Savéant, J.-M.; Tessier, D. Variation of the electrochemical transfer coefficient with potential. *Faraday Discussions of the Chemical Society* **1982**, *74* (0), 57-72, 10.1039/DC9827400057. DOI: 10.1039/DC9827400057.
46. Mareček, V.; Samec, Z.; Weber, J. The dependence of the electrochemical charge-transfer coefficient on the electrode potential: Study of the Fe(CN)63−/Fe(CN)64− redox reaction on polycrystalline Au electrode in KF solutions. *Journal of Electroanalytical Chemistry and Interfacial Electrochemistry* **1978**, *94* (3), 169-185. DOI: https://doi.org/10.1016/S0022-0728(78)80312-X.
